# Metagenomic Analyses of Water Samples of Two Urban Freshwaters in Berlin, Germany, Reveal New Highly Diverse Invertebrate Viruses

**DOI:** 10.3390/microorganisms12112361

**Published:** 2024-11-19

**Authors:** Roland Zell, Marco Groth, Lukas Selinka, Hans-Christoph Selinka

**Affiliations:** 1 Section of Experimental Virology, Institute for Medical Microbiology, Jena University Hospital, Friedrich Schiller University, 07740 Jena, Germany; 2 CF Next Generation Sequencing, Leibniz Institute on Aging - Fritz Lipmann Institute, 07745 Jena, Germany; 3 Section II 1.4 Microbiological Risks, Department of Environmental Hygiene, German Environment Agency, 14195 Berlin, Germany

**Keywords:** riverine ecosystems, freshwater RNA virome, modularity of viruses, functional virus domains, virus taxonomy, virus hallmark genes

## Abstract

In an attempt to explore the RNA viromes of two German rivers, we searched the virus particle contents of one 50 L water sample each from the Teltow Canal and the Havel River for viruses assumed to infect invertebrates. More than 330 complete and partial virus genomes up to a length of 37 kb were identified, with noda-like and reo-like viruses being most abundant, followed by bunya-like and birna-like viruses. Viruses related to the *Permutotetraviridae*, *Nidovirales*, *Flaviviridae*, *Rhabdoviridae and Chuviridae* as well as the unclassified Jῑngmén virus and Negev virus groups were also present. The results indicate a broad extent of recombinant virus genomes, supporting the concept of the modularity of eukaryotic viruses. For example, novel combinations of genes encoding replicase and structural proteins with a jellyroll fold have been observed. Less than 35 viruses could be assigned to existing virus genera. These are (i) an avian deltacoronavirus which was represented by only one short contig, albeit with 98% similarity, (ii) a seadornavirus and a rotavirus, and (iii) some 30 nodaviruses. All remaining viruses are novel and too diverse for accommodation in existing genera. Many of the virus genomes exhibit ORFans encoding hypothetical proteins of up to 2000 amino acids without conserved protein domains.

## 1. Introduction

Freshwater macroinvertebrate fauna, which is mainly comprised of arthropods, gastropods, flatworms, nematodes and annelids, is an essential component of all freshwater ecosystems. As macroinvertebrates occupy the benthal, pelagial and riparian/littoral zones, they influence the primary productivity of a waterbody, promote the decomposition of detritus and constitute a source of food for fish [1]. In lakes and ponds and along the river courses, a mosaic of patches and habitats can be found, and—depending on the local conditions—the many lentic and lotic habitats differ in both their composition of invertebrate communities and in the fluctuation of population densities across spatiotemporal scales. The invertebrate assemblage is governed by a complex melange of abiotic factors and biotic interactions, like seasonality, food resources, predators and pathogens, and is further affected by anthropogenic disturbances, e.g., physical habitat modification, deforestation, pollution, over-harvesting and global climate change [1,2,3]. Freshwater invertebrates are highly diverse, as about 90,000 species have been formally described and many more remain to be discovered [4].

Virus infection has been shown to play a significant role in bacterial mortality in marine and freshwater environments (for a review, see [5]) and may also contribute—directly or indirectly via hyperparasitism—to the control of the health and reproductive capacity of invertebrate populations [6,7]. The extent of this function, however, is presently unclear. Research from the past few decades revealed viruses to be an important constituent of plankton [5,8]. Marine virioplankton, which is better investigated than the virioplankton of streams and lakes, is known to play a role in the mortality of single-cell organisms and to influence the food webs and nutrient cycles of marine ecosystems [9,10]. Viral abundance in lakes and rivers often exceeds that of marine sites in absolute numbers and in virus-to-bacteria ratios [5,11,12,13]. Many viruses closely related to marine protist-infecting viruses have been detected in freshwater samples and in tissues of terrestrial animals and plants as well as in various fecal samples [14,15,16,17,18,19,20,21,22]. Although still underexplored, the relevance of virioplankton to freshwater bacteria and protists is considered similar to that of their marine counterparts [13,23]. The impact of freshwater viromes on aquatic vertebrate and invertebrate fauna is barely understood except for a few investigations on crustacea and mussels and the descriptions of occasional mass die-offs of fishes, shrimps and bivalves (e.g., [7,19,24,25,26,27,28,29,30,31]; for reviews, see [32,33]).

Metagenomics is presently the state-of-the-art approach to surveying the viral diversity of environmental water samples. In fact, the elucidation of the virosphere enormously benefitted from recent advancements in nucleic acid sequencing technologies. These methods allow an unbiased sequencing of uncultured virus genomes (UViGs) and resulted in the identification of more than 750,000 UViGs—a tiny proportion of the vast global virosphere [34,35]. As a consequence, a virus classification system comprising 15 hierarchical ranks was established [36]. Moreover, the creation of new taxonomic ranks based on UViG sequences has been demonstrated to be feasible and was endorsed by the Executive Committee of the International Committee on Taxonomy of Viruses [37,38]. Besides the needful cataloging of the virosphere, tasks for virologists resulting from the enormous increase of knowledge were only recently addressed [39].

The objective of our project is to generate a meticulous description of the viromes of two German rivers, the Teltow Canal and the Havel River in Berlin, Germany. Both freshwater bodies are connected (see map in ref. [40]) and have been selected for our study as they are part of a multi-year environmental surveillance program by the German Environment Agency aimed at monitoring environmentally relevant human pathogenic viruses in surface water. The Havel River has a near-natural river course and is extensively used for recreational activities in the summer months, whereas the Teltow Canal is an artificial connection between the rivers Spree and Havel and runs through densely populated districts in the southwest of Berlin. The Teltow Canal is burdened with the discharges of local wastewater treatment plants as well as with drain water effluents after heavy rainfalls. Viruses detected in water samples are characterized on the basis of hallmark genes encoding characteristic viral protein domains. Part of the results have been published already [21,22,40,41]. The present paper focuses on environmental viruses assumed to infect the invertebrate fauna of these rivers. For this, we describe some 325 viruses with birna-like, flavi-like, noda-like, permutotetra-like, rhabdo-like and chu-like RdRp as well as viruses of the negevirus group (*Martellivirales*), and viruses with similarity to the many families of *Bunyaviricetes*, *Nidovirales* and *Reovirales*. Invertebrate viruses with similarity to members of *Hepelivirales* and *Picornavirales* were excluded (compare [21,22,41]).

## 2. Materials and Methods

### 2.1. Sampling and Virus Enrichment

Two 50 L water samples (sample IDs MR233-17 and MR644-18) were collected in Berlin, Bäkebrücke (Teltow Canal, site coordinates: 52°26′03″ N, 13°18′57″ E), on 18 July 2017, and Berlin, Heerstrasse (Havel River, site coordinates: 52°30′46″ N 13°12′14″ E), on 28 June 2018. Both samples were used for virus enrichment according to the method of Wyn-Jones et al. [42]. As described previously [21,22,40,41], the samples were each partitioned into five 10 L aliquots that were vigorously stirred for 20 min to homogenize suspended detritus. After adjusting pH to 3.5 with hydrochloric acid, virus particles were adsorbed to glass wool, washed and eluted with 3% beef extract/0.05 M glycine buffer pH 9.5. The alkaline eluates were neutralized with sodium hydroxide solution. Next, residual detritus and bacteria of the eluates were removed by filtering (0.45 µm). Thereafter, virus particles were sedimented by ultracentrifugation (100,000× *g*, 2.5 h at 4 °C). In a final step, 500 µL phosphate-buffered saline was added and the sediments were redissolved using a ball mill. Both virus suspensions were stored at −80 °C.

### 2.2. RNA Preparation and Illumina Sequencing

We used the QIAamp Viral RNA mini kit (Qiagen, Hilden, Germany) according to the manufacturer’s instructions for RNA extraction. The libraries were prepared as follows: Teltow Canal sample: 450 ng of total RNA was introduced into Illumina’s TruSeq stranded total RNA library preparation kit combined with the Ribo-Zero Gold rRNA Removal Kit according the manufacturer’s descriptions (Illumina, San Diego, CA, USA); Havel River sample: 100 ng was introduced into Illumina’s TruSeq stranded mRNA library preparation kit. In order to address all RNA molecules (not only polyadenylated RNA), the protocol was adapted as follows: RNA was precipitated using isopropanol and resolved in the Fragment, Prime, Finish Mix (FPF). From this step onward, the manufacturer’s protocol was followed (p20, step 12, TruSeq Stranded mRNA Sample Preparation Guide, Part # 15031047 Rev. E, Illumina, San Diego, CA, USA). Quality check and quantitation of the libraries were performed with the 2100 Bioanalyzer instrument and the DNA 7500 kit (Agilent Technologies, Waldbronn, Germany). Paired-end Illumina sequencing (2 × 150 bp) was done on a HiSeq 2500 platform using the rapid run mode.

### 2.3. Sequence Data Processing and Sequence Analyses

Using bcl2FastQ v2.19.1.403 (Illumina), the sequence data were extracted in FastQ format. Adapter sequences were removed with Cutadapt v1.8.3 software [43] and duplicons were extracted as described previously [41]. As a result, 70,018,635 read pairs from the Teltow Canal sample and 51,902,006 read pairs from the Havel River sample were obtained for de novo assembly with clc_assembler v5.2.1 (Qiagen) utilizing the parameters -p fb ss 50 500, and metaSPAdes v3.15.3 [44] using standard parameters (-k auto). The Teltow Canal sample yielded 537,529 contigs greater than 200 nucleotides with the clc_assembler, and 1,314,849 scaffolds with metaSPAdes. The Havel River sample yielded 162,082 clc contigs and 388,367 scaffolds. Final sequences were generated by manual curation, i.e., through linking of the overlapping apt contigs and scaffolds.

Sequence data were analyzed in a 2-step procedure. First, scaffolds from metaSPAdes and contigs from the clc_assembler were used to search an in-house virus protein database compiled from all NCBI GenBank entries with the Taxonomy ID 10239 using DIAMOND v2.0.10 [45]. Second, DIAMOND hits were confirmed with BLAST+ v2.13.0 (https://ftp.ncbi.nlm.nih.gov/blast/executables/blast+/2.13.0/ accessed 10 September 2024) using the BLASTp, tBLASTx and BLASTn tools. If appropriate, specific BLAST searches were conducted with reference sequences downloaded from GenBank. Further, protein domains were predicted using the NCBI web search tools BLASTp suite (https://blast.ncbi.nlm.nih.gov/Blast.cgi accessed 10 September 2024) and the Pfam conserved domain database (CDD; https://www.ncbi.nlm.nih.gov/Structure/cdd/wrpsb.cgi accessed 10 September 2024). For sequence alignments, ClustalW or Muscle implemented in Mega version X [46] was used. Alignments were adjusted manually if necessary. Phylogenetic trees were inferred with IQ-TREE 2.1.3 for Windows [47], using the automatic model selection option (ModelFinder) of IQ-TREE for the identification of the best-fit substitution models. Branch support was assessed using either the standard non-parametric bootstrap analysis with 1000 replications for simple datasets, or the ultrafast bootstrap approximation UFBoot2 with 10,000 replications for complex alignments [48].

For provisional virus assignment, the current virus taxonomy (2023–2024 release) based on the most recent Master Species List #39 (https://ictv.global/msl; accessed on 20 September 2024) and the most recent Virus Metadata Resource spreadsheet (https://ictv.global/vmr/current; released on 17 May 2024) were used.

## 3. Results

### 3.1. Noda-like Viruses

**Figure 1:**
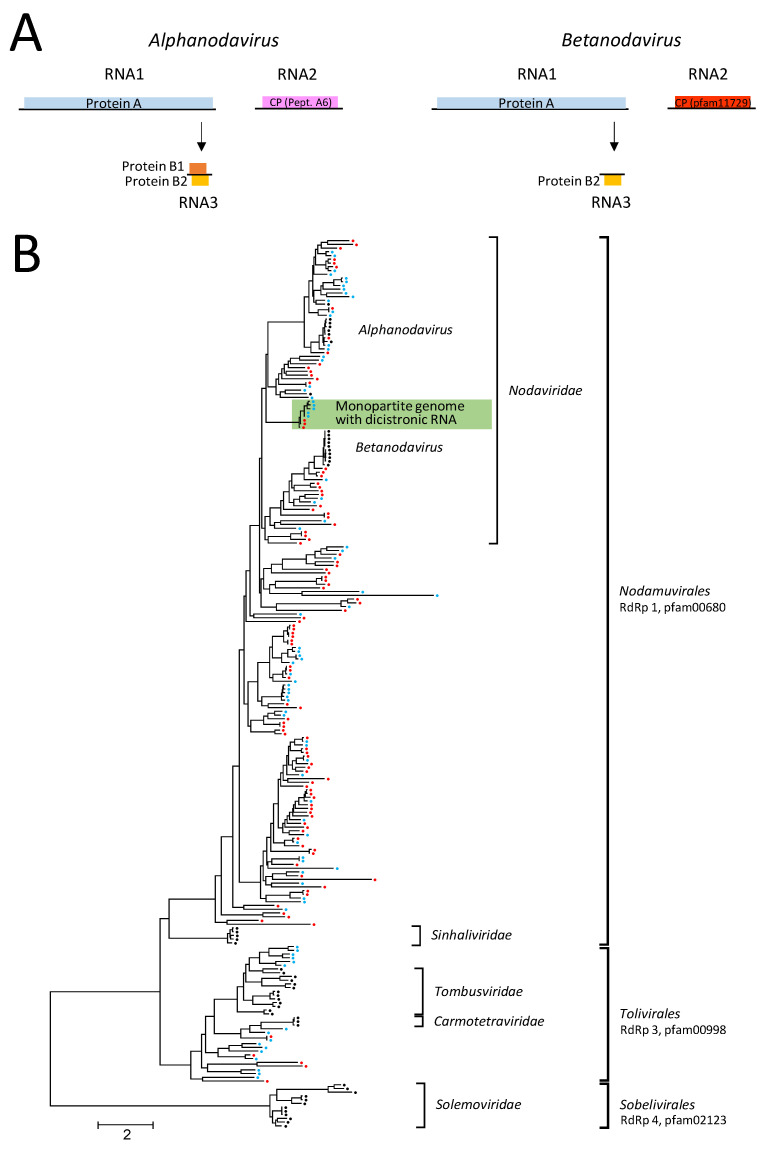
**Nodaviruses and related viruses.** (**A**) Genome layout of alphanodavirus (**left panel**) and betanodavirus (**right panel**). Open reading frames are indicated by coloured boxes. Subgenomic RNA3 is generated from RNA1 by transcription. Whereas proteins A and B2 are homologous in alpha- and betanodaviruses, capsid proteins (CP) are different. (**B**) The RdRp sequence of 83 Teltow Canal noda-LVs (red dots), 16 Havel River noda-LVs (red dots), 51 classified reference strains (black dots) of the *Nodaviridae*, *Sinhaliviridae*, *Carmotetraviridae*, *Solemoviridae* and *Tombusviridae* families, and 88 unclassified viruses (blue dots) were aligned with MEGA and used for maximum likelihood tree inference with IQ-TREE 2 (optimal substitution model: Q.pfam+F+R8). Square brackets indicate branches with families and orders. The tree was arbitrarily rooted with solemovirus sequences. The bar indicates amino acid substitutions per site. Details of the phylogenetic tree are presented in Figure S1.

Besides dicistro-like viruses, nodaviruses constitute the largest virus group in our datasets that infects invertebrates [21,22]. Nodaviruses are positive-stranded RNA viruses with a bipartite genome [49]. Both genome segments are encapsidated in a non-enveloped icosahedral T = 3 capsid. RNA 1 (ca. 3.1 kb) encodes protein A, a large protein with methyltransferase (MeTr) and RNA-dependent RNA polymerase (RdRp) domains, whereas RNA 2 (ca. 1.4 kb) codes for the capsid protein (CP). Nodavirus-infected cells contain a third subgenomic RNA (387 nt) which is derived from RNA 1 (Figure 1A). It encodes an RNAi suppressor (protein B2); some nodaviruses also express a protein named B1 with unknown function. The *Nodaviridae* family consists of two genera, *Alphanodavirus* and *Betanodavirus*, with five and four species, respectively, plus a number of related, unclassified viruses [50]. Both genera are distinguished by two characteristic features: (i) Insects are hosts of alphanodaviruses, whereas betanodaviruses infect fishes. (ii) Alphanodaviruses express a CP precursor named protein α which has similarity to the peptidase A6 (pfam01829). The maturation of alphanodavirus provirions requires autocatalytic cleavage of the protein α to yield the β and γ subunits. In contrast, the betanodavirus CP (pfam11729) has no proteolytic activity but has similarity to other CPs with a jellyroll fold.

DIAMOND assigned 437 MetaSPAdes scaffolds and 578 clc contigs to the *Nodaviridae* family. In addition, more than 150 clc contigs but no scaffolds were correlated with unclassified noda-like viruses. We confirmed 108 noda-like sequences from the Teltow Canal and 18 sequences from the Havel River by BLASTp. Of these 126 viruses, 106 sequences contained an alpha-like type-1 RdRp (protein superfamily pfam00680, cdd subfamily cd23173) characteristic of *Nodaviridae* and 10 sequences contained only a methyltransferase sequence (pfam19222). The lengths of RNA1 ranged up to 3.5 kb. The RdRp tree included 99 Teltow Canal and Havel River sequences which were suited for an alignment, plus 23 *Nodamuvirales* reference strains, 67 unclassified noda-like viruses as well as 28 virus sequences of the *Tolivirales* and *Sobelivirales* orders, and 21 unclassified tombus-like sequences serving as the outgroup (Figure 1B and Figure S1). Several viruses from the Teltow Canal and the Havel River but also unclassified sequences which were retrieved from GenBank revealed unusual features, e.g., monopartite genomes with dicistronic RNAs but no protein B2-encoding sequence (for a compilation, see Table S1). The first orf coded for RdRp, whereas the second orf encoded either a CP with similarity to peptidase A6 (alphanodavirus-like CP, pfam01829; n = 4) or a CP with an S-domain (pfam00729; n = 3) or a divergent hypothetical protein without conserved domains (ORFan; n = 23). Three other Teltow Canal noda-like viruses, namely TC-noda-LV-2, -19 and -29, revealed an unusually long nonstructural polyprotein; its C-terminal part (up to 900 amino acids) showed no similarity to known proteins.

**Figure 2:**
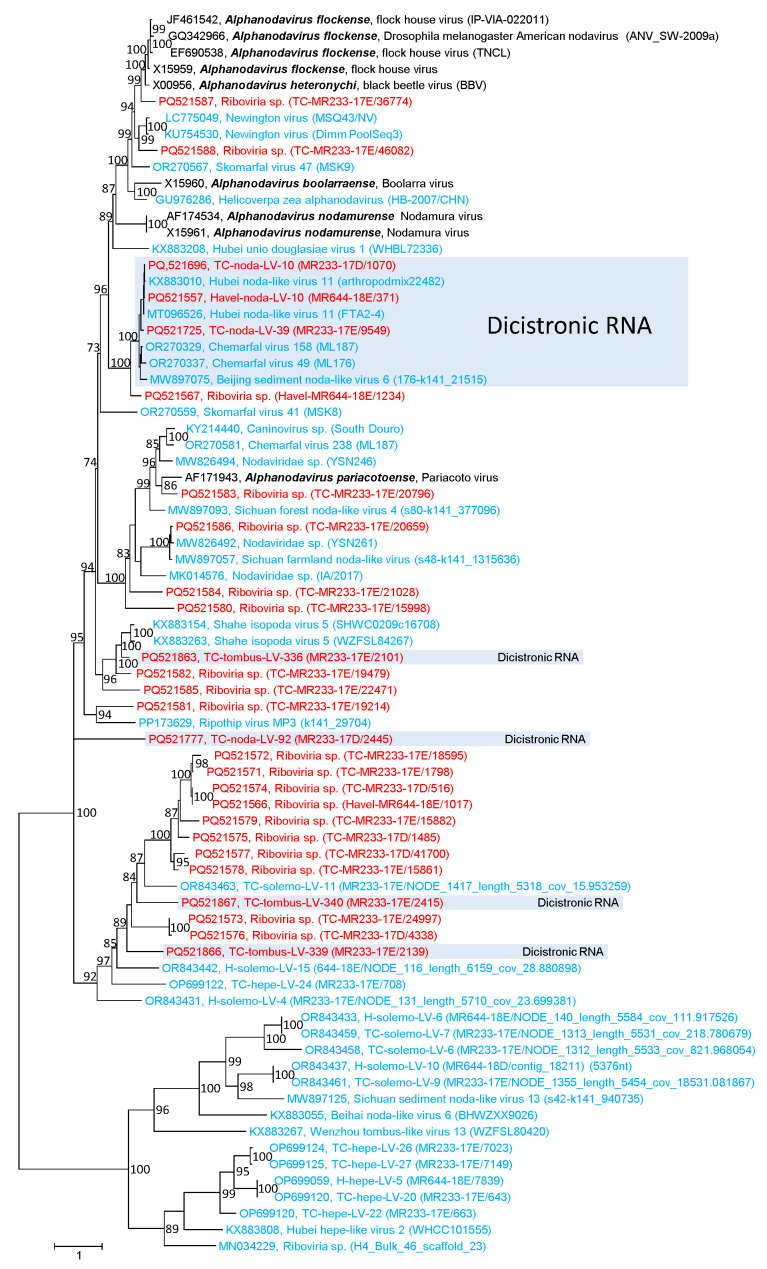
**Phylogenetic analysis of capsid proteins with similarity to peptidase A6.** A total of 76 sequences with similarity to peptidase A6 (pfam01829) were aligned and used for tree inference with IQ-TREE 2; optimal substitution model: Q.pfam+F+R5. Presented is the unrooted maximum likelihood tree. Numbers at nodes indicate bootstrap support obtained with 10,000 ultrafast replications. The scale bar indicates the number of substitutions per site. Colour code: red, virus sequences of this study from Teltow Canal (TC) and Havel River (H); blue, unclassified viruses; black, classified reference viruses. Presented are GenBank accession numbers, species names (printed in bold and italics), virus names and strain designations/sequence identifiers (in round brackets). Viruses with dicistronic RNAs are indicated.

In order to investigate the structural proteins of viruses with noda-like RdRp, we searched our scaffolds/contigs for sequences with similarity to betanodavirus CP (pfam11729) as well as peptidase A6 (Figure 2 and Figure S2). As a result, we identified five short sequences (0.4–1.8 kb) with betanoda-like CPs, which is compatible with the assumption that they belong to a segmented betanodavirus genome, and three dicistronic sequences encoding a CP (pfam11729) plus an ORFan (Figure S2). In addition, we observed three dicistronic virus genomes with noda-like RdRp but with a CP of protein family pfam00729, and six genomes with noda-like RdRp and ORFans. Moreover, 13 subgenomic CP sequences were detected without additional sequence information. These sequences did not allow for reliable assignment to a virus family—hence their designation “*Riboviria* sp.” (Figure S2). Among the viruses with peptidase A6-like CPs, four viruses showed a dicistronic genome with noda-like RdRp and three showed a dicistronic genome with tombus-like RdRp. Nineteen untypeable sequences had only a peptidase A6-like CP (Figure 2).

### 3.2. Reo-like Viruses

The second largest virus group in our datasets is reo-like viruses. The order *Reovirales* comprises two families, *Sedoreoviridae* and *Spinareoviridae*, with six and nine genera, respectively, and 97 species altogether (https://ictv.global/report/sedoreoviridae, https://ictv.global/report/spinareoviridae accessed 10 September 2024; [51,52]). Reoviruses are double-stranded RNA viruses with segmented genomes. Nine to twelve RNA segments are packed in non-enveloped, icosahedral capsids of 50–100 nm. The sizes of the genome segments range from 0.6 to 5.8 kb with a total length of 18–29 kb. Most segments encode only one protein. The reovirus capsid consists of 1–3 concentric protein layers with a T = 1/pseudo T = 2 symmetry of the inner layer and a T = 13 symmetry of the outer and middle layers where present [53]. The proteins of the middle and inner layers have various enzymatic activities (RdRp, NTPase, helicase, methyltransferase, transmethylase). Transcription and replication occur within a double-layered particle or a core particle consisting of the inner protein layer only. Both particles are embedded in a cytosolic viroplasm [53]. Viruses of the order *Reovirales* infect a wide range of hosts including mammals, birds, arthropods and plants.

DIAMOND assigned 180 MetaSPAdes scaffolds and 253 clc contigs to the *Reovirales* order, of which at least 93 scaffolds (52%) and 111 contigs belonged to rotaviruses of the *Sedoreoviridae* family. We confirmed and further analyzed sequences of *Rotavirus* (59 scaffolds, 80 contigs), *Seadornavirus* (15 scaffolds, 16 contigs), and other reo-like viruses with lengths up to 4.9 kbp (26 scaffolds, 36 contigs).

**Figure 3:**
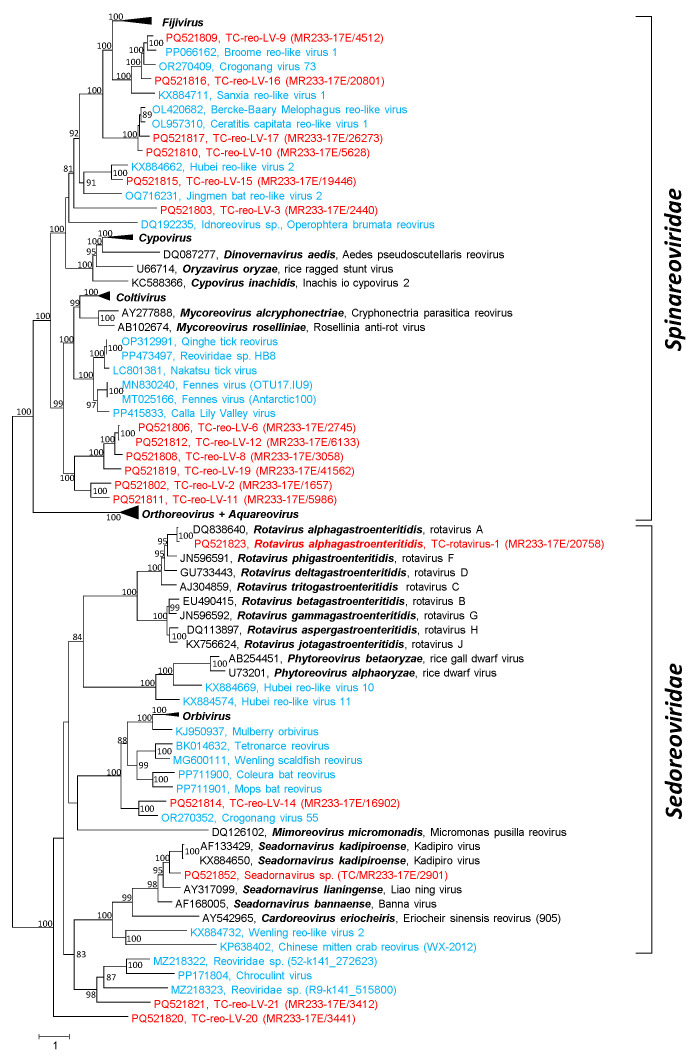
**Phylogenetic analysis of reo-like RdRp.** A total of 96 RdRp sequences of representative members of the *Spinareoviridae* and *Sedoreoviridae* families were aligned and used for tree inference with IQ-TREE 2; optimal substitution model: VT+F+R8. Presented are GenBank acc. nos., species names (printed in bold and italics), virus names and strain designations if available (in round brackets). Square brackets indicate families. Numbers at nodes indicate bootstrap support greater than 75% obtained after 10,000 ultrafast replications. The bar indicates amino acid substitutions per site. Colour code: red, viruses of the Teltow Canal; blue, unclassified reo-like viruses; black, classified reference viruses. Some clades representing genera of the *Spinareoviridae* family were condensed. Details of the phylogenetic tree are presented in Figure S3.

BLAST searches revealed the presence of all rotavirus segments. Further, nine of the twelve genome segments of a novel seadornavirus, plus scaffolds of two other seadornaviruses and partial genomes of at least 15 other reo-like viruses, were detected. A phylogenetic analysis of the RdRp sequences substantiated the rotavirus and seadornavirus sequences and revealed 3 highly divergent viruses of the *Sedoreoviridae* family and 12 novel spinareoviruses (Figure 3 and Figure S3). Six of the novel spinareo-like viruses form a strongly supported clade with distant relation to coltiviruses and mycoreoviruses. One of the sedoreo-like viruses, TC-reo-LV–14, is closely related to Crogonang virus 55, another unclassified virus from freshwater mussels [19]. Both viruses have similarity to orbiviruses. Inconsistent clustering was observed with sequences of orthoreoviruses and aquareoviruses; sequences of both genera did not group into separate branches. Likewise, the RdRp sequence of Inachis io cypovirus 2 (*Cypovirus inachidis*) did not cluster with the remaining cypoviruses (Figure 3).

### 3.3. Bunya-like Viruses

We investigated 31 sequences which were assigned by DIAMOND and BLAST to the bunyavirus group. The present ICTV taxonomy arranges the many bunyaviruses of the *Bunyaviricetes* class into two orders, *Elliovirales* and *Hareavirales*, 15 families, 69 genera and 592 species (https://ictv.global/taxonomy, accessed 10 September 2024). This taxonomy emphasizes the phylogenetic relations of RdRp and does not reflect the complex ecologic virus–host interactions of the many bunyaviruses, which may involve vectors and a change of hosts. The broad host range includes vertebrates (fish, reptiles, birds, mammals), various invertebrates, plants, fungi and protists. Bunyaviruses are enveloped, negative-stranded RNA viruses with segmented genomes. The virions are pleomorphic and have an appearance ranging from spherical to filamentous. Virions contain 1 to 10 linear RNA segments covered with nucleoprotein. The ribonucleoprotein molecules appear circular due to short complementary sequences at the 5′ and 3′ termini of the RNA which allow the formation of panhandle structures [54].

**Figure 4:**
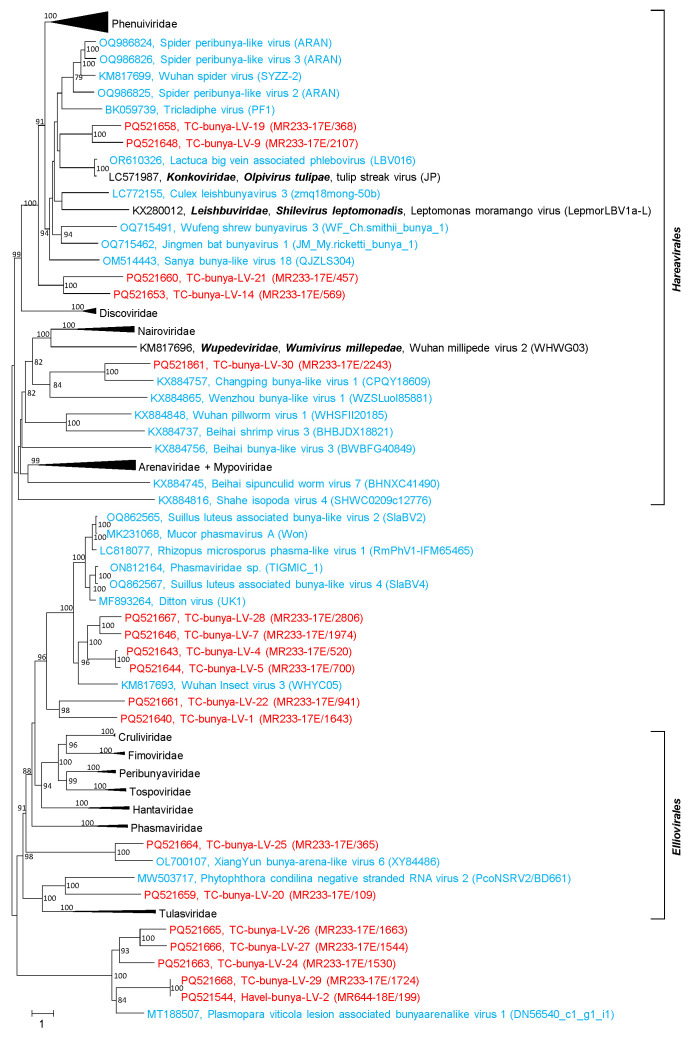
**Phylogenetic analysis of bunya-like RdRp.** The RdRp sequences of 92 viruses were aligned with MEGA and used for tree inference with IQ-TREE 2 (optimal substitution model: VT+F+R8). Presented are GenBank acc. nos., species names (printed in bold and italics), virus names and strain designations if available (in round brackets). Square brackets indicate orders. Numbers at nodes indicate bootstrap support greater than 75% obtained after 10,000 ultrafast replications. The bar indicates amino acid substitutions per site. Colour code: red, sequences of viruses from Teltow Canal and Havel River; blue, unclassified viruses; black, classified reference viruses. Details of the phylogenetic tree are presented in Figure S4. Note: members of the *Arenaviridae* and *Mypoviridae* families cluster together.

DIAMOND assigned 28 scaffolds and 149 contigs of the Teltow Canal dataset to the *Bunyaviricetes* class and unclassified bunya-like viruses, respectively. All but one of the scaffolds were confirmed by BLAST, whereas only 31 of the 149 contigs had sizes greater than 1 kb and were further studied. The Havel River datasets contained only two bunyavirus scaffolds and four contigs. The analysis revealed that none of the new sequences belonged to an acknowledged species. Eighteen sequences were suited for the phylogenetic analysis of RdRp. The phylotree (Figure 4 and Figure S4) revealed five sequences of the *Hareavirales* order and eight sequences of the *Elliovirales* order. Five sequences were too divergent for an assignment to either order and were related to Plasmopara viticola lesion associated bunyaarenalike virus 1. The results suggest the creation of at least 10 new virus taxa (species/genera/families).

### 3.4. Birna-like Viruses

**Figure 5:**
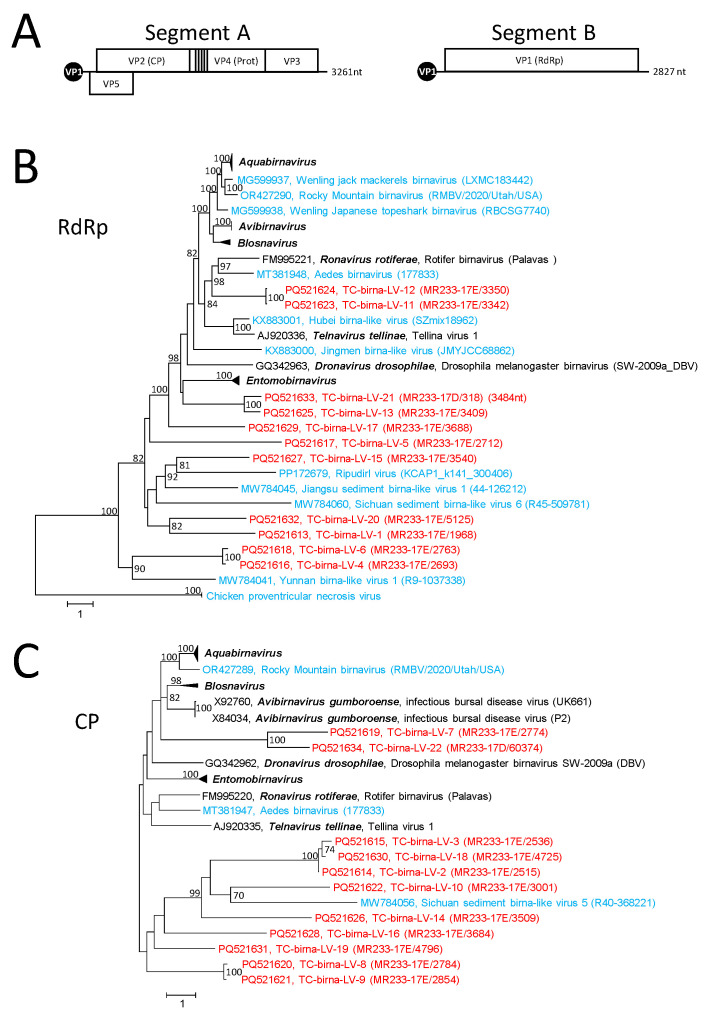
***Birnaviridae* and related viruses.** (**A**) Genome layout of birnaviruses. The bi-segmented genome has three open reading frames which are indicated by boxes. Both segments are covalently attached to VP1 (RdRp). Phylogenetic analysis of birna-like RdRp (**B**) and capsid protein VP2 (**C**). A total of 59 sequences of birna-like RdRP (**B**) and 40 sequences of the capsid protein VP2 (**C**) were aligned with MEGA and used for tree inference with IQ-TREE 2; optimal substitution model: LG+F+R6 (**B**) and Q.pfam+F+R4 (**C**). Presented are GenBank acc. nos., species names (printed in bold and italics), virus names and strain designations if available (in round brackets). Numbers at nodes indicate bootstrap support greater than 75% obtained after 10,000 ultrafast replications. The bar indicates amino acid substitutions per site. The tree was arbitrarily rooted with sequences of chicken proventricular necrosis virus. Branches representing members of the birnavirus genera were condensed. Details of the phylogenetic trees are presented in Figure S5A,B. Colour code: red, sequences of the Teltow Canal birna-like viruses (TC-birna-LV); blue, unclassified viruses; black, classified reference viruses.

Twenty-seven birna-like virus sequences from the Teltow Canal were identified in the present study. Birnaviruses are double-stranded RNA viruses with two genome segments packed in a non-enveloped, icosahedral capsid with T = 13 symmetry [55]. Segment A (2.9–3.6 kbp) exhibits two overlapping orfs. The large orf 2 encodes a polyprotein which is autocatalytically processed into a capsid protein with two jellyroll domains (VP2), a protease (VP4), an RNA-binding protein (VP3) and three to four small peptides (Figure 5A). Another protein, VP5, is encoded by the small orf 1. Segment B (2.6–3.25 kbp) encodes VP1 which is RdRp. The replication and transcription of capped mRNA without a poly(A) tail occur in the cytoplasm. The N-terminal serine residue of RdRp is covalently attached to the 5′-end of the positive RNA strand, but unbound RdRp molecules are also present in the virus particle. The birnavirus RdRp has a palm domain with a permuted order of the highly conserved active site motifs DN–x_12-20_–DxxxxE–x_62-79_–SGxxxTxxxN (C–A–B) [56,57].

DIAMOND assigned 47 scaffolds/53 contigs to birnaviruses. Twenty-seven scaffolds of sizes greater than 1 kb were further analyzed. Fourteen sequences representing segment A and 13 segment B sequences were suited for phylogenetic analyses of the VP1 (RdRp) and VP2 (CP) proteins (Figure 5B,C and Figure S5A,B). None of these sequences clustered with any of the seven acknowledged genera of *Birnaviridae*. Presumably, eight new birnavirus genera have to be created to accommodate the Teltow Canal birna-like viruses in the present *Birnaviridae* taxonomy. It is worth noting that the sequence of TC-birna-LV–1 has an unusual length of 4.5 kbp and encodes two proteins. It is unclear whether the 5′-end of orf 1 is complete. It codes for a hypothetical protein of at least 35 kDa without similarity to any protein deposited in GenBank. Orf 2 encodes a divergent RdRp with characteristic hallmarks of a birnavirus VP1. In addition, 6 of 14 segment A and 8 of 13 segment B sequences were greater than the RNAs of the acknowledged birnaviruses.

### 3.5. Permutotetra-like Viruses

**Figure 6:**
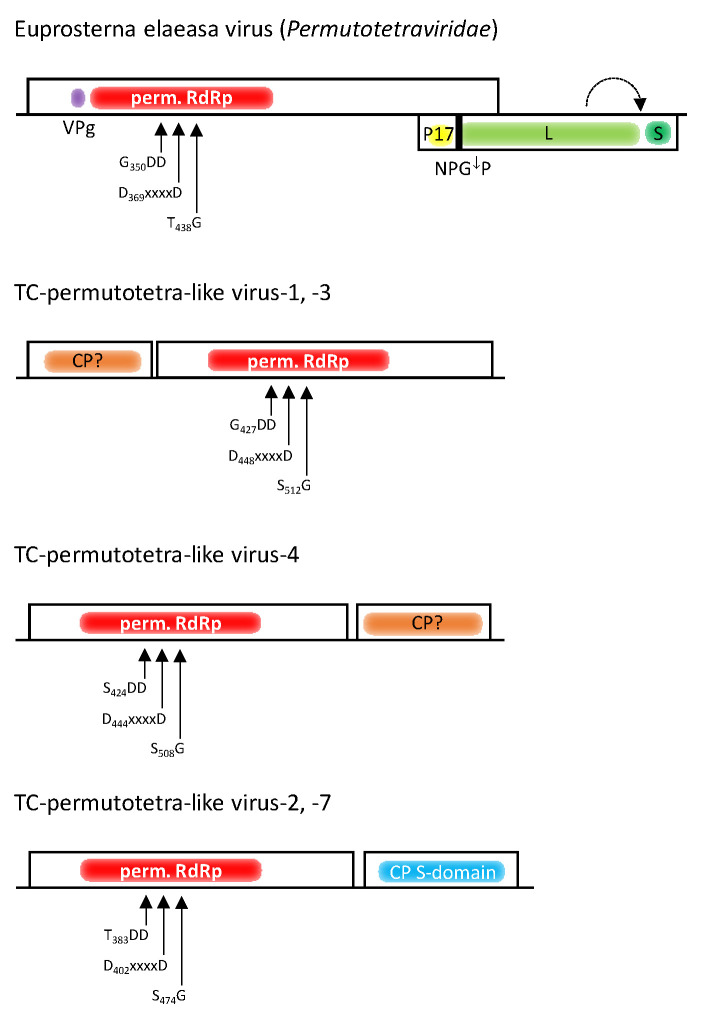
**Genome layout of permutotetraviruses and viruses from the Teltow Canal with permuted RdRp.** Orfs are indicated by boxes. Presented are the RdRp domains with permuted palm motifs (in red) and the CPs (hypothetical CPs in orange; CP with S-domain in blue). Additional protein-encoding gene regions of the Euprosterna elaeasa virus are as follows: VPg (purple), P17 (yellow), L (light green) and S (dark green). L and S are subdomains of the peptidase A21 precursor. NPGP indicates the stop/reinitiation signal, and the dashed arrow indicates the autocatalytic processing site of the peptidase A21 precursor.

DIAMOND had difficulty correctly assigning RdRp sequences to the *Permutotetraviridae* family. It suggested 13 scaffolds, only 2 of which contained a permuted RdRp sequence. Two misassigned scaffolds proved to be hepeli- and astrovirus-like RdRp sequences. The nine remaining sequences contained peptidase A21-like CPs only. With the help of BLAST, an additional five scaffolds representing peptidase A21 sequences plus seven RdRp sequences with a permuted order of palm subdomain motifs were found. Of the 45 clc contigs that were identified by DIAMOND, 5 contigs contained permuted RdRp sequences and 4 contained peptidase A21 sequences. Additional contigs with RdRp and peptidase A21 sequences were detected with BLAST. Permutotetra-like viruses are positive-stranded RNA viruses with icosahedral T = 4 capsids. Their monopartite genome has a length of 5.7 kb with two partly overlapping orfs [58] (Figure 6). The first orf encodes a short polypeptide (VPg) which is covalently attached to the 5′-end of the genome, and an alpha-like RdRp with the conserved palm motifs in the order C–A–B (permuted RdRp) rather than A–B–C (canonical RdRp). The second orf partly overlaps with orf1 and is translated from a subgenomic RNA that encodes a short 17 kDa polypeptide (P17) and a CP precursor with similarity to peptidase A21 (pfam03566). Whereas P17 is released by a cotranslational elongation arrest/re-initiation mechanism at an NPGP sequence, the processed CP is released by autocatalytic cleavage of a short C-terminal oligopeptide from the CP precursor [58].

**Figure 7:**
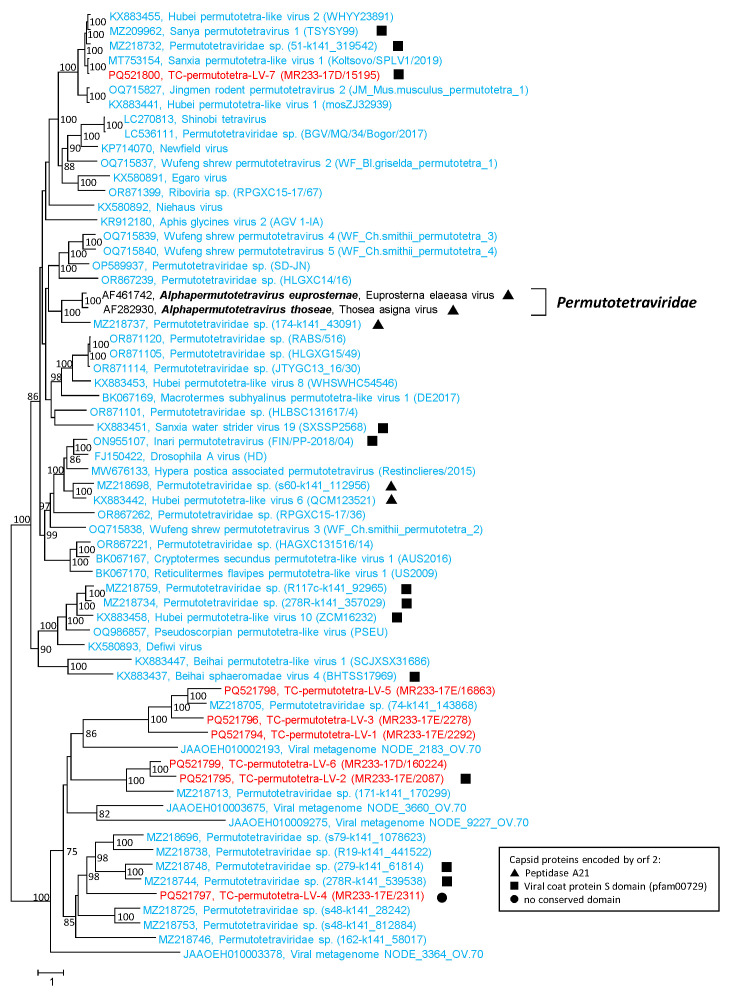
**Phylogenetic analysis of permutotetra-like RdRp.** The RdRp sequences of 58 viruses with permuted palm motifs were aligned with MEGA and used for tree inference with IQ-TREE 2 (optimal substitution model: Q.pfam+F+R6). Two reference strains of the *Permutotetraviridae* family are indicated with a square bracket. Presented are GenBank acc. nos., species names (printed in bold and italics), virus names and strain designations if available (in round brackets). Numbers at nodes indicate bootstrap support greater than 75% obtained after 10,000 ultrafast replications. The bar indicates amino acid substitutions per site. Colour code: red, sequences of viruses from Teltow Canal; blue, unclassified viruses; black, classified reference viruses. Filled triangles (▲) indicate viruses with peptidase A21-like CP, filled squares (■) indicate viruses with CPs with S domain (pfam00729), filled dots (●) indicate viruses with CP without conserved domains.

None of our Teltow Canal permutotetra-like viruses (TC-permutotetra-LVs) exhibited overlapping RdRp and CP sequences. Hence, both genes were analyzed separately. Instead, the TC-permutotetra-LVs exhibited unusual gene layouts: (i) Two viruses had an orf1 encoding a protein without similarity to any protein in GenBank and a second orf which coded for a permuted RdRp (Figure 6). (ii) A third virus possessed an RdRp followed by an ORFan. (iii) Two more viruses had an RdRp-encoding orf1 and a CP with an S domain (pfam00729). The remaining two TC-permutotetra-LVs lacked additional sequences. The seven TC-permutotetra-LV sequences were aligned with RdRp sequences of 2 acknowledged alphapermutotetraviruses and 56 unclassified viruses with permuted RdRp palm motifs. The phylogenetic tree (Figure 7) showed that the TC-permutotetra-LVs clustered into four different branches. Compared to both acknowledged reference viruses, they exhibited highly divergent sequences with similarities lower than 35%. It is worth noting that TC-permutotetra-LV–7 showed 98% similarity to the unclassified Sanxia permutotetra-like virus 1 from the water strider.

**Figure 8:**
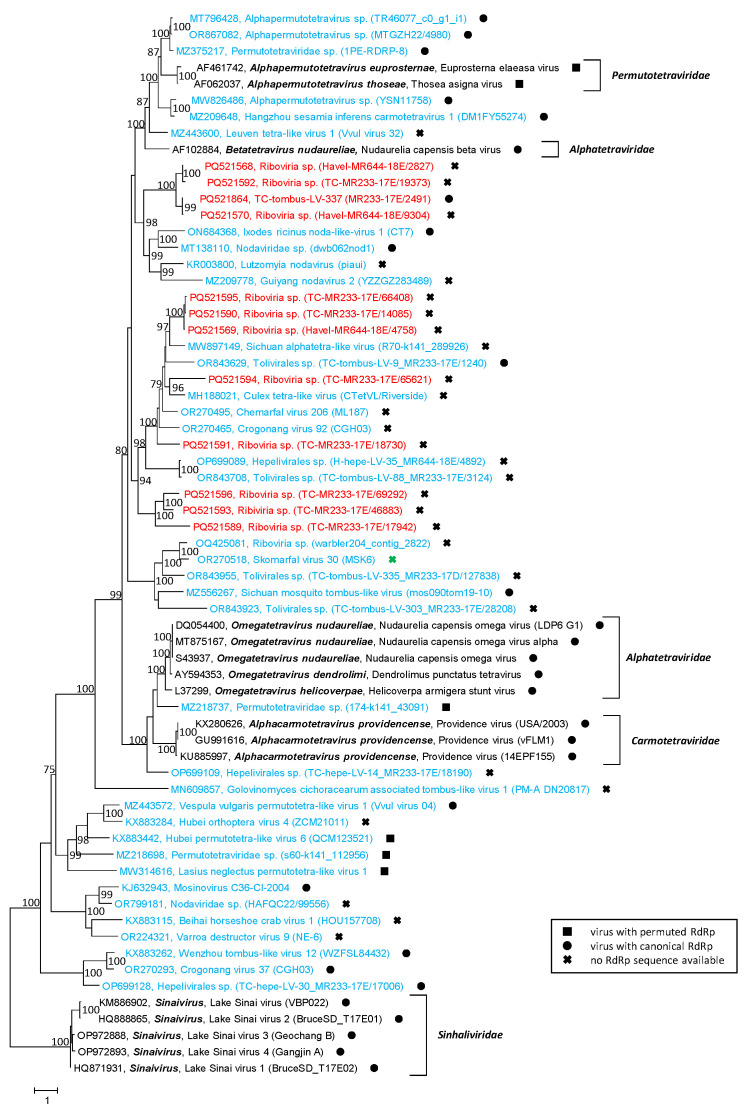
**Phylogenetic analysis of capsid proteins with similarity to peptidase A21.** A total of 65 peptidase A21-like CP sequences were aligned with MEGA and used for tree inference with IQ-TREE 2 (optimal substitution model: Q.pfam+F+R5). Presented are GenBank acc. nos., species names (printed in bold and italics), virus names and strain designations if available (in round brackets). Numbers at nodes indicate bootstrap support greater than 75% obtained after 10,000 ultrafast replications. The bar indicates amino acid substitutions per site. Colour code: red, sequences of viruses from Teltow Canal and Havel River; blue, unclassified viruses; black, classified reference viruses. Filled squares (■) indicate viruses with permuted RdRp palm motifs, filled dots (●) indicate viruses with canonical RdRp, viruses without RdRp sequences are indicated with crosses (**X**).

Analysis of the CP sequences exhibited an inconclusive result. As no dicistronic sequences, but only complete or partial peptidase A21-like sequences, were found, we first searched for the NPGP motif, which is a cis-active translational termination/re-initiation site between the C-terminus of the 17 kDa polypeptide and the N-terminus of the CP precursor. None of our 13 peptidase A21-like sequences had such a sequence motif. Hence, our virus sequences were preliminarily named “*Riboviria* sp.” to express the unsuccessful attempt to assign these sequences to one of the established families. CPs with a peptidase A21 domain are found not only in members of the *Permutotetraviridae* family but also in those of the *Alphatetraviridae*, *Carmotetraviridae* and *Sinhaliviridae* families. Therefore, reference sequences of these virus families, 11 sequences from the Teltow Canal and the Havel River, and 37 sequences of unclassified viruses were aligned and used to infer a phylogenetic tree (Figure 8). As shown in the tree, our viruses clustered into five branches distinct from the reference strains and the unclassified viruses.

### 3.6. Nido-like Viruses

One short deltacoronavirus contig (length: 226 nt) was identified as well as eight highly divergent viruses whose taxonomic rank could be narrowed only to the order *Nidovirales*. The order *Nidovirales* presently contains 14 families with 48 genera and 130 species (https://ictv.global/taxonomy, 10 September 2024). Common to these viruses is an enveloped virion with a helical ribonucleocapsid which consists of a positive-stranded RNA covered with nucleoprotein [59]. The pleomorphic particles are spherical, egg- or rod-shaped with sizes up to 200 nm in length and about 50–70 nm in diameter. The envelope may be studded with large protein spikes (peplomers). The RNA genome of nidoviruses is capped, polycistronic and polyadenylated. The genomes range in size from 13 to 41 kb and exhibit various gene layouts even within a family. The former distinction into small-genome nidoviruses (e.g., members of the suborders *Arnidovirineae* and *Nanidovirineae*) and large-genome nidoviruses (e.g., members of *Cornidoviridae* and *Monidoviridae*) may be obsolete due to many novel viruses with intermediate genome sizes and the strong size variation within families (e.g., viruses of *Medioniviridae*). Nidoviruses exhibit the most complex mechanisms of transcription, translation and polyprotein processing of all RNA viruses. Two to ten orfs encode nonstructural polyproteins as well as a number of structural proteins like spike (S), envelope (E), membrane (M) and nucleocapsid (N) or their homologs and a variant number of accessory proteins. The nonstructural polyproteins undergo autocatalytic processing to yield some 12–16 proteins in those nidoviruses expressing the polyproteins pp1a and pp1ab. A membrane-associated replicase/transcriptase complex facilitates the synthesis of full-length minus-stranded RNA and a ‘nested’ set of subgenomic minus-stranded RNAs that direct the transcription of full-length genomic RNA and subgenomic mRNAs. For nidovirus taxonomy, five hallmark proteins of the pp1AB polyprotein, i.e., 3CLpro (3C-like proteinase), NiRAN (nidovirus RdRp-associated nucleotidyltransferase), RdRp1 (pfam00680, cd23168), ZBD (cys/his-rich Zn-binding domain) and HEL1 (superfamily 1 helicase with P-loop), are generally used to distinguish the members of *Nidovirales*. The host range of nidoviruses includes vertebrates (*Arteriviridae*, *Coronaviridae*, *Tobaniviridae*) and invertebrates (*Roniviridae*, *Mesoniviridae*). Many newer nidoviruses have been characterized from metagenomic sequence data; their hosts remain to be identified.

DIAMOND assigned 11 scaffolds to the *Nidovirales* order; however, 5 of them were clearly misassigned. With the help of BLAST, seven additional nido-like scaffolds were identified. Among these is a very small scaffold (226 nt) that showed 98% amino acid identity to deltacoronaviruses of birds. Another interesting virus is Teltow Canal nido-like virus (TC-nido-LV)–6, which has a genome length of 37 kb and is one of the largest known RNA viruses. The genome of this virus has five orfs. Orf1a has a length of 6933 nt, which corresponds to a protein of 2311 aa. Only one conserved domain was detected, the ASC-1 homology (ASCH) domain (e-value 5.27 × 10^22^). Orf1b protein is fused to orf1a protein by a –1 frameshift at an UUUAAAC RNA signal (nt7045–7051) similar to the orf1ab-encoding region of bunidovirus soil24316 (GenBank acc. no. BK066825). Both 1ab proteins, however, exhibit little similarity. In contrast, the orf2 protein of both viruses share RdRp–ZBD–HEL1 domains (Figure S6).

**Figure 9:**
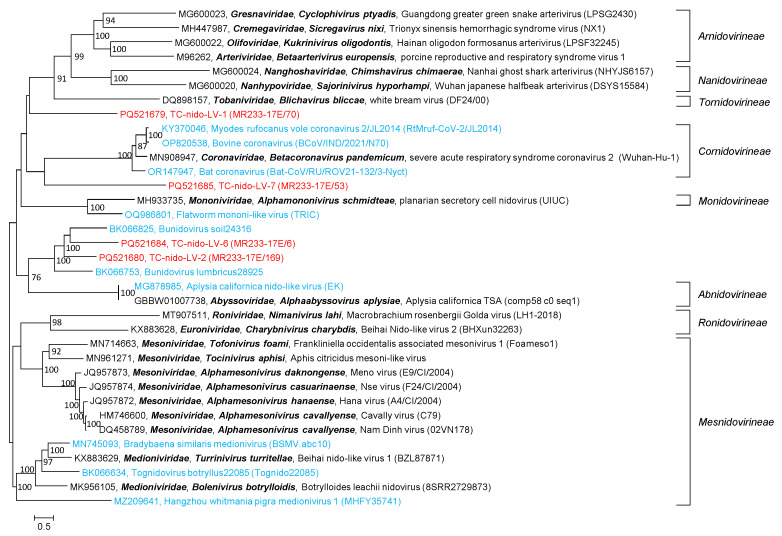
**Phylogenetic analysis of polyprotein 1b of *Nidovirales*.** The polyprotein 1b sequence of 21 classified reference viruses (printed in black) representing the 14 families of the *Nidovirales* order, 4 nido-like viruses of the Teltow Canal (printed in red), and 10 unclassified nido-like viruses (printed in blue) were aligned with MEGA and used for tree inference with IQ-TREE 2 (optimal substitution model: VT+F+R6). Presented are GenBank acc. nos., species names (printed in bold and italics), virus names and strain designations if available (in round brackets). Square brackets indicate suborder names. Numbers at nodes indicate bootstrap support obtained after 10,000 ultrafast replications. The bar indicates amino acid substitutions per site. The tree was arbitrarily rooted with the *Mesnidovirineae* suborder.

Four TC-nido-LVs had polyprotein sequences suitable for a phylogenetic analysis (Figure 9). The tree indicates two TC viruses with similarity to bunidoviruses, which are large unclassified nidoviruses from earthworms and soil samples. Two other viruses, TC-nido-LV–1 and –7, are too diverse for an assignment to be attempted.

### 3.7. Flavivirus Supergroup

Flaviviruses are positive-stranded RNA viruses 40–60 nm in diameter with an enveloped icosahedral core [60] (https://ictv.global/report/chapter/flaviviridae/flaviviridae, accessed on 10 September 2024). The core consists of a single capsid protein (C), and the envelope contains two or more glycoproteins, depending on the genus. Where the virion structure was resolved by cryo-electron microscopy, the data demonstrate 90 dimers of the envelope protein E or its homolog being arranged into an icosahedral scaffold but lacking a T = 3 quasi-equivalent environment (e.g., [61]). The genomic RNA has a length of 9–13 kb and encodes a single polyprotein that is processed by viral and cellular proteases to yield three or more structural proteins and, in most viruses, seven nonstructural proteins. Both translation initiation by cap-dependent (genus *Orthoflavivirus*) and cap-independent mechanisms (genera *Hepacivirus*, *Pegivirus*, *Pestivirus*) have been described. The flavivirus supergroup comprises the *Flaviviridae* family with presently four genera and 97 species plus many novel viruses awaiting classification [60,62]. Among the unclassified viruses with sequence similarity to *Flaviviridae* are diatom colony-associated ssRNA virus 1 and Jῑngmén viruses [63,64]. Special features of Jῑngmén viruses are their segmented genome and their size of 60–80 nm in diameter. Two of the four segments exhibit sequence similarity to the flavivirus NS5 gene (segment 1) and NS3 gene (segment 3), respectively, whereas three to four unique proteins without similarity to known proteins are encoded by the remaining segments. Viruses of all four flavivirus genera infect mammals; only members of the genus *Orthoflavivirus* use arthropods (insects, ticks) as vectors.

**Figure 10:**
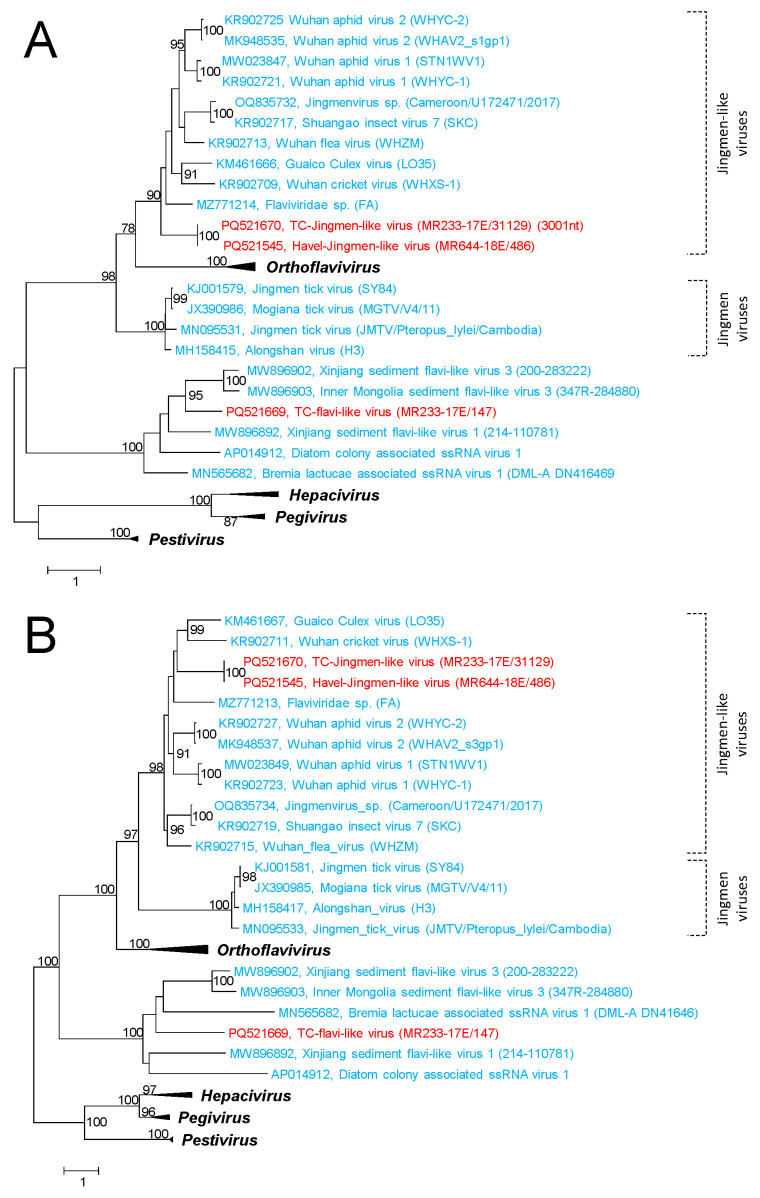
***Flaviviridae* and Jῑngmén-like viruses.** A total of 36 sequences of flavivirus NS5- (**A**) and NS2-NS3-like proteins (**B**) were aligned with MEGA and used for tree inference with IQ-TREE 2; optimal substitution model: Q.pfam+F+I+G4 (**A**) and LG+F+R5 (**B**). Presented are GenBank acc. nos., species names (printed in bold and italics), virus names and strain designations if available (in round brackets). Square brackets indicate viruses of the Jῑngmén virus group and Jῑngmén-like viruses. Numbers at nodes indicate bootstrap support greater than 75% obtained after 10,000 ultrafast replications. The bar indicates amino acid substitutions per site. Colour code: red, sequences of viruses from Teltow Canal and Havel River; blue, unclassified viruses; black, classified reference viruses. Details of the phylogenetic trees are presented in Figure S7A,B.

We identified three viruses with sequence similarity to flaviviruses and Jῑngmén viruses in the Teltow Canal and the Havel River. The sequence of TC-flavi-like virus has a length of 12,630 nt and encodes a polyprotein of 4155 aa. This virus has similarity to several unclassified flavi-like viruses, e.g., viruses detected in sediment samples in China (MW896892, MW896903, MW806903) and viruses associated with diatoms (AP014912) and oomycetes which infect lettuce (MN565682) (for reference, see [17,64]). The Jῑngmén-like viruses from the Teltow Canal and the Havel River have similarity to a subgroup of Jῑngmén viruses. Viruses of this subgroup have an RNA segment 2 with two non-overlapping orfs, the first of which encodes the hypothetical viral protein VP4 [61]. A phylogenetic analysis of the RdRp sequences included (i) our viruses from the Teltow Canal and the Havel River, (ii) 20 unclassified viruses from other metagenomics studies and (iii) 13 reference strains representing the four genera of the *Flaviviridae* family. The phylotree presented six major clades. Three branches correspond to the four genera of the *Flaviviridae* family, a fourth cluster contains unclassified flavi-like viruses including our TC-flavi-like virus, and two clades comprise the Jῑngmén viruses (Figure 10A and Figure S7A). It is worth noting that the Jῑngmén tick virus and three related viruses with only one orf of RNA segment 2 cluster together and the 12 Jῑngmén-like viruses with two orfs of segment 2 form a second clade. In order to verify the Jῑngmén virus subgroups, a second phylogenetic analysis was conducted. For this, segment 3 of the Jῑngmén viruses and the corresponding NS2-NS3 region of flaviviruses and flavi-like viruses were investigated. The phylogenetic tree (Figure 10B and Figure S7B) confirmed the split observed in the RdRp tree.

### 3.8. Nege-like Virus

**Figure 11:**
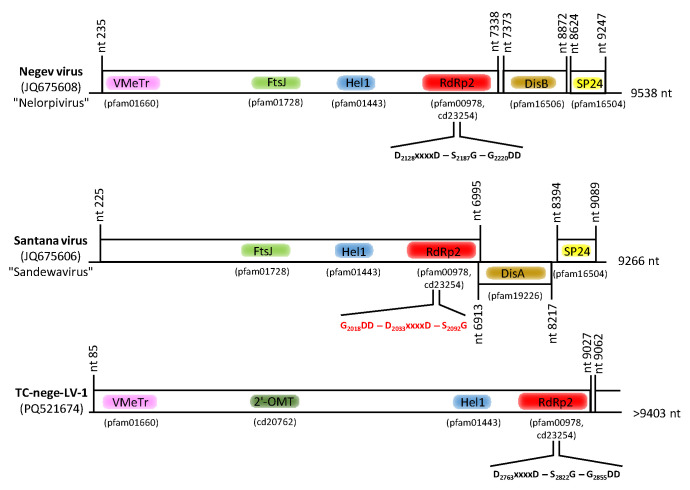
Gene layout of Negev virus (“Nelorpivirus” group), Santana virus (“Sandewavirus” group) and Teltow Canal nege-like virus 1 (TC-nege-LV-1). Orfs are indicated by boxes with approximate positions of conserved protein domains. Canonical and permuted order of conserved RdRp palm motifs are indicated. Abbreviations: DisA, viral DisA glycoprotein; DisB, viral DisB glycoprotein; FtsJ, SAM-dependent FtsJ-like (nucleoside-2′-O-)-methyltransferase (capping enzyme); Hel1, viral superfamily 1 RNA helicase; SP24, 24 kDa virion membrane protein of plant and insect viruses; RdRp2, type 2 RNA-dependent RNA polymerase; VMeTr, viral SAM-dependent G-7-methyltransferase.

Negev virus and a few other related viruses from insects have been described as enveloped (ether-sensitive), spherical particles 45–55 nm in diameter with monopartite, single-stranded RNA genomes [65]. The polyadenylated RNA has a length of 9–10 kb with three orfs. Orf 1 encodes a nonstructural polyprotein with four conserved protein domains (Figure 11), i.e., a viral G-7-methyltransferase (pfam01660), a ribosomal RNA 2′-O-methyltransferase FtsJ-like domain (pfam01728), a type 1 helicase (pfam01443) and an alpha-like type 2 RdRp (pfam00978, cd23254). The second orf codes for a putative glycoprotein and the third orf for a putative membrane protein of the SP24 superfamily with transmembrane regions (pfam16504). Common to Negev viruses and related viruses is their “insect only” specificity: all available virus strains were isolated from insects (various mosquito species, sandflies, dung flies, aphids, mealybugs, seed bugs) and propagate well in insect cells but not in mammalian cell lines. A new taxon “Negevirus” with two subgroups, “Nelorpivirus” and “Sandewavirus”, has been proposed to accommodate these viruses [66]. Meanwhile, more than 350 complete or partial nege-like virus sequences have been released by GenBank (as of 10 September 2024).

DIAMOND failed to assign sequences of the Teltow Canal dataset to nege-like viruses, but with BLAST, five scaffolds were identified. Each pair of sequences corresponded to RdRp and helicase genes. One scaffold of 9.4 kb contained a complete nege-like orf1 and a partial orf2 sequence and was named Teltow Canal nege-like virus (TC-nege-LV)–1. The helicase–RdRp sequence of this virus was aligned with that of 27 nelorpiviruses, 14 sandewaviruses, 14 kitaviruses of the *Blunervirus*, *Cilevirus* and *Higrevirus* genera, and two idaeoviruses. Though TC-nege-LV–1 showed a comparable gene layout (Figure 11), the alignment revealed two specific features: (i) a variant methyltransferase gene which exhibited similarity to the nucleoside-2′-O-methyltransferase of nidoviruses but not to the FtsJ domain, and (ii) an insertion of circa 1000 aa located 5′ to the helicase gene. It is worth noting that the RdRp sequences of the “Sandewavirus” clade exhibited a permuted order of the palm subdomain motifs (canonical order: A–B–C; permuted order: C–A–B; compared in Figure 11). The phylogenetic analysis confirmed the divergent nature of TC-nege-LV–1; it clustered at the root of the sandewaviruses but with a long branch (Figure S8).

### 3.9. Rhabdoviridae

Rhabdoviruses are negative-stranded RNA viruses [67,68]. The virions of most members are enveloped, bullet-shaped or bacilliform; particle sizes range from 100 to 460 nm in length and 45 to 100 nm in diameter. The genomic RNA is 10–16 kb in length and encodes five canonical structural proteins plus a variable number of accessory proteins. The genomic RNA is covered with nucleoprotein (N) to form a helical ribonucleoprotein (RNP) complex. The RNP is associated with phosphoprotein (P) and a large polymerase (L). The envelope is studded with glycoprotein (G) which forms trimeric peplomers and shimmed with matrix protein (M). As an exception, few rhabdoviruses may have a non-enveloped filamentous virion or a bi-segmented genome. Each protein is translated from subgenomic mRNAs. For this, transcription follows a “stop-start” mechanism leading to a 3′–5′ gradient of mRNA synthesis. Replication is directed from a full-length anti-genome RNA intermediate. Rhabdoviruses exhibit a wide host range including vertebrates, invertebrates and plants. Many rhabdoviruses use arthropods as vectors. The *Rhabdoviridae* family is large and comprises four subfamilies, 56 genera and 434 species [69]. In addition, more than 300 rhabdoviruses await classification.

DIAMOND assigned one scaffold and one contig greater than 1 kb to the *Rhabdoviridae* family. The scaffold had a length of 10,074 nt, represented an almost complete genome of a novel virus and was named TC-rhabdo-like virus. Its divergent sequence clusters with members of *Deltarhabdovirinae*, and Hubei rhabdo-like virus 2 from nematodes (subclass Spirurina) and Plasmopara viticola lesion associated mononega virus 1 from an oomycete are its closest relatives (Figure S9). The sequence divergence suggests a new rhabdovirus genus.

### 3.10. Chuviridae

The first chǔviruses were described by Li et al. [70] as negative-stranded RNA viruses. Based on their RdRp sequences, chǔviruses were accommodated in a new virus family, *Chuviridae*, of the order *Jingchuvirales*. Meanwhile, this family comprises 16 genera and 43 species (https://ictv.global/taxonomy; accessed 10 October 2024), and some 750 chu-like viruses await classification. Chǔviruses are still uncultured; available genomic sequences exhibit segmented or unsegmented, linear or circular genomes of 9–12 kb with 2 to 4 orfs and various gene layouts [71]. Details of virion structure and viral life cycle are unknown. Most chǔviruses were detected in various arthropods (insects, ticks, spiders, crustaceans), but some were associated with insectivorous bats, fishes, reptiles, nematodes and eggplants.

DIAMOND assigned one scaffold and one contig greater than 6 kb to the *Chuviridae* family. The corresponding sequences represented an almost complete RdRp sequence of a divergent virus, named TC-chu-like virus. As only an RdRp sequence is available, structural features of its genome remain unclear (e.g., segmented or unsegmented, linear or circular RNA genome). Figure S9 presents a phylogenetic analysis of TC-chu-like virus RdRp aligned with the 10 closest hits of the BLAST search plus reference sequences of related virus families. The data suggest a novel genus of the family *Chuviridae*.

## 4. Discussion

The advent of high-throughput sequencing techniques has led to the identification of a plethora of novel viruses including those of invertebrates. It has been estimated that the number of UViG sequences exceeded 750,000 in 2018 [34]. Viruses are obligate cellular parasites and depend on permissive host cells. Whereas many cultured viruses induce visible cytopathic effects on infected cells, most of the UViG sequences lack reliable information on hosts and infection sequelae—though environmental viruses are present in abundance. In addition, many UViGs were detected in tissues, intestinal contents or faecal samples. Albeit linked to a potential host, uncertainties exist regarding whether the unveiling of novel viruses in such samples really indicates infection. This conundrum is a drop of bitterness that mars the many merits of unbiased virus sequencing. However, *“virologists, especially viral taxonomists, have no choice but to work within this new reality”* [37].

In the present study, we searched our scaffold/contig banks obtained from two environmental samples from the Teltow Canal and the Havel River for sequences of virus families known to include invertebrate viruses, i.e., for *Birnaviridae*, *Flaviviridae*, *Nodaviridae*, *Permutotetraviridae*, *Rhabdoviridae*, and the many families of the *Nidovirales*, *Reovirales* and *Bunyaviricetes*, as well as for viruses of the unclassified Jῑngmén virus and Negev virus groups. Viruses of *Picornavirales* and *Hepelivirales* were excluded as we have already screened our scaffold/contig banks for these viruses [21,22,41]. As a result, here we present complete or partial sequences of more than 300 virus strains, roughly only 11% of which could be classified at the genus level. The remaining viruses were novel, and many exhibited unexpected features.

**Noda-like viruses:** Between 400 and 500 sequences were assigned by DIAMOND to the *Nodaviridae* family but many of these were rather short, and no attempts were made to verify them by BLAST. Of the 107 sequences with noda-like RdRp, only 15 sequences were assigned to the genus *Alphanodavirus* on basis of their phylogenetic clustering and protein B2 sequences. Another 15 viruses were identified as candidates of *Betanodavirus* judging from their RdRp sequences (n = 10) and characteristic CP sequences (n = 5). The remaining viruses with noda-like RdRp had either partial sequences unsuited for assignment or showed significant differences, e.g., dicistronic RNAs comprising noda-like RdRp and CP domains or ORFans.

**Reo-like viruses:** The reo-like sequences of the Teltow Canal sample were dominated by rotavirus A, whereas no rotavirus was detected in the Havel River sample. This is a plausible finding as the Teltow Canal receives increased discharge of a municipal wastewater treatment plant in the summer months in order to relieve the Havel River, which is used for recreational purposes. Consistently, we have previously reported the presence of other viruses in the Teltow Canal known to indicate faecal contamination, e.g., certain plant viruses, posaviruses and Aichi virus [22,40]. In addition, segments of a novel seadornavirus plus highly diverse RdRp sequences of novel sedoreo- and spinareoviruses were detected (Figure 3). These and the many other unclassified virus sequences of both virus families indicate that the *Reovirales* order comprises far more than 97 members.

**Bunya-like viruses:** The many members of the *Bunyaviricetes* class are presently classified on the basis of pairwise evolutionary distance (PED) values obtained in DEmARC analyses (see 2016.030a-vM.A.v6.Bunyavirales.pdf downloaded at https://ictv.global/taxonomy, accessed 10 September 2024). Moreover, hundreds of bunya-like viruses are still unclassified. As shown in Figure 4, 18 of the 31 bunya-like viruses of the Teltow Canal and the Havel River with lengths up to 12.4 kb were suited for phylogenetic analysis of their RdRp, but none clustered with any of the acknowledged families.

**Birna-like viruses:** Our Teltow Canal dataset also indicates an unexplored and diverse birnavirus virome. None of the Teltow Canal birna-like viruses clustered with members of the known birnavirus genera.

**Permutotetra-like viruses:** A permuted order of the conserved RdRp palm motifs has been described for birnaviruses and permutotetraviruses which cluster in phylogenetic analyses with the members of the phylum *Pisuviricota* [56,72,73]. Permuted RdRps were also found in a few hepe-like viruses, nege-like viruses (see below) and some other viruses of the *Kitrinoviricota* phylum [41,73]. Here, we identified seven viruses with permutotetra-like RdRp but with three gene layouts which differ from those of permutotetraviruses (Figure 6). None of our peptidase A21 sequences had the NPGP motif used to release the N-terminal P17 peptide characteristic of permutotetraviruses.

**Nido-like viruses:** We detected one short deltacoronavirus-specific scaffold. The main reservoir of deltacoronaviruses is birds, especially waterfowl, but transmission to pigs and other mammals occurs frequently [74,75]. The presence of a deltacoronavirus in the Teltow Canal is consistent with the many ducks, coots and swans living there. The remaining nido-like viruses were highly diverse, which impeded their assignment to families. Three viruses were bunidovirus-like of which MR233-17E/6 had an (almost) complete genome of 37 kb—only planarian secretory cell nidovirus and bunidovirus soil24316 have longer RNA genomes [76].

**Flavivirus supergroup:** The TC-flavi-like virus shares characteristic domains with the acknowledged flavivirus reference strains but only with little similarity. The NS3 sequences exhibit similarity to a trypsin-like peptidase and a superfamily II helicase. The RdRp domain with similarity to the RdRp subgroup cd01699 is separated from the NS3-like domain by a stretch of more than 2000 amino acids without conserved protein domains. This gene layout and sequence similarity are shared by five similar viruses (Figure 10A,B). The host ranges of these viruses are unclear. Three of these viruses were detected in lake/pond/river sediments, which is consistent with our environmental sample. A fourth virus was associated with a diatom colony in a tidal pool, and the fifth virus was identified in an oomycete metagenome. The available sequences of all six viruses of this clade, which range in size from 11.4 to 15.7 kb, obviously lack structural proteins. It is unknown whether this indicates endogenous viruses (which do not need CPs) of a fungal-like or protist host, or is the result of partial sequencing.

Two other flavi-like viruses are related to the Jῑngmén virus group. In our alignments, the Jῑngmén viruses from ticks and the Jῑngmén-like viruses from insects constitute two distinct clades (Figure 10A,B). Viruses of both clades also differ in their gene layouts. The assumption of an arthropod host of our TC- and Havel-Jῑngmén-like viruses is consistent with the known host range of other Jῑngmén viruses.

**Nege-like viruses:** The presently available sequences of nege-like viruses suggest two clades of “insect only” viruses related to members of *Kitaviridae* and *Mayoviridae* (Figure S8). Proposed names of these clades are “Nelorpivirus” and “Sandewavirus” [66]. Besides sequence divergence, we noticed two additional features that justify the creation of two genera. First, both virus groups differ in genome layouts (Figure 11). Second, the order of the conserved RdRp palm domains differs in both virus groups: nelorpiviruses have a canonical RdRp, while sandewaviruses possess a permuted polymerase. Though only a partial TC-nege-LV–1 sequence was available, the phylogenetic tree (Figure S8) reveals a third branch of nege-like viruses. TC-nege-LV–1 has a canonical RdRp but a long insertion of about 1000 amino acids between the 2′-O-methyltransferase and the helicase domain (Figure 11).

**Rhabdo-like virus:** Our TC-rhabdo-like virus sequence clusters with members of *Deltarhabdovirinae*. All viruses of this subfamily use arthropod hosts. However, two related viruses, the unclassified Plasmopara viticola lesion associated mononega virus 1 and the Hubei rhabdo-like virus 2, were detected in a fungus and a nematode, respectively, which may raise doubt regarding an arthropod host of TC-rhabdo-like virus.

**Chu-like virus:** The presently known chǔviruses are UViGs, and the *Chuviridae* taxonomy is based essentially on RdRp similarity. Other characteristic features like the number of genome segments, gene layout, replication mechanism, host range and pathogenicity are of secondary importance.

**Genetic exchange among RNA viruses:** Capsid proteins with a jellyroll fold are the building blocks of many icosahedral T = 3 and T = 4 capsids. The invertebrate viruses investigated in this study make use of five CP types, i.e., the viral coat protein S-domain (pfam00729), the viral coat protein VNN (pfam11729), the luteovirus coat protein (pfam00894), the peptidase A6-like CP (pfam01829) and the peptidase A21-like CP (pfam03566). These five CPs may be combined with the RdRps of various supergroups, indicating horizontal gene transfer in the evolution of these viruses. Table 1 presents a few conspicuous examples from the Teltow Canal, the Havel River and other sources. In addition, many virus sequences presented orfs that encode putative structural proteins without conserved domains (see Figure S1: sequences marked with **✕**). Such viruses are not shown in Table 1. It remains to be investigated whether highly divergent sequences or novel domain structures prevented perception of the details of these ORFans. Koonin et al. [77] and Dolja and Koonin [78] developed hypotheses that explain the evolution of eukaryotic viruses by the *“mixing and matching of gene modules”* in the *“crucible of eukaryogenesis”* [78]. It appears likely that similar gene shuffling mechanisms may still contribute to the occasional emergence of viruses with unusual genotypes.

## 5. Conclusions

Invertebrates exert important functions in freshwater ecosystems but are endangered by anthropogenic activities, pollution and climate change. Viruses of many families are known to infect invertebrates, using them either as vectors or as genuine hosts. Notwithstanding its role as an essential component of the virosphere, the diversity of invertebrate viruses is less well investigated compared to that of their vertebrate counterparts, and myriads of invertebrate viruses may still be undiscovered. Analysis of the viromes of two rivers in Berlin revealed hundreds of novel viruses assumed to infect invertebrates. These viruses present highly divergent genomes, numerous ORFans, and gene layouts indicating extensive horizontal gene transfer among environmental viruses. The lack of knowledge of their genuine hosts calls for intensified research, at least for the most interesting virus groups.

## Figures and Tables

**Table 1:** CP/RdRp combinations suggesting gene exchange.

Capsid Protein	RdRp Superfamily	Examples
Viral coat protein S-domain (pfam00729)	RdRp1 (pfam00729)	Plasmopara halstedii virus A, Sclerophthora macrospora virus A, Beijing sediment noda-like virus1, Beihai noda-like virus 5, Ripothoz virus TC-noda-LV–11, –16
	RdRp3 (pfam00998)	*Tombusviridae* (except *Luteovirus*), Tombunodavirus
	RdRp4 (pfam02123)	*Sobemovirus*, *Polemovirus*
	permuted RdRp	Inari permutotetravirus, Beihai sphaeromadae virus 4, TC-permutotetra-LV–2, –7
Nodavirus capsid protein VNN (pfam11729)	RdRp1 (pfam00680)	*Betanodavirus*, Orsay nodavirus, Le Blanc nodavirus, Santeuil nodavirus
	RdRp3 (pfam00998)	Wufeng shrew carmotetravirus 1
Luteovirus coat protein(pfam00894)	RdRp1 (pfam00729)	Craigies Hill virus
	RdRp3 (pfam00998)	*Luteovirus*
	RdRp4 (pfam02123)	*Enamovirus*, *Polerovirus*
Peptidase A6 (pfam01829)	RdRp1 (pfam00680)	*Alphanodavirus*, Ripothip virus, TC-noda-LV–92
	RdRp2 (pfam00978)	H-hepe-LV–5, TC-hepe-LV–20
	RdRp3 (pfam00998)	TC-tombus-LV–336, –339, –340, H-tombus-LV–4, –6, –15, Shahe isopoda virus 5
	RdRp4 (pfam02123)	H-solemo-LV–4, –6, –10, –15, TC-solemo-LV–11
	permuted RdRp	TC-hepe-LV–22, –24
Peptidase A21 (pfam03566)	RdRp1 (pfam00680)	*Sinhaliviridae*, Lutzomyia nodavirus
	RdRp2 (pfam00978)	*Alphatetraviridae*, Hubei hepe-like virus 2, TC-hepe-LV–30
	RdRp3 (pfam00998)	*Carmotetraviridae*, TC-tombus-LV–87, –337, Sichuan mosquito tombus-like virus
	permuted RdRp	*Permutotetraviridae*, *Permutotetraviridae* sp. 174-k141_43091, Hubei permutotetra-like virus 6

## Data Availability

BioProject ID: PRJNA1174387; Biosamples: SAMN44339963, SAMN44339964; Short Read Archive: SRR31035838, SRR31035839; GenBank accession numbers: PQ521543–PQ521867.

## Supplementary Materials

The following supporting information can be downloaded at https://www.mdpi.com/article/10.3390/microorganisms12112361/s1, Figure S1: Phylogenetic analysis of the RdRp of noda-like viruses; Figure S2: Phylogenetic analysis of structural proteins with jellyroll fold; Figure S3: Phylogenetic analysis of reo-like RdRp sequences; Figure S4: Phylogenetic analysis of bunyavirus RdRp sequences; Figure S5: Phylogenetic analysis of the birnavirus proteins; Figure S6: Genome layout of bunidoviruses and TC-nido-LVs; Figure S7: Phylogenetic analysis of flavivirus-like proteins; Figure S8: Phylogenetic analysis of helicase–RdRp sequences of nege-like viruses and related viruses of the Kitaviridae and Mayoviridae families; Figure S9: Phylogenetic analysis of the polymerase of the subphylum Haploviricotina; Table S1: Compilation of Teltow Canal and Havel River viruses.
