# Supplementary material for: Metagenomic Analyses of Water Samples of Two Urban Freshwaters in Berlin, Germany, Reveal New Highly Diverse Invertebrate Viruses
Source: Microorganisms. 2024 Nov 19;12(11):2361. doi: 10.3390/microorganisms12112361 (PMC11596407; doi:10.3390/microorganisms12112361)
Supplement: Supplementary file 1 [file microorganisms-12-02361-s001.zip › microorganisms-3298242-supplementary.pdf]

Supplementary material to

Metagenomic analyses of water samples of two urban freshwaters in Berlin, Germany, reveal new highly diverse invertebrate viruses

Roland Zell[1]*, Marco Groth[2], Lukas Selinka[1], and Hans-Christoph Selinka[3]

[1] Section of Experimental Virology, Institute for Medical Microbiology, Jena University Hospital, Friedrich Schiller University, Jena, Germany
[2] CF Next Generation Sequencing, Leibniz Institute on Aging, Fritz Lipmann Institute, Jena, Germany
[3] Section II 1.4 Microbiological Risks, Department of Environmental Hygiene, German Environment Agency, Berlin, Germany

Legends to Supplementary Figures:

Figure S1: Phylogenetic analysis of the RdRp of noda-like viruses. The RdRp sequences of 83 Teltow Canal noda-LVs (printed in red), 16 Havel noda-LVs (printed in red), 51 classified reference strains (printed in black) of the *Nodaviridae*, *Sinhaliviridae*, *Carmotetraviridae*, *Solemoviridae* and *Tombusviridae*, and 88 unclassified viruses (printed in blue) were aligned with MEGA and used for maximum likelihood tree inference with IQ-TREE 2 (optimal substitution model: Q.pfam+F+R8). Presented are GenBank acc. nos., species names (printed in bold and italics), virus names and strain designations if available (in round brackets). Square brackets indicate subfamily, family and order names. Information on structural proteins is given if available: ■, peptidase A6 (pfam01829), ◘, peptidase A21 (pfam03566), ▲, betanodavirus capsid protein VNN (pfam11729), **O**, viral coat protein with S-domain (pfam00729), ●, luteovirus coat protein, ✖, hypothetical protein without conserved domains. Numbers at nodes indicate bootstrap support greater than 75% obtained after 10,000 ultrafast replications. The tree was arbitrarily rooted with solemovirus sequences. The bar indicates amino acid substitutions per site.

Figure S2: Phylogenetic analysis of structural proteins with jellyroll fold. A total of 150 sequences representing the protein superfamilies pfam00894, pfam00729 and pfam11729 were aligned with MEGA and used for maximum likelihood tree inference with IQ-TREE 2 (optimal substitution model: Q.pfam+F+R5). Included are 24 noda-like viruses of Teltow Canal and Havel River (printed in red), 79 classified reference viruses (printed in black) and 47 unclassified viruses (printed in blue). Presented are GenBank acc. nos., species names (printed in bold and italics), virus names and strain designations if available (in round brackets). Thin square brackets indicate tombusvirus subfamilies, whereas thick square brackets indicate protein superfamilies. Numbers at nodes indicate bootstrap support greater than 75% obtained after 10,000 ultrafast replications. The tree was arbitrarily rooted with betanodavirus capsid protein sequences. The bar indicates amino acid substitutions per site.

Figure S3: Phylogenetic analysis of reo-like RdRp sequences. The RdRp sequences of 52 classified reference viruses (printed in black), 17 reo-like viruses from the Teltow Canal (TC-reo-LVs, printed in red) and 27 unclassified viruses (printed in blue) were aligned with MEGA and used for tree inference with IQ-TREE 2 (optimal substitution model: VT+F+R8). Presented are GenBank acc. nos., species names (printed in bold and italics), virus names and strain designations if available (in round brackets). Square brackets indicate families. Numbers at nodes indicate bootstrap support greater than 75% obtained after 10,000 ultrafast replications. The bar indicates amino acid substitutions per site.

Figure S4: Phylogenetic analysis of bunyavirus RdRp sequences. The RdRp sequences of 40 classified reference viruses (printed in black) representing the 15 families of the *Bunyaviricetes* class, 18 bunya-like sequences of Teltow Canal and Havel River (printed in red) and 34 unclassified bunya-like sequences (printed in blue) were aligned with MEGA and used for tree inference with IQ-TREE 2 (optimal substitution model: VT+F+R8). Presented are GenBank acc. nos., species names (printed in bold and italics), virus names and strain designations if available (in round brackets). Square brackets indicate families and orders. Numbers at nodes indicate bootstrap support greater than 75% obtained after 10,000 ultrafast replications. The bar indicates amino acid substitutions per site. Note: members of the *Arenaviridae* and *Mypoviridae* families cluster together.

Figure S5: Phylogenetic analysis of the birnavirus proteins. (A) Analysis of VP1 (RdRp). The VP1 sequences of 25 classified reference viruses (printed in black) representing the 7 genera of the

Birnaviridae family, 11 birna-like viruses of the Teltow Canal (printed in red) and 22 unclassified bunya-like viruses (printed in blue) were aligned with MEGA and used for tree inference with IQ-TREE 2 (optimal substitution model: LG+F+R6). Presented are GenBank acc. nos., species names (printed in bold and italics), virus names and strain designations if available (in round brackets). Square brackets indicate genus names. Numbers at nodes indicate bootstrap support greater than 75% obtained after 10,000 ultrafast replications. The bar indicates amino acid substitutions per site. The tree was arbitrarily rooted with sequences of chicken proventricular necrosis virus. **(B)** Analysis of VP2 (CP). The VP2 sequences of 18 classified reference viruses (printed in black) representing the 7 genera of the *Birnaviridae* family, 11 birna-like viruses of the Teltow Canal (printed in red) and 11 unclassified bunya-like viruses (printed in blue) were aligned with MEGA and used for tree inference with IQ-TREE 2 (optimal substitution model: Q.pfam+F+R4). Presented are GenBank acc. nos., species names (printed in bold and italics), virus names and strain designations if available (in round brackets). Square brackets indicate genus names. Numbers at nodes indicate bootstrap support greater than 65% obtained after 1000 replications. The bar indicates amino acid substitutions per site. This is a midpoint-rooted tree.

Figure S6: Genome layout of bunidoviruses and TC-nido-LVs. Orfs are indicated by boxes. Locations of conserved protein domains as suggested by the Conserved Domain Database (CDD) search tool are indicated and highlighted in different colours. Abbreviations: –1FS, ribosomal frameshift site; ASCH, ASC-1 homology domain (cd06555, COG4043); ExoN, 3'–5'-proofreading exonuclease (cd21528, COG0847); HEL1, superfamily 1 P-loop helicase (pfam13087); MeTr, S-adenosyl-dependent methyltransferase (pfam05063, pfam13649, pfam13847); NADAR, NAD and ADP-ribose domain of unknown function (pfam08719); Prot, trypsin-like serine protease (pfam00089, cd00190); RdRp1, type 1 RNA-dependent RNA polymerase (pfam00680); ZBD, Cys-His-rich zinc-binding domain (cd21343, cd21399, cd21403).

Figure S7: Phylogenetic analysis of flavivirus-like proteins. The NS5-like protein sequence (RdRp) **(A)** and the NS2B-NS3-like protein sequence (protease/helicase) **(B)** of 13 classified reference viruses (printed in black) representing the 4 genera of the *Flaviviridae* family, 1 flavi-like virus and 2 Jīngmén-like viruses of Teltow Canal and Havel River (printed in red), and 20 unclassified flavi-like and Jīngmén-like viruses (printed in blue) were aligned with MEGA and used for tree inference with IQ-TREE 2 (optimal substitution model: Q.pfam+F+I+G4). Presented are GenBank acc. nos., species names (printed in bold and italics), virus names and strain designations if available (in round brackets). Square brackets indicate genus names. Dashed square brackets indicate two clades of Jīngmén viruses and Jīngmén-like viruses. Numbers at nodes indicate bootstrap support obtained after 1000 replications. The bar indicates amino acid substitutions per site. This is a midpoint-rooted tree.

Figure S8: Phylogenetic analysis of helicase–RdRp sequences of nege-like viruses and related viruses of the *Kitaviridae* and *Mayoviridae* families. The helicase–RdRp sequences of 15 classified reference viruses (printed in black) representing the *Kitaviridae* and *Mayoviridae* families, one nege-like virus of the Teltow Canal (printed in red), and 45 sequences of the unclassified Negev virus and nege-like viruses (printed in blue) were aligned with MEGA and used for tree inference with IQ-TREE 2 (optimal substitution model: Q.pfam+F+R5). Presented are GenBank acc. nos., species names (printed in bold and italics), virus names and strain designations if available (in round brackets). Square brackets indicate family/order names, dashed square brackets indicate the proposed names of some unclassified viruses. Numbers at nodes indicate bootstrap support obtained after 10,000 ultrafast replications. The bar indicates amino acid substitutions per site. The tree was arbitrarily rooted with *Mayoviridae* sequences.

Figure S9: Phylogenetic analysis of the polymerase of the subphylum *Haploviricotina*. The RNA-dependent RNA polymerase sequence of 84 classified reference viruses (printed in black) representing 47 genera of the *Rhabdoviridae* family, 16 genera of the *Chuviridae* family, and the remaining 17 virus families of the *Haploviricotina* subphylum, plus 1 rhabdo-like and chu-like virus each of the Teltow Canal (printed in red), 3 unclassified rhabdo-like viruses (printed in blue) and 4 unclassified chu-like viruses (printed in blue) were aligned with MEGA and used for tree inference with IQ-TREE 2 (optimal substitution model: Q.pfam+T+R9). Presented are GenBank acc. nos., species names (printed in bold and italics), virus names and strain designations if available (in round brackets). Square brackets indicate higher rank names (subfamily, family, order, subphylum). Numbers at nodes indicate bootstrap support obtained after 10,000 ultrafast replications. The bar indicates amino acid substitutions per site. The tree was arbitrarily rooted with sequences of the genus *Yuyuevirus*.

Figure S1

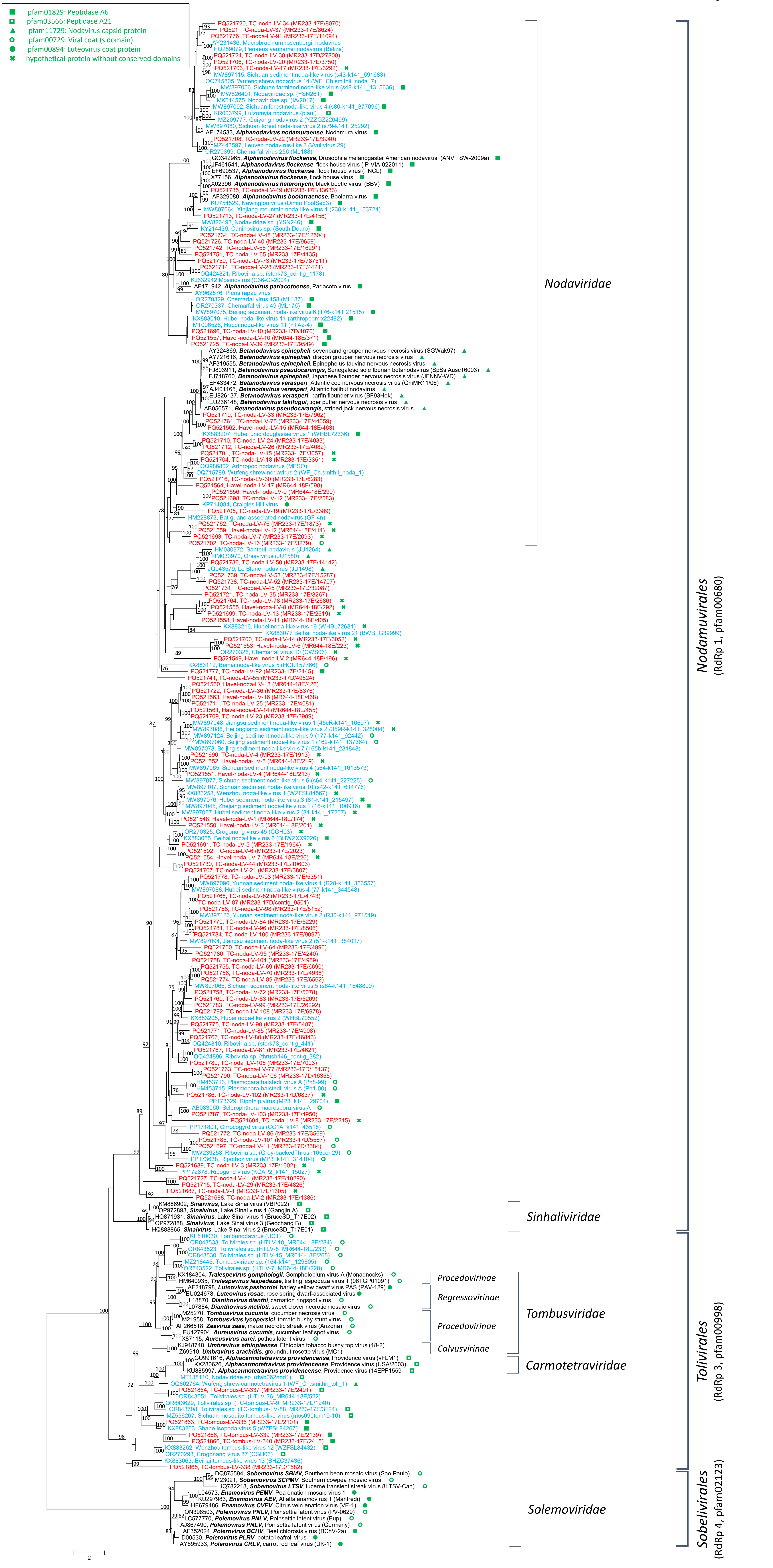

pfam01829: Peptidase A6
pfam03566: Peptidase A21
pfam11729: Nodavirus capsid protein
pfam00729: Viral coat (s domain)
pfam00894: Luteovirus coat protein
hypothetical protein without conserved domains
Nodaviridae
Nodamuvirales
(RdRp 1, pfam00680)
Sinhaliviridae
Procedovirinae
Regressovirinae
Procedovirinae
Calvusvirinae
Tombusviridae
Carmotetraviridae
Tolivirales
(RdRp 3, pfam00998)
Solemoviridae
Sobelivirales
(RdRp 4, pfam02123)
2

Figure S2

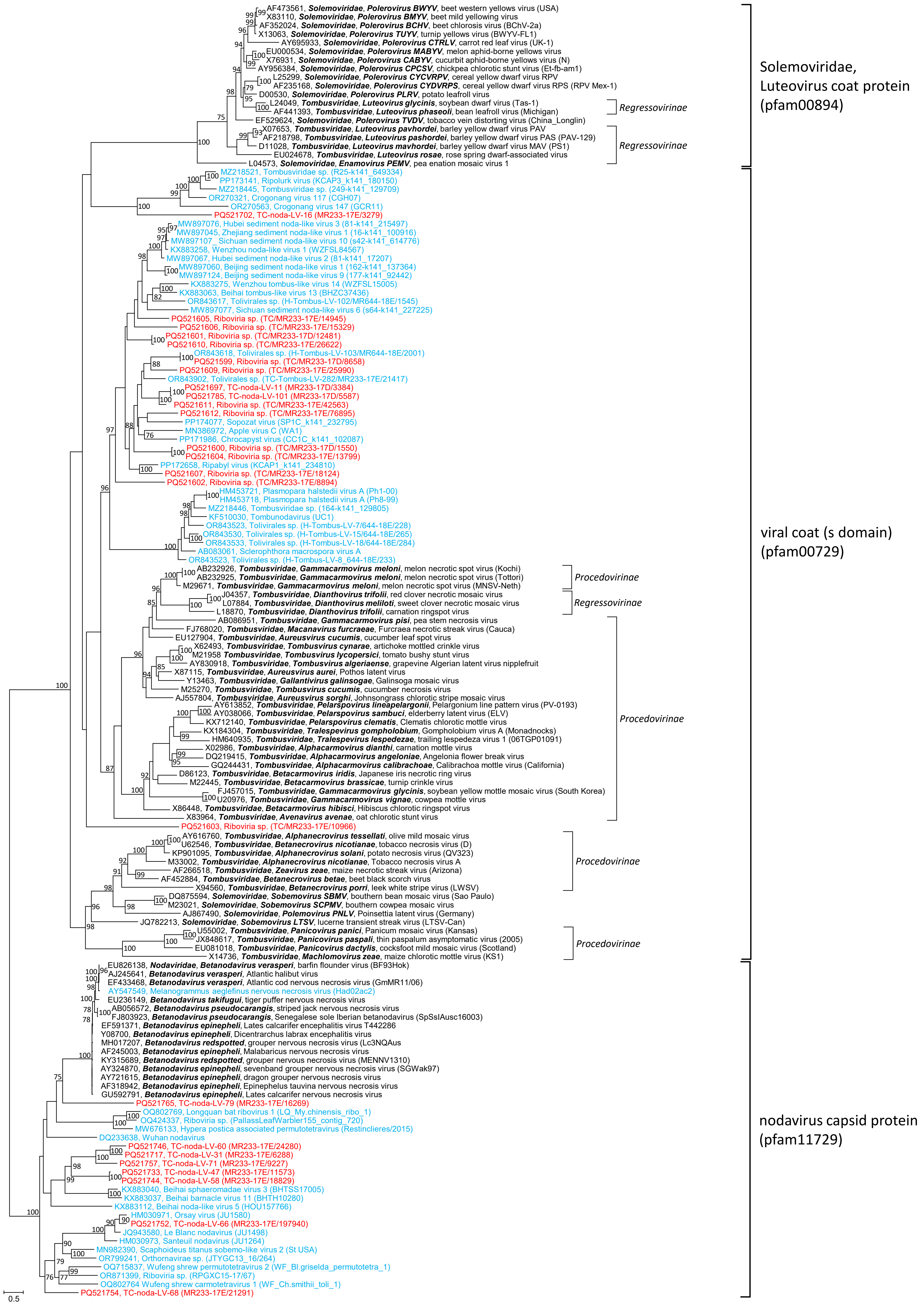
AF473561, Solemoviridae, Polerovirus BWYV, beet western yellows virus (USA)
X83110, Solemoviridae, Polerovirus BMYV, beet mild yellowing virus
AF352024, Solemoviridae, Polerovirus BCHV, beet chlorosis virus (BChV-2a)
X13063, Solemoviridae, Polerovirus TUYV, turnip yellows virus (BWYV-FL1)
AY695933, Solemoviridae, Polerovirus CTRLV, carrot red leaf virus (UK-1)
EU000534, Solemoviridae, Polerovirus MABYV, melon aphid-borne yellows virus
X76931, Solemoviridae, Polerovirus CABYV, cucurbit aphid-borne yellows virus (N)
AY956384, Solemoviridae, Polerovirus CPCSV, chickpea chlorotic stunt virus (Et-fb-am1)
L25299, Solemoviridae, Polerovirus CYCVRPV, cereal yellow dwarf virus RPV
AF235168, Solemoviridae, Polerovirus CYDVRPS, cereal yellow dwarf virus RPS (RPV Mex-1)
D00530, Solemoviridae, Polerovirus PLRV, potato leafroll virus
L24049, Tombusviridae, Luteovirus glycinis, soybean dwarf virus (Tas-1)
AF441393, Tombusviridae, Luteovirus phaseoli, bean leafroll virus (Michigan)
EF529624, Solemoviridae, Polerovirus TVDV, tobacco vein distorting virus (China_Longlin)
X07653, Tombusviridae, Luteovirus pavhordei, barley yellow dwarf virus PAV
AF218798, Tombusviridae, Luteovirus pashordei, barley yellow dwarf virus PAS (PAV-129)
D11028, Tombusviridae, Luteovirus mavhordei, barley yellow dwarf virus MAV (PS1)
EU024678, Tombusviridae, Luteovirus rosae, rose spring dwarf-associated virus
L04573, Solemoviridae, Enamovirus PEMV, pea enation mosaic virus 1
Regressovirinae
Regressovirinae
Solemoviridae,
Luteovirus coat protein
(pfam00894)
MZ218521, Tombusviridae sp. (R25-k141_649334)
PP173141, Ripolurk virus (KCAP3_k141_180150)
MZ218445, Tombusviridae sp. (249-k141_129709)
OR270321, Crogonang virus 117 (CGH07)
OR270563, Crogonang virus 147 (GCR11)
PQ521702, TC-noda-LV-16 (MR233-17E/3279)
MW897076, Hubei sediment noda-like virus 3 (81-k141_215497)
MW897045, Zhejiang sediment noda-like virus 1 (16-k141_100916)
MW897107_ Sichuan sediment noda-like virus 10 (s42-k141_614776)
KX883258, Wenzhou noda-like virus 1 (WZFSL84567)
MW897067, Hubei sediment noda-like virus 2 (81-k141_17207)
MW897060, Beijing sediment noda-like virus 1 (162-k141_137364)
MW897124, Beijing sediment noda-like virus 9 (177-k141_92442)
KX883275, Wenzhou tombus-like virus 14 (WZFSL15005)
KX883063, Beihai tombus-like virus 13 (BHZC37436)
OR843617, Tolivirales sp. (H-Tombus-LV-102/MR644-18E/1545)
MW897077, Sichuan sediment noda-like virus 6 (s64-k141_227225)
PQ521605, Riboviria sp. (TC/MR233-17E/14945)
PQ521606, Riboviria sp. (TC/MR233-17E/15329)
PQ521601, Riboviria sp. (TC/MR233-17D/12481)
PQ521610, Riboviria sp. (TC/MR233-17E/26622)
OR843618, Tolivirales sp. (H-Tombus-LV-103/MR644-18E/2001)
PQ521599, Riboviria sp. (TC/MR233-17D/8658)
PQ521609, Riboviria sp. (TC/MR233-17E/25990)
OR843902, Tolivirales sp. (TC-Tombus-LV-282/MR233-17E/21417)
PQ521697, TC-noda-LV-11 (MR233-17D/3384)
PQ521785, TC-noda-LV-101 (MR233-17D/5587)
PQ521611, Riboviria sp. (TC/MR233-17E/42563)
PQ521612, Riboviria sp. (TC/MR233-17E/76895)
PP174077, Sopozat virus (SP1C_k141_232795)
MN386972, Apple virus C (WA1)
PP171986, Chrocapyst virus (CC1C_k141_102087)
PQ521600, Riboviria sp. (TC/MR233-17D/1550)
PQ521604, Riboviria sp. (TC/MR233-17E/13799)
PP172658, Ripabyl virus (KCAP1_k141_234810)
PQ521607, Riboviria sp. (TC/MR233-17E/18124)
PQ521602, Riboviria sp. (TC/MR233-17E/8894)
HM453721, Plasmopara halstedii virus A (Ph1-00)
HM453718, Plasmopara halstedii virus A (Ph8-99)
MZ218446, Tombusviridae sp. (164-k141_129805)
KF510030, Tombunodavirus (UC1)
OR843523, Tolivirales sp. (H-Tombus-LV-7/644-18E/228)
OR843530, Tolivirales sp. (H-Tombus-LV-15/644-18E/265)
OR843533, Tolivirales sp. (H-Tombus-LV-18/644-18E/284)
AB083061, Sclerophthora macrospora virus A
OR843523, Tolivirales sp. (H-Tombus-LV-8_644-18E/233)
AB232926, Tombusviridae, Gammacarmovirus meloni, melon necrotic spot virus (Kochi)
AB232925, Tombusviridae, Gammacarmovirus meloni, melon necrotic spot virus (Tottori)
M29671, Tombusviridae, Gammacarmovirus meloni, melon necrotic spot virus (MNSV-Neth)
J04357, Tombusviridae, Dianthovirus trifolii, red clover necrotic mosaic virus
L07884, Tombusviridae, Dianthovirus meliloti, sweet clover necrotic mosaic virus
L18870, Tombusviridae, Dianthovirus trifolii, carnation ringspot virus
AB086951, Tombusviridae, Gammacarmovirus pisi, pea stem necrosis virus
FJ768020, Tombusviridae, Macanavirus furcraeae, Furcraea necrotic streak virus (Cauca)
EU127904, Tombusviridae, Aureusvirus cucumis, cucumber leaf spot virus
X62493, Tombusviridae, Tombusvirus cynarae, artichoke mottled crinkle virus
M21958 Tombusviridae, Tombusvirus lycopersici, tomato bushy stunt virus
AY830918, Tombusviridae, Tombusvirus algeriaense, grapevine Algerian latent virus nipplefruit
X87115, Tombusviridae, Aureusvirus aurei, Pothos latent virus
Y13463, Tombusviridae, Gallantivirus galinsogae, Galinsoga mosaic virus
M25270, Tombusviridae, Tombusvirus cucumis, cucumber necrosis virus
AJ557804, Tombusviridae, Aureusvirus sorghi, Johnsongrass chlorotic stripe mosaic virus
AY613852, Tombusviridae, Pelarspovirus lineapelargonii, Pelargonium line pattern virus (PV-0193)
AY038066, Tombusviridae, Pelarspovirus sambuci, elderberry latent virus (ELV)
KX712140, Tombusviridae, Pelarspovirus clematis, Clematis chlorotic mottle virus
KX184304, Tombusviridae, Tralespevirus gompholobium, Gompholobium virus A (Monadnocks)
HM640935, Tombusviridae, Tralespevirus lespedezae, trailing lespedeza virus 1 (06TGP01091)
X02986, Tombusviridae, Alphacarmovirus dianthi, carnation mottle virus
DQ219415, Tombusviridae, Alphacarmovirus angeloniae, Angelonia flower break virus
GQ244431, Tombusviridae, Alphacarmovirus calibrachoae, Calibrachoa mottle virus (California)
D86123, Tombusviridae, Betacarmovirus iridis, Japanese iris necrotic ring virus
M22445, Tombusviridae, Betacarmovirus brassicae, turnip crinkle virus
FJ457015, Tombusviridae, Gammacarmovirus glycinis, soybean yellow mottle mosaic virus (South Korea)
U20976, Tombusviridae, Gammacarmovirus vignae, cowpea mottle virus
X86448, Tombusviridae, Betacarmovirus hibisci, Hibiscus chlorotic ringspot virus
X83964, Tombusviridae, Avenavirus avenae, oat chlorotic stunt virus
PQ521603, Riboviria sp. (TC/MR233-17E/10966)
AY616760, Tombusviridae, Alphanecrovirus tessellati, olive mild mosaic virus
U62546, Tombusviridae, Betanecrovirus nicotianae, tobacco necrosis virus (D)
KP901095, Tombusviridae, Alphanecrovirus solani, potato necrosis virus (QV323)
M33002, Tombusviridae, Alphanecrovirus nicotianae, Tobacco necrosis virus A
AF266518, Tombusviridae, Zeavirus zeae, maize necrotic streak virus (Arizona)
AF452884, Tombusviridae, Betanecrovirus betae, beet black scorch virus
X94560, Tombusviridae, Betanecrovirus porri, leek white stripe virus (LWSV)
DQ875594, Solemoviridae, Sobemovirus SBMV, southern bean mosaic virus (Sao Paulo)
M23021, Solemoviridae, Sobemovirus SCPMV, southern cowpea mosaic virus
AJ867490, Solemoviridae, Polemovirus PNLV, Poinsettia latent virus (Germany)
JQ782213, Solemoviridae, Sobemovirus LTSV, lucerne transient streak virus (LTSV-Can)
U55002, Tombusviridae, Panicovirus panici, Panicum mosaic virus (Kansas)
JX848617, Tombusviridae, Panicovirus paspali, thin paspalum asymptomatic virus (2005)
EU081018, Tombusviridae, Panicovirus dactylis, cocksfoot mild mosaic virus (Scotland)
X14736, Tombusviridae, Machlomovirus zeae, maize chlorotic mottle virus (KS1)
Procedovirinae
Regressovirinae
Procedovirinae
Procedovirinae
Procedovirinae
viral coat (s domain)
(pfam00729)
EU826138, Nodaviridae, Betanodavirus verasperi, barfin flounder virus (BF93Hok)
AJ245641, Betanodavirus verasperi, Atlantic halibut virus
EF433468, Betanodavirus verasperi, Atlantic cod nervous necrosis virus (GmMR11/06)
AY547549, Melanogrammus aeglefinus nervous necrosis virus (Had02ac2)
EU236149, Betanodavirus takifugui, tiger puffer nervous necrosis virus
AB056572, Betanodavirus pseudocarangis, striped jack nervous necrosis virus
FJ803923, Betanodavirus pseudocarangis, Senegalese sole Iberian betanodavirus (SpSsIAusc16003)
EF591371, Betanodavirus epinepheli, Lates calcarifer encephalitis virus T442286
Y08700, Betanodavirus epinepheli, Dicentrarchus labrax encephalitis virus
MH017207, Betanodavirus redspotted, grouper nervous necrosis virus (Lc3NQAus
AF245003, Betanodavirus epinepheli, Malabaricus nervous necrosis virus
KY315689, Betanodavirus redspotted, grouper nervous necrosis virus (MENNV1310)
AY324870, Betanodavirus epinepheli, sevenband grouper nervous necrosis virus (SGWak97)
AY721615, Betanodavirus epinepheli, dragon grouper nervous necrosis virus
AF318942, Betanodavirus epinepheli, Epinephelus tauvina nervous necrosis virus
GU592791, Betanodavirus epinepheli, Lates calcarifer nervous necrosis virus
PQ521765, TC-noda-LV-79 (MR233-17E/16269)
OQ802769, Longquan bat ribovirus 1 (LQ_My.chinensis_ribo_1)
OQ424337, Riboviria sp. (PallassLeafWarbler155_contig_720)
MW676133, Hypera postica associated permutotetravirus (Restinclieres/2015)
DQ233638, Wuhan nodavirus
PQ521746, TC-noda-LV-60 (MR233-17E/24280)
PQ521717, TC-noda-LV-31 (MR233-17E/6288)
PQ521757, TC-noda-LV-71 (MR233-17E/9227)
PQ521733, TC-noda-LV-47 (MR233-17E/11573)
PQ521744, TC-noda-LV-58 (MR233-17E/18829)
KX883040, Beihai sphaeromadae virus 3 (BHTSS17005)
KX883037, Beihai barnacle virus 11 (BHTH10280)
KX883112, Beihai noda-like virus 5 (HOU157766)
HM030971, Orsay virus (JU1580)
PQ521752, TC-noda-LV-66 (MR233-17E/197940)
JQ943580, Le Blanc nodavirus (JU1498)
HM030973, Santeuil nodavirus (JU1264)
MN982390, Scaphoideus titanus sobemo-like virus 2 (St USA)
OR799241, Orthornavirae sp. (JTYGC13_16/264)
OQ715837, Wufeng shrew permutotetravirus 2 (WF_Bl.griselda_permutotetra_1)
OR871399, Riboviria sp. (RPGXC15-17/67)
OQ802764 Wufeng shrew carmotetravirus 1 (WF_Ch.smithii_toli_1)
PQ521754, TC-noda-LV-68 (MR233-17E/21291)
nodavirus capsid protein
(pfam11729)
0.5

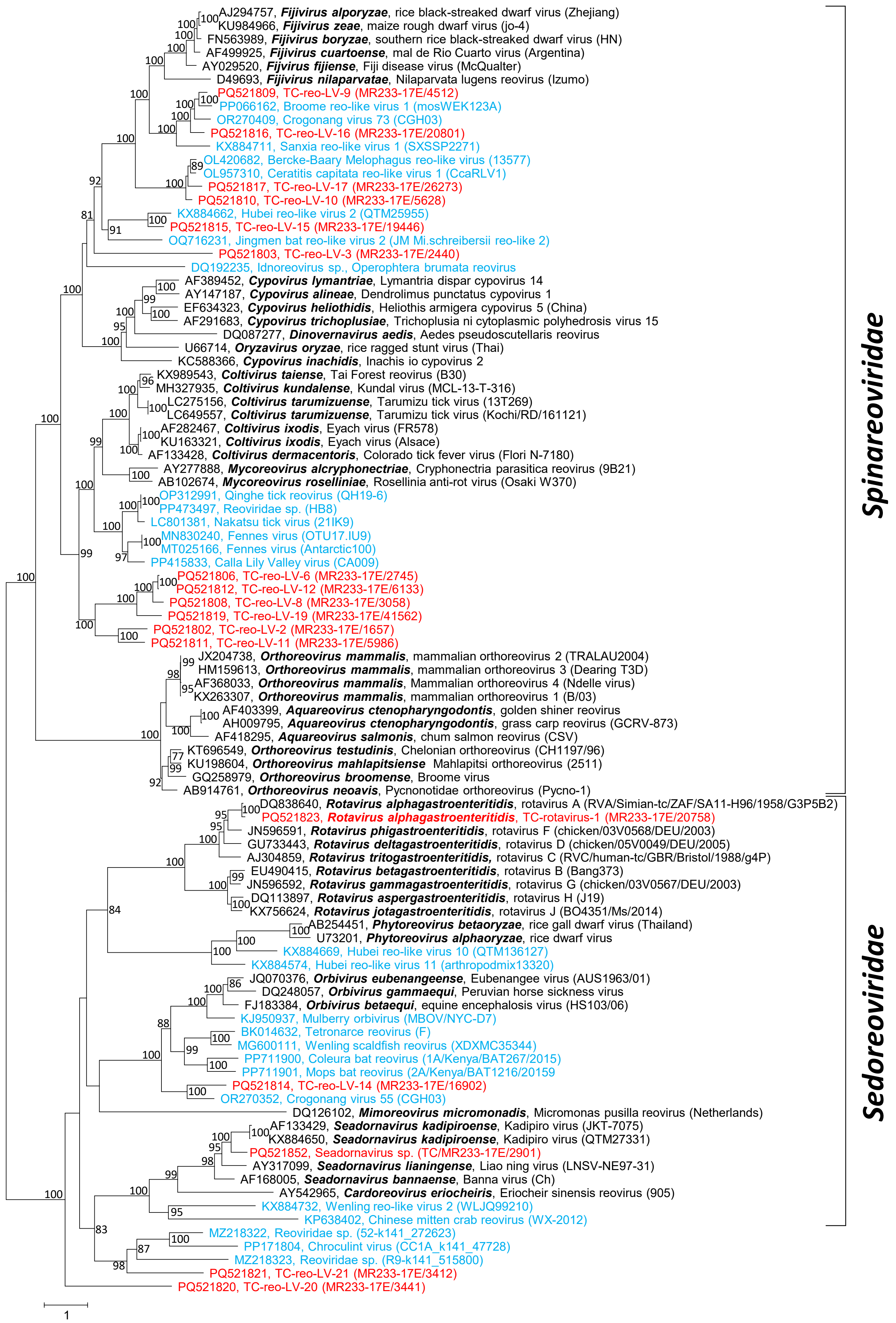
AJ294757, Fijivirus alporyzae, rice black-streaked dwarf virus (Zhejiang)
KU984966, Fijivirus zeae, maize rough dwarf virus (jo-4)
FN563989, Fijivirus boryzae, southern rice black-streaked dwarf virus (HN)
AF499925, Fijivirus cuartoense, mal de Rio Cuarto virus (Argentina)
AY029520, Fijvirus fijiense, Fiji disease virus (McQualter)
D49693, Fijivirus nilaparvatae, Nilaparvata lugens reovirus (Izumo)
PQ521809, TC-reo-LV-9 (MR233-17E/4512)
PP066162, Broome reo-like virus 1 (mosWEK123A)
OR270409, Crogonang virus 73 (CGH03)
PQ521816, TC-reo-LV-16 (MR233-17E/20801)
KX884711, Sanxia reo-like virus 1 (SXSSP2271)
OL420682, Bercke-Baary Melophagus reo-like virus (13577)
OL957310, Ceratitis capitata reo-like virus 1 (CcaRLV1)
PQ521817, TC-reo-LV-17 (MR233-17E/26273)
PQ521810, TC-reo-LV-10 (MR233-17E/5628)
KX884662, Hubei reo-like virus 2 (QTM25955)
PQ521815, TC-reo-LV-15 (MR233-17E/19446)
OQ716231, Jingmen bat reo-like virus 2 (JM Mi.schreibersii reo-like 2)
PQ521803, TC-reo-LV-3 (MR233-17E/2440)
DQ192235, Idnoreovirus sp., Operophtera brumata reovirus
AF389452, Cypovirus lymantriae, Lymantria dispar cypovirus 14
AY147187, Cypovirus alineae, Dendrolimus punctatus cypovirus 1
EF634323, Cypovirus heliothidis, Heliothis armigera cypovirus 5 (China)
AF291683, Cypovirus trichoplusiae, Trichoplusia ni cytoplasmic polyhedrosis virus 15
DQ087277, Dinovernavirus aedis, Aedes pseudoscutellaris reovirus
U66714, Oryzavirus oryzae, rice ragged stunt virus (Thai)
KC588366, Cypovirus inachidis, Inachis io cypovirus 2
KX989543, Coltivirus taiense, Tai Forest reovirus (B30)
MH327935, Coltivirus kundalense, Kundal virus (MCL-13-T-316)
LC275156, Coltivirus tarumizuense, Tarumizu tick virus (13T269)
LC649557, Coltivirus tarumizuense, Tarumizu tick virus (Kochi/RD/161121)
AF282467, Coltivirus ixodis, Eyach virus (FR578)
KU163321, Coltivirus ixodis, Eyach virus (Alsace)
AF133428, Coltivirus dermacentoris, Colorado tick fever virus (Flori N-7180)
AY277888, Mycoreovirus alcryphonectriae, Cryphonectria parasitica reovirus (9B21)
AB102674, Mycoreovirus roselliniae, Rosellinia anti-rot virus (Osaki W370)
OP312991, Qinghe tick reovirus (QH19-6)
PP473497, Reoviridae sp. (HB8)
LC801381, Nakatsu tick virus (21IK9)
MN830240, Fennes virus (OTU17.IU9)
MT025166, Fennes virus (Antarctic100)
PP415833, Calla Lily Valley virus (CA009)
PQ521806, TC-reo-LV-6 (MR233-17E/2745)
PQ521812, TC-reo-LV-12 (MR233-17E/6133)
PQ521808, TC-reo-LV-8 (MR233-17E/3058)
PQ521819, TC-reo-LV-19 (MR233-17E/41562)
PQ521802, TC-reo-LV-2 (MR233-17E/1657)
PQ521811, TC-reo-LV-11 (MR233-17E/5986)
JX204738, Orthoreovirus mammalis, mammalian orthoreovirus 2 (TRALAU2004)
HM159613, Orthoreovirus mammalis, mammalian orthoreovirus 3 (Dearing T3D)
AF368033, Orthoreovirus mammalis, Mammalian orthoreovirus 4 (Ndelle virus)
KX263307, Orthoreovirus mammalis, mammalian orthoreovirus 1 (B/03)
AF403399, Aquareovirus ctenopharyngodontis, golden shiner reovirus
AH009795, Aquareovirus ctenopharyngodontis, grass carp reovirus (GCRV-873)
AF418295, Aquareovirus salmonis, chum salmon reovirus (CSV)
KT696549, Orthoreovirus testudinis, Chelonian orthoreovirus (CH1197/96)
KU198604, Orthoreovirus mahlapitsiense Mahlapitsi orthoreovirus (2511)
GQ258979, Orthoreovirus broomense, Broome virus
AB914761, Orthoreovirus neoavis, Pycnonotidae orthoreovirus (Pycno-1)
Spinareoviridae
DQ838640, Rotavirus alphagastroenteritidis, rotavirus A (RVA/Simian-tc/ZAF/SA11-H96/1958/G3P5B2)
PQ521823, Rotavirus alphagastroenteritidis, TC-rotavirus-1 (MR233-17E/20758)
JN596591, Rotavirus phigastroenteritidis, rotavirus F (chicken/03V0568/DEU/2003)
GU733443, Rotavirus deltagastroenteritidis, rotavirus D (chicken/05V0049/DEU/2005)
AJ304859, Rotavirus tritogastroenteritidis, rotavirus C (RVC/human-tc/GBR/Bristol/1988/g4P)
EU490415, Rotavirus betagastroenteritidis, rotavirus B (Bang373)
JN596592, Rotavirus gammagastroenteritidis, rotavirus G (chicken/03V0567/DEU/2003)
DQ113897, Rotavirus aspergastroenteritidis, rotavirus H (J19)
KX756624, Rotavirus jotagastroenteritidis, rotavirus J (BO4351/Ms/2014)
AB254451, Phytoreovirus betaoryzae, rice gall dwarf virus (Thailand)
U73201, Phytoreovirus alphaoryzae, rice dwarf virus
KX884669, Hubei reo-like virus 10 (QTM136127)
KX884574, Hubei reo-like virus 11 (arthropodmix13320)
JQ070376, Orbivirus eubenangeense, Eubenangee virus (AUS1963/01)
DQ248057, Orbivirus gammaequi, Peruvian horse sickness virus
FJ183384, Orbivirus betaequi, equine encephalosis virus (HS103/06)
KJ950937, Mulberry orbivirus (MBOV/NYC-D7)
BK014632, Tetronarce reovirus (F)
MG600111, Wenling scaldfish reovirus (XDXMC35344)
PP711900, Coleura bat reovirus (1A/Kenya/BAT267/2015)
PP711901, Mops bat reovirus (2A/Kenya/BAT1216/20159
PQ521814, TC-reo-LV-14 (MR233-17E/16902)
OR270352, Crogonang virus 55 (CGH03)
DQ126102, Mimoreovirus micromonadis, Micromonas pusilla reovirus (Netherlands)
AF133429, Seadornavirus kadipiroense, Kadipiro virus (JKT-7075)
KX884650, Seadornavirus kadipiroense, Kadipiro virus (QTM27331)
PQ521852, Seadornavirus sp. (TC/MR233-17E/2901)
AY317099, Seadornavirus lianingense, Liao ning virus (LNSV-NE97-31)
AF168005, Seadornavirus bannaense, Banna virus (Ch)
AY542965, Cardoreovirus eriocheiris, Eriocheir sinensis reovirus (905)
KX884732, Wenling reo-like virus 2 (WLJQ99210)
KP638402, Chinese mitten crab reovirus (WX-2012)
MZ218322, Reoviridae sp. (52-k141_272623)
PP171804, Chroculint virus (CC1A_k141_47728)
MZ218323, Reoviridae sp. (R9-k141_515800)
PQ521821, TC-reo-LV-21 (MR233-17E/3412)
PQ521820, TC-reo-LV-20 (MR233-17E/3441)
Sedoreoviridae
1

Figure S4

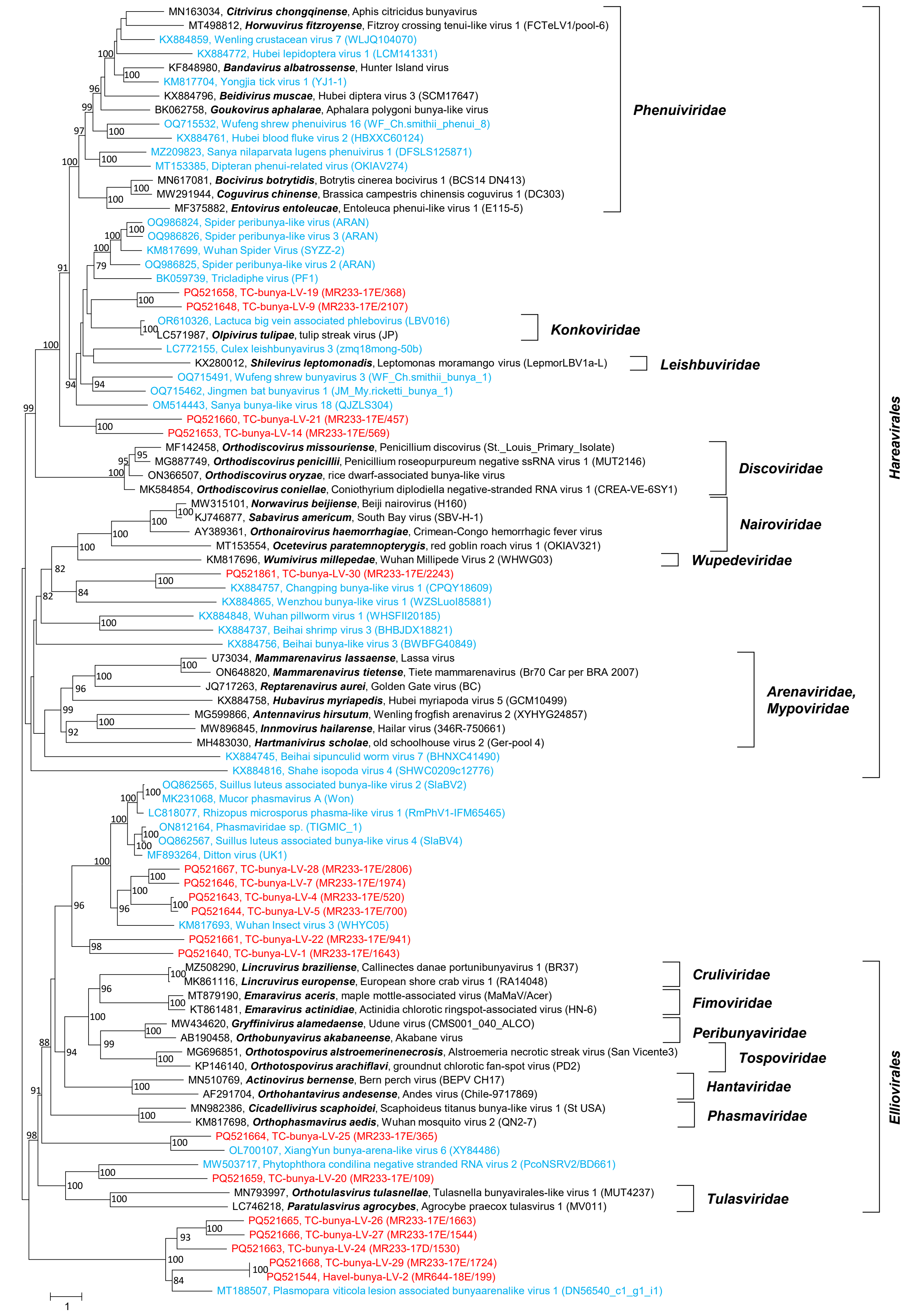
MN163034, Citrivirus chongqinense, Aphis citricidus bunyavirus
MT498812, Horwuvirus fitzroyense, Fitzroy crossing tenui-like virus 1 (FCTeLV1/pool-6)
KX884859, Wenling crustacean virus 7 (WLJQ104070)
KX884772, Hubei lepidoptera virus 1 (LCM141331)
KF848980, Bandavirus albatrossense, Hunter Island virus
KM817704, Yongjia tick virus 1 (YJ1-1)
KX884796, Beidivirus muscae, Hubei diptera virus 3 (SCM17647)
BK062758, Goukovirus aphalarae, Aphalara polygoni bunya-like virus
OQ715532, Wufeng shrew phenuivirus 16 (WF_Ch.smithii_phenui_8)
KX884761, Hubei blood fluke virus 2 (HBXXC60124)
MZ209823, Sanya nilaparvata lugens phenuivirus 1 (DFSLS125871)
MT153385, Dipteran phenui-related virus (OKIAV274)
MN617081, Bocivirus botrytidis, Botrytis cinerea bocivirus 1 (BCS14 DN413)
MW291944, Coguvirus chinense, Brassica campestris chinensis coguvirus 1 (DC303)
MF375882, Entovirus entoleucae, Entoleuca phenui-like virus 1 (E115-5)
Phenuiviridae
OQ986824, Spider peribunya-like virus (ARAN)
OQ986826, Spider peribunya-like virus 3 (ARAN)
KM817699, Wuhan Spider Virus (SYZZ-2)
OQ986825, Spider peribunya-like virus 2 (ARAN)
BK059739, Tricladiphe virus (PF1)
PQ521658, TC-bunya-LV-19 (MR233-17E/368)
PQ521648, TC-bunya-LV-9 (MR233-17E/2107)
OR610326, Lactuca big vein associated phlebovirus (LBV016)
LC571987, Olpivirus tulipae, tulip streak virus (JP)
Konkoviridae
LC772155, Culex leishbunyavirus 3 (zmq18mong-50b)
KX280012, Shilevirus leptomonadis, Leptomonas moramango virus (LepmorLBV1a-L)
Leishbuviridae
OQ715491, Wufeng shrew bunyavirus 3 (WF_Ch.smithii_bunya_1)
OQ715462, Jingmen bat bunyavirus 1 (JM_My.ricketti_bunya_1)
OM514443, Sanya bunya-like virus 18 (QJZLS304)
PQ521660, TC-bunya-LV-21 (MR233-17E/457)
PQ521653, TC-bunya-LV-14 (MR233-17E/569)
MF142458, Orthodiscovirus missouriense, Penicillium discovirus (St._Louis_Primary_Isolate)
MG887749, Orthodiscovirus penicillii, Penicillium roseopurpureum negative ssRNA virus 1 (MUT2146)
ON366507, Orthodiscovirus oryzae, rice dwarf-associated bunya-like virus
MK584854, Orthodiscovirus coniellae, Coniothyrium diplodiella negative-stranded RNA virus 1 (CREA-VE-6SY1)
Discoviridae
MW315101, Norwavirus beijiense, Beiji nairovirus (H160)
KJ746877, Sabavirus americum, South Bay virus (SBV-H-1)
AY389361, Orthonairovirus haemorrhagiae, Crimean-Congo hemorrhagic fever virus
MT153554, Ocetevirus paratemnopterygis, red goblin roach virus 1 (OKIAV321)
Nairoviridae
KM817696, Wumivirus millepedae, Wuhan Millipede Virus 2 (WHWG03)
Wupedeviridae
PQ521861, TC-bunya-LV-30 (MR233-17E/2243)
KX884757, Changping bunya-like virus 1 (CPQY18609)
KX884865, Wenzhou bunya-like virus 1 (WZSLuol85881)
KX884848, Wuhan pillworm virus 1 (WHSFII20185)
KX884737, Beihai shrimp virus 3 (BHBJDX18821)
KX884756, Beihai bunya-like virus 3 (BWBFG40849)
U73034, Mammarenavirus lassaense, Lassa virus
ON648820, Mammarenavirus tietense, Tiete mammarenavirus (Br70 Car per BRA 2007)
JQ717263, Reptarenavirus aurei, Golden Gate virus (BC)
KX884758, Hubavirus myriapedis, Hubei myriapoda virus 5 (GCM10499)
MG599866, Antennavirus hirsutum, Wenling frogfish arenavirus 2 (XYHYG24857)
MW896845, Innmovirus hailarense, Hailar virus (346R-750661)
MH483030, Hartmanivirus scholae, old schoolhouse virus 2 (Ger-pool 4)
Arenaviridae,
Mypoviridae
KX884745, Beihai sipunculid worm virus 7 (BHNXC41490)
KX884816, Shahe isopoda virus 4 (SHWC0209c12776)
Hareavirales
OQ862565, Suillus luteus associated bunya-like virus 2 (SlaBV2)
MK231068, Mucor phasmavirus A (Won)
LC818077, Rhizopus microsporus phasma-like virus 1 (RmPhV1-IFM65465)
ON812164, Phasmaviridae sp. (TIGMIC_1)
OQ862567, Suillus luteus associated bunya-like virus 4 (SlaBV4)
MF893264, Ditton virus (UK1)
PQ521667, TC-bunya-LV-28 (MR233-17E/2806)
PQ521646, TC-bunya-LV-7 (MR233-17E/1974)
PQ521643, TC-bunya-LV-4 (MR233-17E/520)
PQ521644, TC-bunya-LV-5 (MR233-17E/700)
KM817693, Wuhan Insect virus 3 (WHYC05)
PQ521661, TC-bunya-LV-22 (MR233-17E/941)
PQ521640, TC-bunya-LV-1 (MR233-17E/1643)
MZ508290, Lincruvirus braziliense, Callinectes danae portunibunyavirus 1 (BR37)
MK861116, Lincruvirus europense, European shore crab virus 1 (RA14048)
Cruliviridae
MT879190, Emaravirus aceris, maple mottle-associated virus (MaMaV/Acer)
KT861481, Emaravirus actinidiae, Actinidia chlorotic ringspot-associated virus (HN-6)
Fimoviridae
MW434620, Gryffinivirus alamedaense, Udune virus (CMS001_040_ALCO)
AB190458, Orthobunyavirus akabaneense, Akabane virus
Peribunyaviridae
MG696851, Orthotospovirus alstroemerinenecrosis, Alstroemeria necrotic streak virus (San Vicente3)
KP146140, Orthotospovirus arachiflavi, groundnut chlorotic fan-spot virus (PD2)
Tospoviridae
MN510769, Actinovirus bernense, Bern perch virus (BEPV CH17)
AF291704, Orthohantavirus andesense, Andes virus (Chile-9717869)
Hantaviridae
MN982386, Cicadellivirus scaphoidei, Scaphoideus titanus bunya-like virus 1 (St USA)
KM817698, Orthophasmavirus aedis, Wuhan mosquito virus 2 (QN2-7)
Phasmaviridae
PQ521664, TC-bunya-LV-25 (MR233-17E/365)
OL700107, XiangYun bunya-arena-like virus 6 (XY84486)
MW503717, Phytophthora condilina negative stranded RNA virus 2 (PcoNSRV2/BD661)
PQ521659, TC-bunya-LV-20 (MR233-17E/109)
MN793997, Orthotulasvirus tulasnellae, Tulasnella bunyavirales-like virus 1 (MUT4237)
LC746218, Paratulasvirus agrocybes, Agrocybe praecox tulasvirus 1 (MV011)
Tulasviridae
Elliovirales
PQ521665, TC-bunya-LV-26 (MR233-17E/1663)
PQ521666, TC-bunya-LV-27 (MR233-17E/1544)
PQ521663, TC-bunya-LV-24 (MR233-17D/1530)
PQ521668, TC-bunya-LV-29 (MR233-17E/1724)
PQ521544, Havel-bunya-LV-2 (MR644-18E/199)
MT188507, Plasmopara viticola lesion associated bunyaarenalike virus 1 (DN56540_c1_g1_i1)
1

Figure S5A

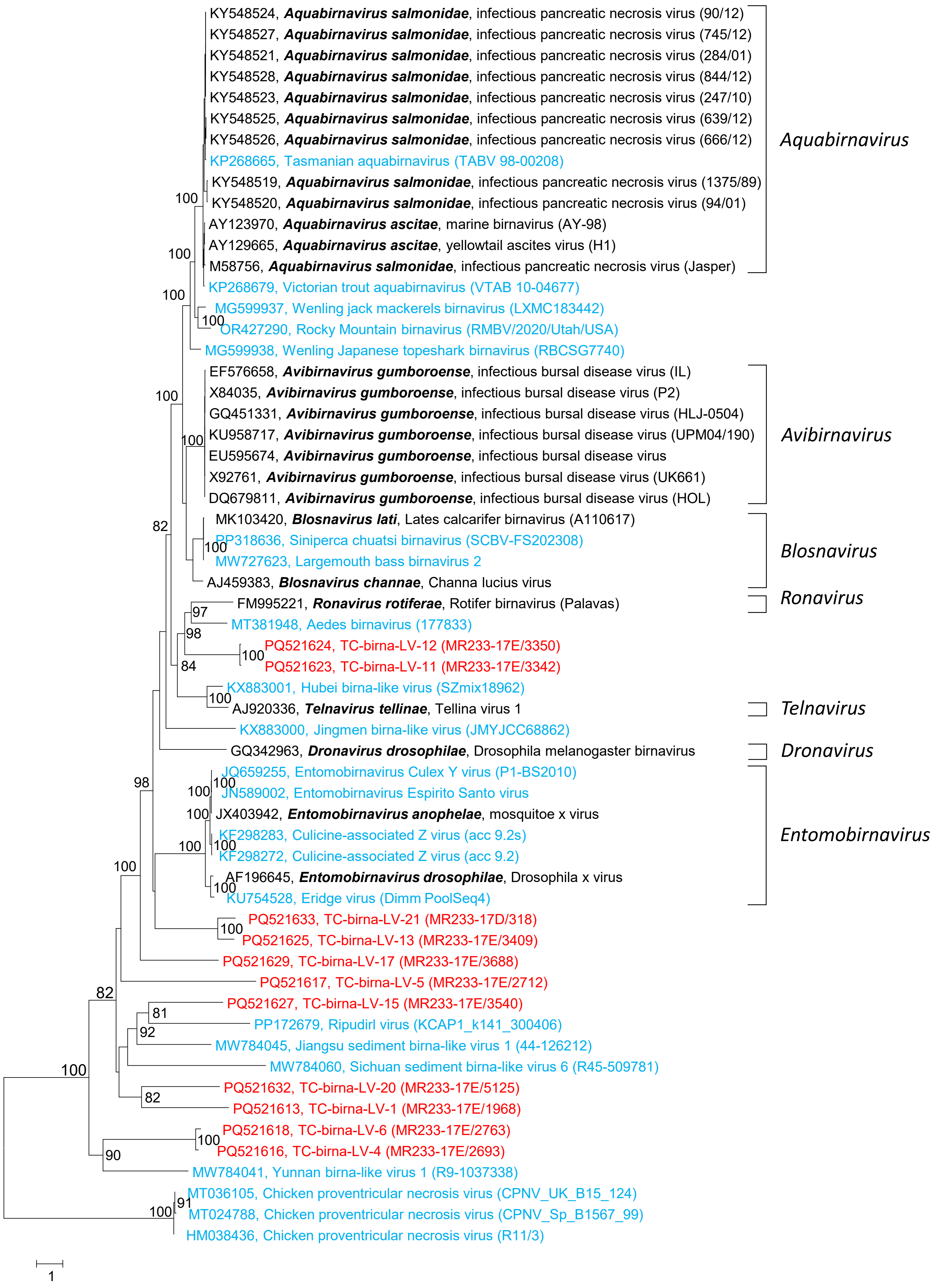

KY548524, **Aquabirnavirus salmonidae**, infectious pancreatic necrosis virus (90/12)
KY548527, **Aquabirnavirus salmonidae**, infectious pancreatic necrosis virus (745/12)
KY548521, **Aquabirnavirus salmonidae**, infectious pancreatic necrosis virus (284/01)
KY548528, **Aquabirnavirus salmonidae**, infectious pancreatic necrosis virus (844/12)
KY548523, **Aquabirnavirus salmonidae**, infectious pancreatic necrosis virus (247/10)
KY548525, **Aquabirnavirus salmonidae**, infectious pancreatic necrosis virus (639/12)
KY548526, **Aquabirnavirus salmonidae**, infectious pancreatic necrosis virus (666/12)
KP268665, Tasmanian aquabirnavirus (TABV 98-00208)
KY548519, **Aquabirnavirus salmonidae**, infectious pancreatic necrosis virus (1375/89)
KY548520, **Aquabirnavirus salmonidae**, infectious pancreatic necrosis virus (94/01)
AY123970, **Aquabirnavirus ascitae**, marine birnavirus (AY-98)
AY129665, **Aquabirnavirus ascitae**, yellowtail ascites virus (H1)
M58756, **Aquabirnavirus salmonidae**, infectious pancreatic necrosis virus (Jasper)
KP268679, Victorian trout aquabirnavirus (VTAB 10-04677)
MG599937, Wenling jack mackerels birnavirus (LXMC183442)
OR427290, Rocky Mountain birnavirus (RMBV/2020/Utah/USA)
MG599938, Wenling Japanese topeshark birnavirus (RBCSG7740)
EF576658, **Avibirnavirus gumboroense**, infectious bursal disease virus (IL)
X84035, **Avibirnavirus gumboroense**, infectious bursal disease virus (P2)
GQ451331, **Avibirnavirus gumboroense**, infectious bursal disease virus (HLJ-0504)
KU958717, **Avibirnavirus gumboroense**, infectious bursal disease virus (UPM04/190)
EU595674, **Avibirnavirus gumboroense**, infectious bursal disease virus
X92761, **Avibirnavirus gumboroense**, infectious bursal disease virus (UK661)
DQ679811, **Avibirnavirus gumboroense**, infectious bursal disease virus (HOL)
MK103420, **Blosnavirus lati**, Lates calcarifer birnavirus (A110617)
PP318636, Siniperca chuatsi birnavirus (SCBV-FS202308)
MW727623, Largemouth bass birnavirus 2
AJ459383, **Blosnavirus channae**, Channa lucius virus
FM995221, **Ronavirus rotiferae**, Rotifer birnavirus (Palavas)
MT381948, Aedes birnavirus (177833)
PQ521624, TC-birna-LV-12 (MR233-17E/3350)
PQ521623, TC-birna-LV-11 (MR233-17E/3342)
KX883001, Hubei birna-like virus (SZmix18962)
AJ920336, **Telnavirus tellinae**, Tellina virus 1
KX883000, Jingmen birna-like virus (JMYJCC68862)
GQ342963, **Dronavirus drosophilae**, Drosophila melanogaster birnavirus
JQ659255, Entomobirnavirus Culex Y virus (P1-BS2010)
JN589002, Entomobirnavirus Espirito Santo virus
JX403942, **Entomobirnavirus anophelae**, mosquitoe x virus
KF298283, Culicine-associated Z virus (acc 9.2s)
KF298272, Culicine-associated Z virus (acc 9.2)
AF196645, **Entomobirnavirus drosophilae**, Drosophila x virus
KU754528, Eridge virus (Dimm PoolSeq4)
PQ521633, TC-birna-LV-21 (MR233-17D/318)
PQ521625, TC-birna-LV-13 (MR233-17E/3409)
PQ521629, TC-birna-LV-17 (MR233-17E/3688)
PQ521617, TC-birna-LV-5 (MR233-17E/2712)
PQ521627, TC-birna-LV-15 (MR233-17E/3540)
PP172679, Ripudirl virus (KCAP1_k141_300406)
MW784045, Jiangsu sediment birna-like virus 1 (44-126212)
MW784060, Sichuan sediment birna-like virus 6 (R45-509781)
PQ521632, TC-birna-LV-20 (MR233-17E/5125)
PQ521613, TC-birna-LV-1 (MR233-17E/1968)
PQ521618, TC-birna-LV-6 (MR233-17E/2763)
PQ521616, TC-birna-LV-4 (MR233-17E/2693)
MW784041, Yunnan birna-like virus 1 (R9-1037338)
MT036105, Chicken proventricular necrosis virus (CPNV_UK_B15_124)
MT024788, Chicken proventricular necrosis virus (CPNV_Sp_B1567_99)
HM038436, Chicken proventricular necrosis virus (R11/3)
Aquabirnavirus
Avibirnavirus
Blosnavirus
Ronavirus
Telnavirus
Dronavirus
Entomobirnavirus
100
100
100
100
100
100
82
100
97
98
84
100
100
98
100
100
100
100
100
100
100
100
82
81
92
82
100
100
90
100
91
1

Figure S5B

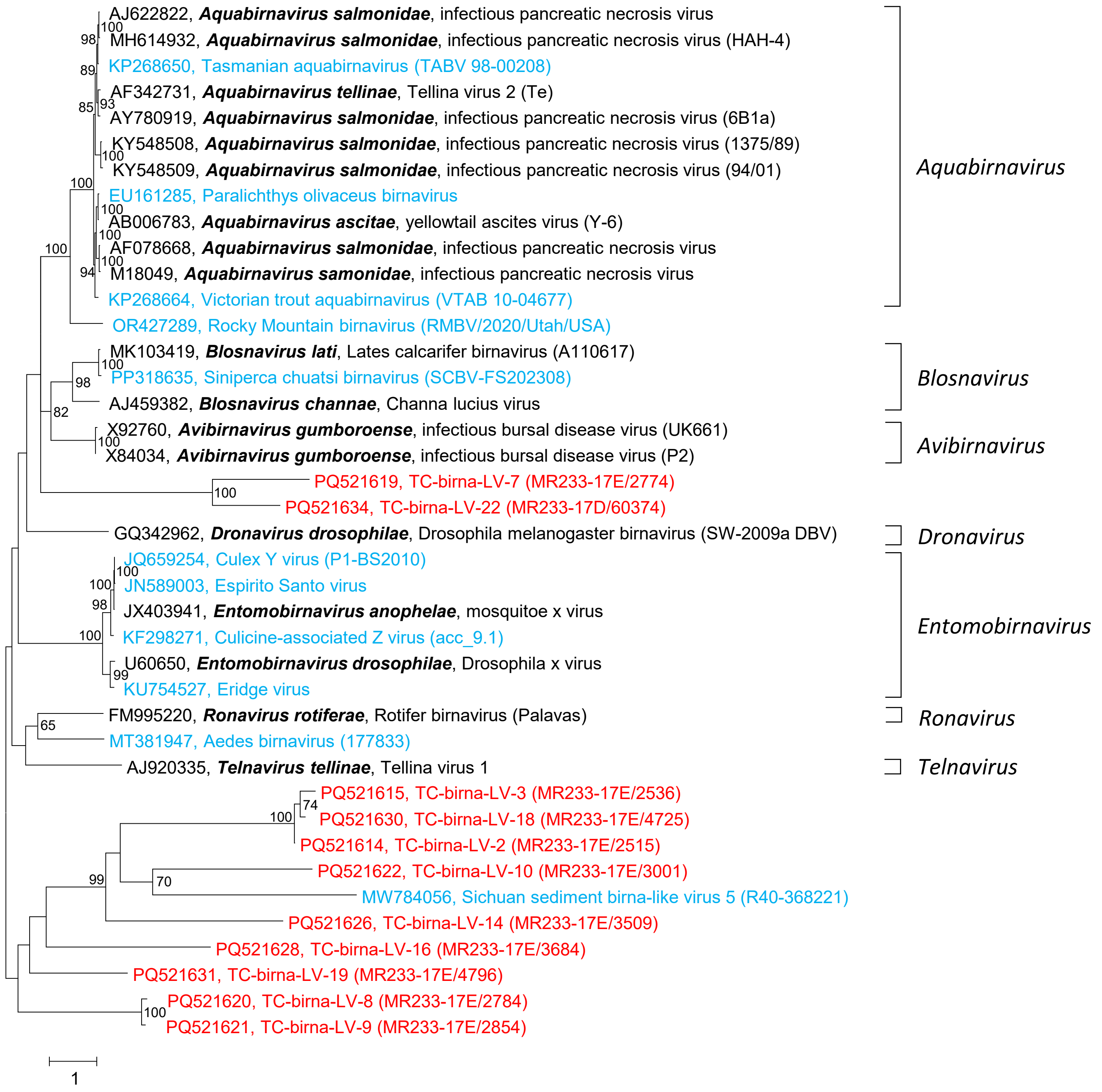

AJ622822, ***Aquabirnavirus salmonidae***, infectious pancreatic necrosis virus
MH614932, ***Aquabirnavirus salmonidae***, infectious pancreatic necrosis virus (HAH-4)
KP268650, Tasmanian aquabirnavirus (TABV 98-00208)
AF342731, ***Aquabirnavirus tellinae***, Tellina virus 2 (Te)
AY780919, ***Aquabirnavirus salmonidae***, infectious pancreatic necrosis virus (6B1a)
KY548508, ***Aquabirnavirus salmonidae***, infectious pancreatic necrosis virus (1375/89)
KY548509, ***Aquabirnavirus salmonidae***, infectious pancreatic necrosis virus (94/01)
EU161285, Paralichthys olivaceus birnavirus
AB006783, ***Aquabirnavirus ascitae***, yellowtail ascites virus (Y-6)
AF078668, ***Aquabirnavirus salmonidae***, infectious pancreatic necrosis virus
M18049, ***Aquabirnavirus samonidae***, infectious pancreatic necrosis virus
KP268664, Victorian trout aquabirnavirus (VTAB 10-04677)
OR427289, Rocky Mountain birnavirus (RMBV/2020/Utah/USA)
MK103419, ***Blosnavirus lati***, Lates calcarifer birnavirus (A110617)
PP318635, Siniperca chuatsi birnavirus (SCBV-FS202308)
AJ459382, ***Blosnavirus channae***, Channa lucius virus
X92760, ***Avibirnavirus gumboroense***, infectious bursal disease virus (UK661)
X84034, ***Avibirnavirus gumboroense***, infectious bursal disease virus (P2)
PQ521619, TC-birna-LV-7 (MR233-17E/2774)
PQ521634, TC-birna-LV-22 (MR233-17D/60374)
GQ342962, ***Dronavirus drosophilae***, Drosophila melanogaster birnavirus (SW-2009a DBV)
JQ659254, Culex Y virus (P1-BS2010)
JN589003, Espirito Santo virus
JX403941, ***Entomobirnavirus anophelae***, mosquitoe x virus
KF298271, Culicine-associated Z virus (acc_9.1)
U60650, ***Entomobirnavirus drosophilae***, Drosophila x virus
KU754527, Eridge virus
FM995220, ***Ronavirus rotiferae***, Rotifer birnavirus (Palavas)
MT381947, Aedes birnavirus (177833)
AJ920335, ***Telnavirus tellinae***, Tellina virus 1
PQ521615, TC-birna-LV-3 (MR233-17E/2536)
PQ521630, TC-birna-LV-18 (MR233-17E/4725)
PQ521614, TC-birna-LV-2 (MR233-17E/2515)
PQ521622, TC-birna-LV-10 (MR233-17E/3001)
MW784056, Sichuan sediment birna-like virus 5 (R40-368221)
PQ521626, TC-birna-LV-14 (MR233-17E/3509)
PQ521628, TC-birna-LV-16 (MR233-17E/3684)
PQ521631, TC-birna-LV-19 (MR233-17E/4796)
PQ521620, TC-birna-LV-8 (MR233-17E/2784)
PQ521621, TC-birna-LV-9 (MR233-17E/2854)
Aquabirnavirus
Blosnavirus
Avibirnavirus
Dronavirus
Entomobirnavirus
Ronavirus
Telnavirus
100 98 89 93 85 100 100 100 100 100 100 94 98 82 100 100 100 100 98 100 99 65 74 100 70 99 100
1

Figure S6

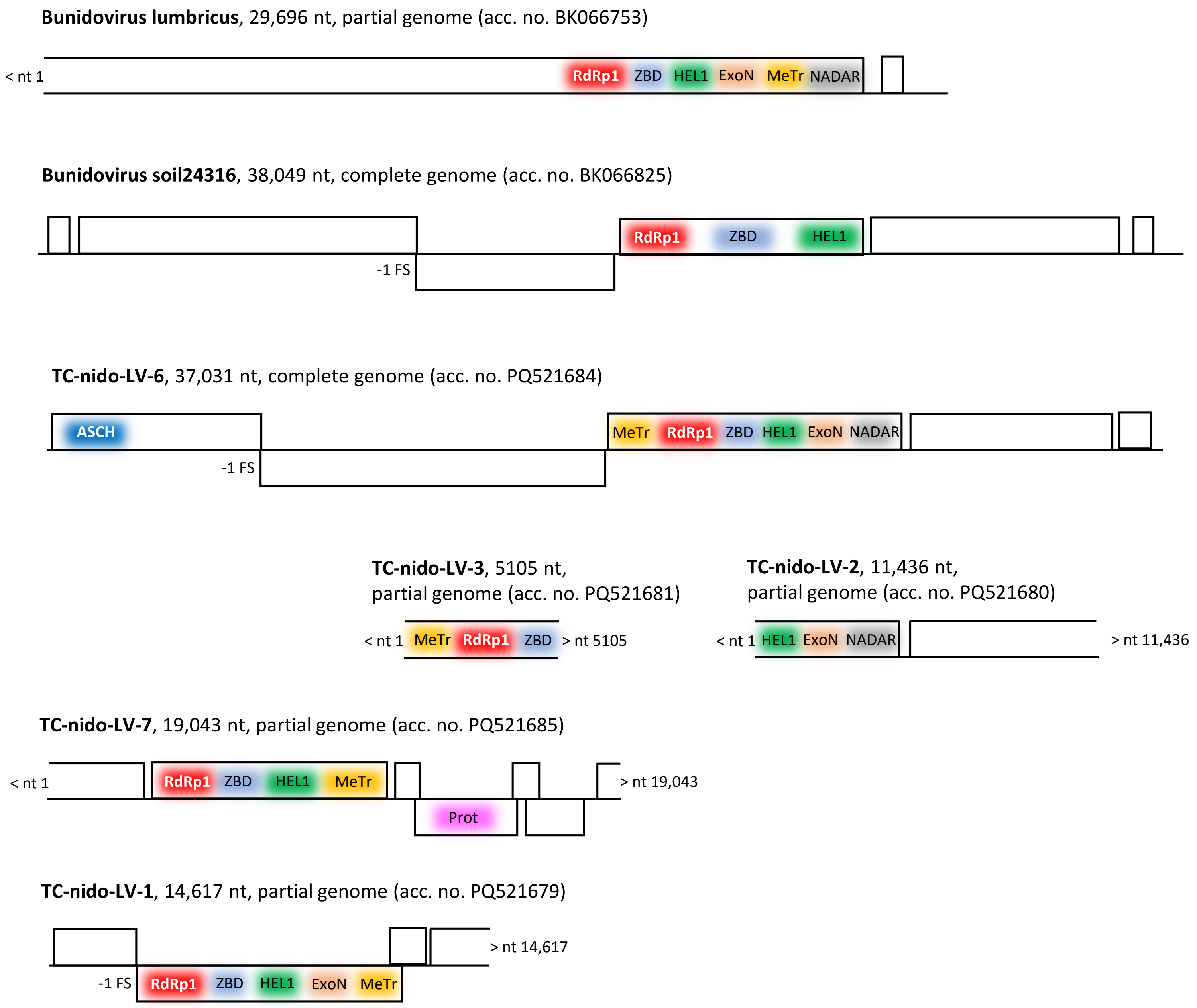

Bunidovirus lumbricus, 29,696 nt, partial genome (acc. no. BK066753)
< nt 1
RdRp1 ZBD HEL1 ExoN MeTr NADAR
Bunidovirus soil24316, 38,049 nt, complete genome (acc. no. BK066825)
-1 FS
RdRp1 ZBD HEL1
TC-nido-LV-6, 37,031 nt, complete genome (acc. no. PQ521684)
ASCH
-1 FS
MeTr RdRp1 ZBD HEL1 ExoN NADAR
TC-nido-LV-3, 5105 nt, partial genome (acc. no. PQ521681)
< nt 1 MeTr RdRp1 ZBD > nt 5105
TC-nido-LV-2, 11,436 nt, partial genome (acc. no. PQ521680)
< nt 1 HEL1 ExoN NADAR
> nt 11,436
TC-nido-LV-7, 19,043 nt, partial genome (acc. no. PQ521685)
< nt 1
RdRp1 ZBD HEL1 MeTr
Prot
> nt 19,043
TC-nido-LV-1, 14,617 nt, partial genome (acc. no. PQ521679)
> nt 14,617
-1 FS
RdRp1 ZBD HEL1 ExoN MeTr

Figure S7A

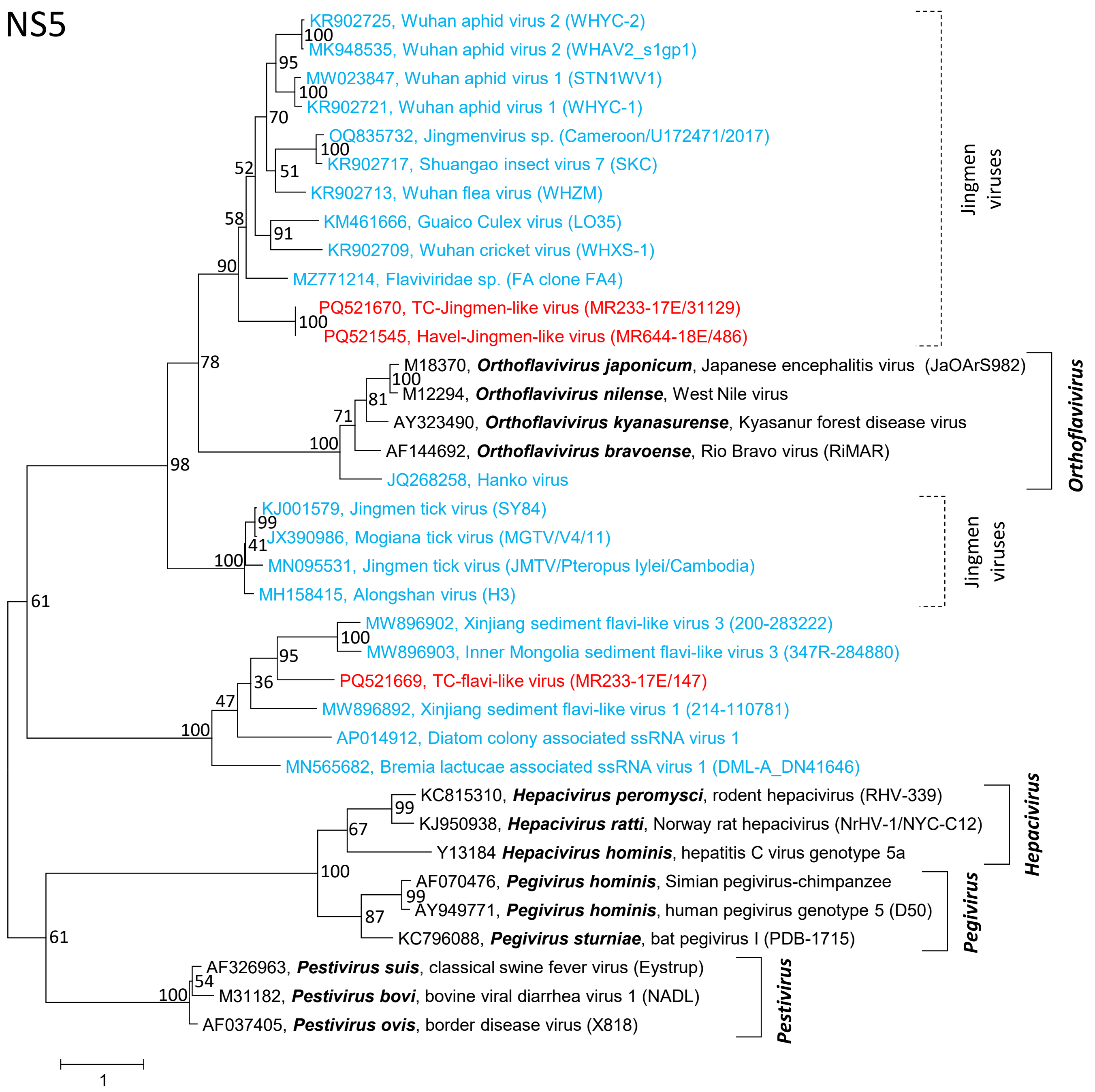
NS5
KR902725, Wuhan aphid virus 2 (WHYC-2)
MK948535, Wuhan aphid virus 2 (WHAV2_s1gp1)
MW023847, Wuhan aphid virus 1 (STN1WV1)
KR902721, Wuhan aphid virus 1 (WHYC-1)
OQ835732, Jingmenvirus sp. (Cameroon/U172471/2017)
KR902717, Shuangao insect virus 7 (SKC)
KR902713, Wuhan flea virus (WHZM)
KM461666, Guaico Culex virus (LO35)
KR902709, Wuhan cricket virus (WHXS-1)
MZ771214, Flaviviridae sp. (FA clone FA4)
PQ521670, TC-Jingmen-like virus (MR233-17E/31129)
PQ521545, Havel-Jingmen-like virus (MR644-18E/486)
M18370, Orthoflavivirus japonicum, Japanese encephalitis virus (JaOArS982)
M12294, Orthoflavivirus nilense, West Nile virus
AY323490, Orthoflavivirus kyanasurense, Kyasanur forest disease virus
AF144692, Orthoflavivirus bravoense, Rio Bravo virus (RiMAR)
JQ268258, Hanko virus
KJ001579, Jingmen tick virus (SY84)
JX390986, Mogiana tick virus (MGTV/V4/11)
MN095531, Jingmen tick virus (JMTV/Pteropus lylei/Cambodia)
MH158415, Alongshan virus (H3)
MW896902, Xinjiang sediment flavi-like virus 3 (200-283222)
MW896903, Inner Mongolia sediment flavi-like virus 3 (347R-284880)
PQ521669, TC-flavi-like virus (MR233-17E/147)
MW896892, Xinjiang sediment flavi-like virus 1 (214-110781)
AP014912, Diatom colony associated ssRNA virus 1
MN565682, Bremia lactucae associated ssRNA virus 1 (DML-A_DN41646)
KC815310, Hepacivirus peromysci, rodent hepacivirus (RHV-339)
KJ950938, Hepacivirus ratti, Norway rat hepacivirus (NrHV-1/NYC-C12)
Y13184 Hepacivirus hominis, hepatitis C virus genotype 5a
AF070476, Pegivirus hominis, Simian pegivirus-chimpanzee
AY949771, Pegivirus hominis, human pegivirus genotype 5 (D50)
KC796088, Pegivirus sturniae, bat pegivirus I (PDB-1715)
AF326963, Pestivirus suis, classical swine fever virus (Eystrup)
M31182, Pestivirus bovi, bovine viral diarrhea virus 1 (NADL)
AF037405, Pestivirus ovis, border disease virus (X818)
Jingmen viruses
Orthoflavivirus
Jingmen viruses
Hepacivirus
Pegivirus
Pestivirus
100
95
100
70
100
51
52
91
58
90
100
78
100
81
71
100
98
99
41
100
61
100
95
36
47
100
99
67
100
99
87
61
54
100
1

Figure S7B

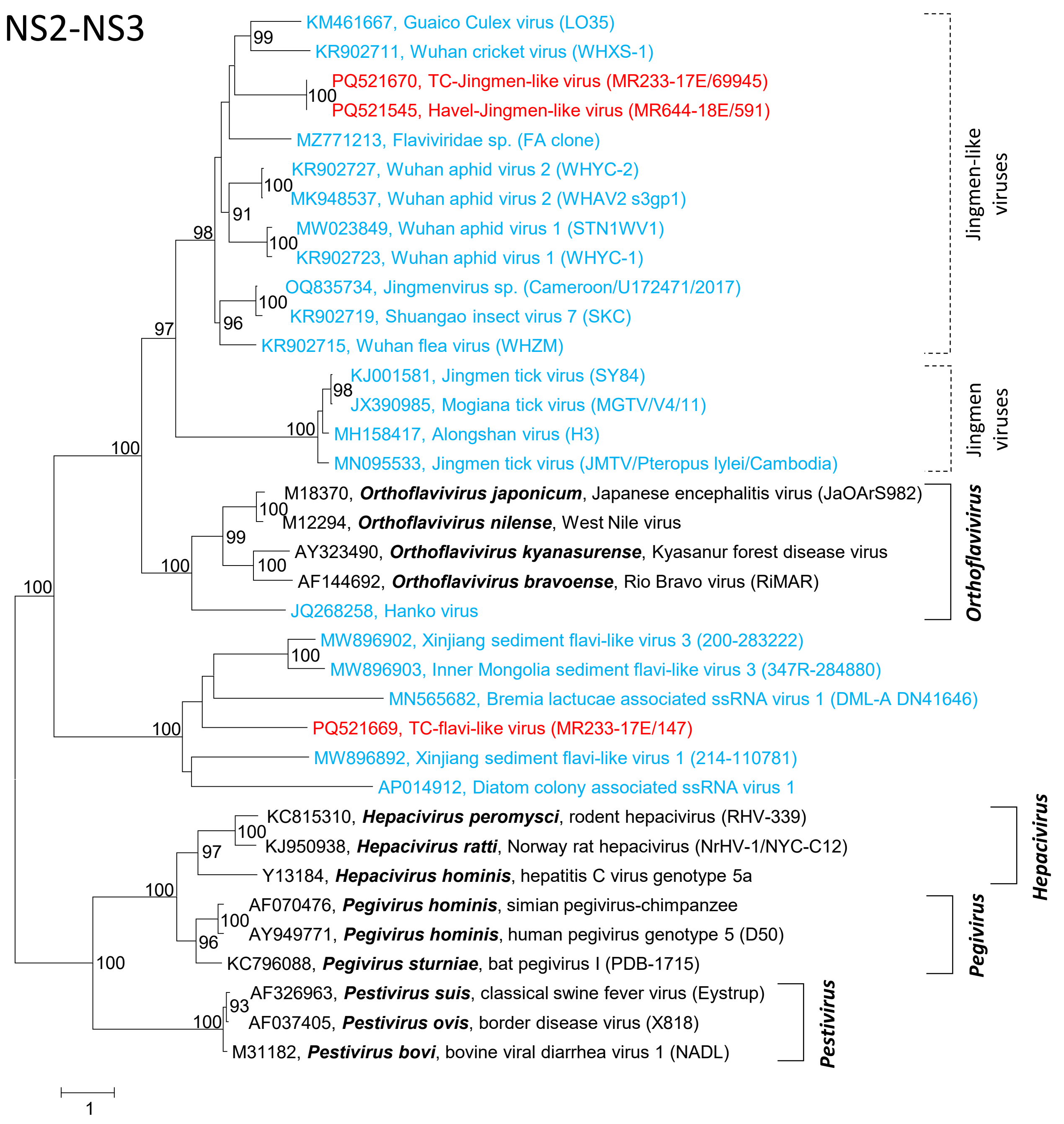

NS2-NS3
KM461667, Guaico Culex virus (LO35)
KR902711, Wuhan cricket virus (WHXS-1)
PQ521670, TC-Jingmen-like virus (MR233-17E/69945)
PQ521545, Havel-Jingmen-like virus (MR644-18E/591)
MZ771213, Flaviviridae sp. (FA clone)
KR902727, Wuhan aphid virus 2 (WHYC-2)
MK948537, Wuhan aphid virus 2 (WHAV2 s3gp1)
MW023849, Wuhan aphid virus 1 (STN1WV1)
KR902723, Wuhan aphid virus 1 (WHYC-1)
OQ835734, Jingmenvirus sp. (Cameroon/U172471/2017)
KR902719, Shuangao insect virus 7 (SKC)
KR902715, Wuhan flea virus (WHZM)
KJ001581, Jingmen tick virus (SY84)
JX390985, Mogiana tick virus (MGTV/V4/11)
MH158417, Alongshan virus (H3)
MN095533, Jingmen tick virus (JMTV/Pteropus lylei/Cambodia)
M18370, Orthoflavivirus japonicum, Japanese encephalitis virus (JaOArS982)
M12294, Orthoflavivirus nilense, West Nile virus
AY323490, Orthoflavivirus kyanasurense, Kyasanur forest disease virus
AF144692, Orthoflavivirus bravoense, Rio Bravo virus (RiMAR)
JQ268258, Hanko virus
MW896902, Xinjiang sediment flavi-like virus 3 (200-283222)
MW896903, Inner Mongolia sediment flavi-like virus 3 (347R-284880)
MN565682, Bremia lactucae associated ssRNA virus 1 (DML-A DN41646)
PQ521669, TC-flavi-like virus (MR233-17E/147)
MW896892, Xinjiang sediment flavi-like virus 1 (214-110781)
AP014912, Diatom colony associated ssRNA virus 1
KC815310, Hepacivirus peromysci, rodent hepacivirus (RHV-339)
KJ950938, Hepacivirus ratti, Norway rat hepacivirus (NrHV-1/NYC-C12)
Y13184, Hepacivirus hominis, hepatitis C virus genotype 5a
AF070476, Pegivirus hominis, simian pegivirus-chimpanzee
AY949771, Pegivirus hominis, human pegivirus genotype 5 (D50)
KC796088, Pegivirus sturniae, bat pegivirus I (PDB-1715)
AF326963, Pestivirus suis, classical swine fever virus (Eystrup)
AF037405, Pestivirus ovis, border disease virus (X818)
M31182, Pestivirus bovi, bovine viral diarrhea virus 1 (NADL)
Jingmen-like viruses
Jingmen viruses
Orthoflavivirus
Hepacivirus
Pegivirus
Pestivirus
99
100
91
100
100
98
96
100
97
98
100
100
100
99
100
100
100
100
100
97
100
100
96
100
93
100
100
1

Figure S8

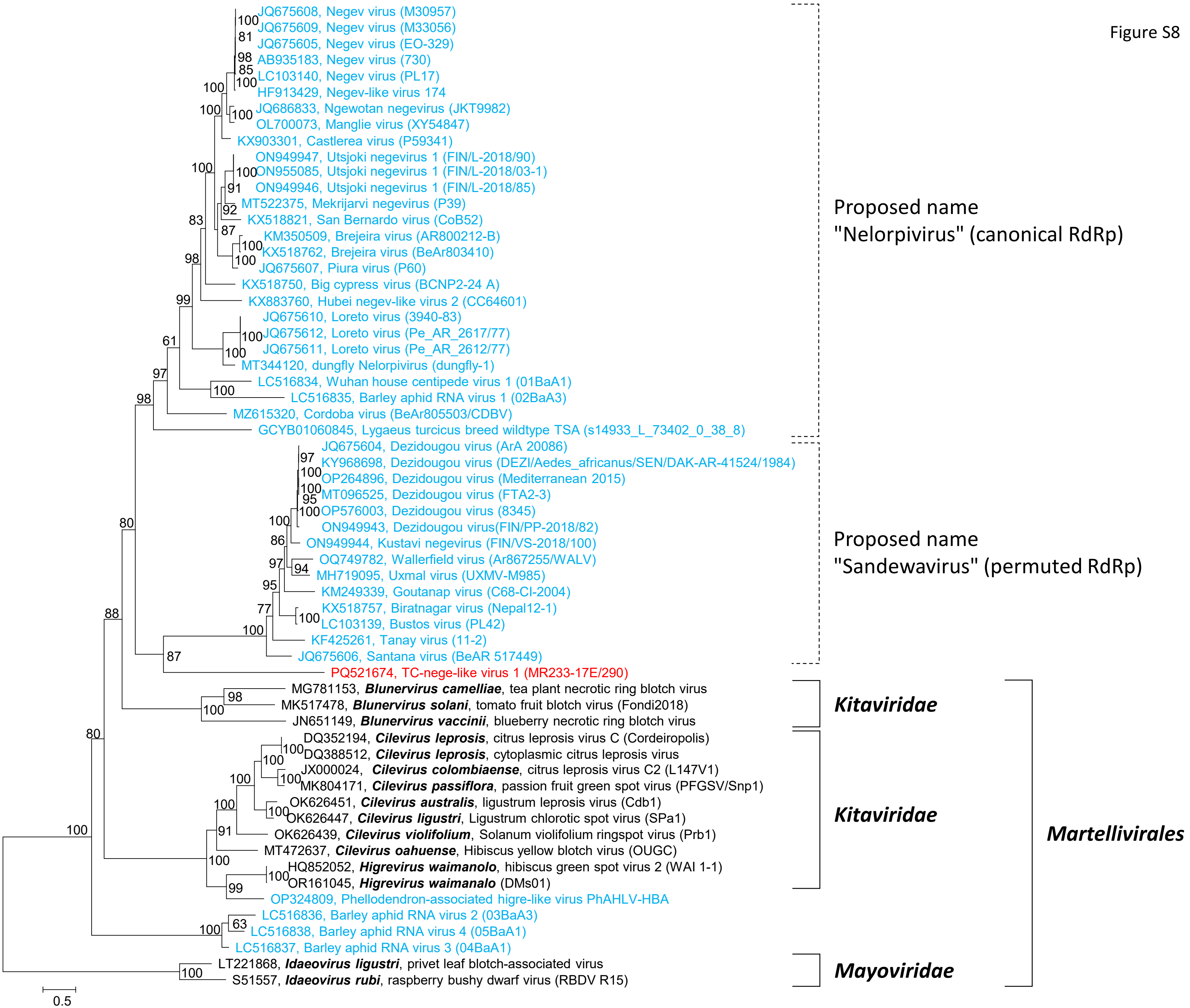

JQ675608, Negev virus (M30957)
JQ675609, Negev virus (M33056)
JQ675605, Negev virus (EO-329)
AB935183, Negev virus (730)
LC103140, Negev virus (PL17)
HF913429, Negev-like virus 174
JQ686833, Ngewotan negevirus (JKT9982)
OL700073, Manglie virus (XY54847)
KX903301, Castlerea virus (P59341)
ON949947, Utsjoki negevirus 1 (FIN/L-2018/90)
ON955085, Utsjoki negevirus 1 (FIN/L-2018/03-1)
ON949946, Utsjoki negevirus 1 (FIN/L-2018/85)
MT522375, Mekrijarvi negevirus (P39)
KX518821, San Bernardo virus (CoB52)
KM350509, Brejeira virus (AR800212-B)
KX518762, Brejeira virus (BeAr803410)
JQ675607, Piura virus (P60)
KX518750, Big cypress virus (BCNP2-24 A)
KX883760, Hubei negev-like virus 2 (CC64601)
JQ675610, Loreto virus (3940-83)
JQ675612, Loreto virus (Pe_AR_2617/77)
JQ675611, Loreto virus (Pe_AR_2612/77)
MT344120, dungfly Nelorpivirus (dungfly-1)
LC516834, Wuhan house centipede virus 1 (01BaA1)
LC516835, Barley aphid RNA virus 1 (02BaA3)
MZ615320, Cordoba virus (BeAr805503/CDBV)
GCYB01060845, Lygaeus turcicus breed wildtype TSA (s14933_L_73402_0_38_8)
JQ675604, Dezidougou virus (ArA 20086)
KY968698, Dezidougou virus (DEZI/Aedes_africanus/SEN/DAK-AR-41524/1984)
OP264896, Dezidougou virus (Mediterranean 2015)
MT096525, Dezidougou virus (FTA2-3)
OP576003, Dezidougou virus (8345)
ON949943, Dezidougou virus(FIN/PP-2018/82)
ON949944, Kustavi negevirus (FIN/VS-2018/100)
OQ749782, Wallerfield virus (Ar867255/WALV)
MH719095, Uxmal virus (UXMV-M985)
KM249339, Goutanap virus (C68-CI-2004)
KX518757, Biratnagar virus (Nepal12-1)
LC103139, Bustos virus (PL42)
KF425261, Tanay virus (11-2)
JQ675606, Santana virus (BeAR 517449)
PQ521674, TC-nege-like virus 1 (MR233-17E/290)
MG781153, Blunervirus camelliae, tea plant necrotic ring blotch virus
MK517478, Blunervirus solani, tomato fruit blotch virus (Fondi2018)
JN651149, Blunervirus vaccinii, blueberry necrotic ring blotch virus
DQ352194, Cilevirus leprosis, citrus leprosis virus C (Cordeiropolis)
DQ388512, Cilevirus leprosis, cytoplasmic citrus leprosis virus
JX000024, Cilevirus colombiaense, citrus leprosis virus C2 (L147V1)
MK804171, Cilevirus passiflora, passion fruit green spot virus (PFGSV/Snp1)
OK626451, Cilevirus australis, ligustrum leprosis virus (Cdb1)
OK626447, Cilevirus ligustri, Ligustrum chlorotic spot virus (SPa1)
OK626439, Cilevirus violifolium, Solanum violifolium ringspot virus (Prb1)
MT472637, Cilevirus oahuense, Hibiscus yellow blotch virus (OUGC)
HQ852052, Higrevirus waimanolo, hibiscus green spot virus 2 (WAI 1-1)
OR161045, Higrevirus waimanalo (DMs01)
OP324809, Phellodendron-associated higre-like virus PhAHLV-HBA
LC516836, Barley aphid RNA virus 2 (03BaA3)
LC516838, Barley aphid RNA virus 4 (05BaA1)
LC516837, Barley aphid RNA virus 3 (04BaA1)
LT221868, Idaeovirus ligustri, privet leaf blotch-associated virus
S51557, Idaeovirus rubi, raspberry bushy dwarf virus (RBDV R15)
0.5
Proposed name
"Nelorpivirus" (canonical RdRp)
Proposed name
"Sandewavirus" (permuted RdRp)
Kitaviridae
Kitaviridae
Mayoviridae
Martellivirales

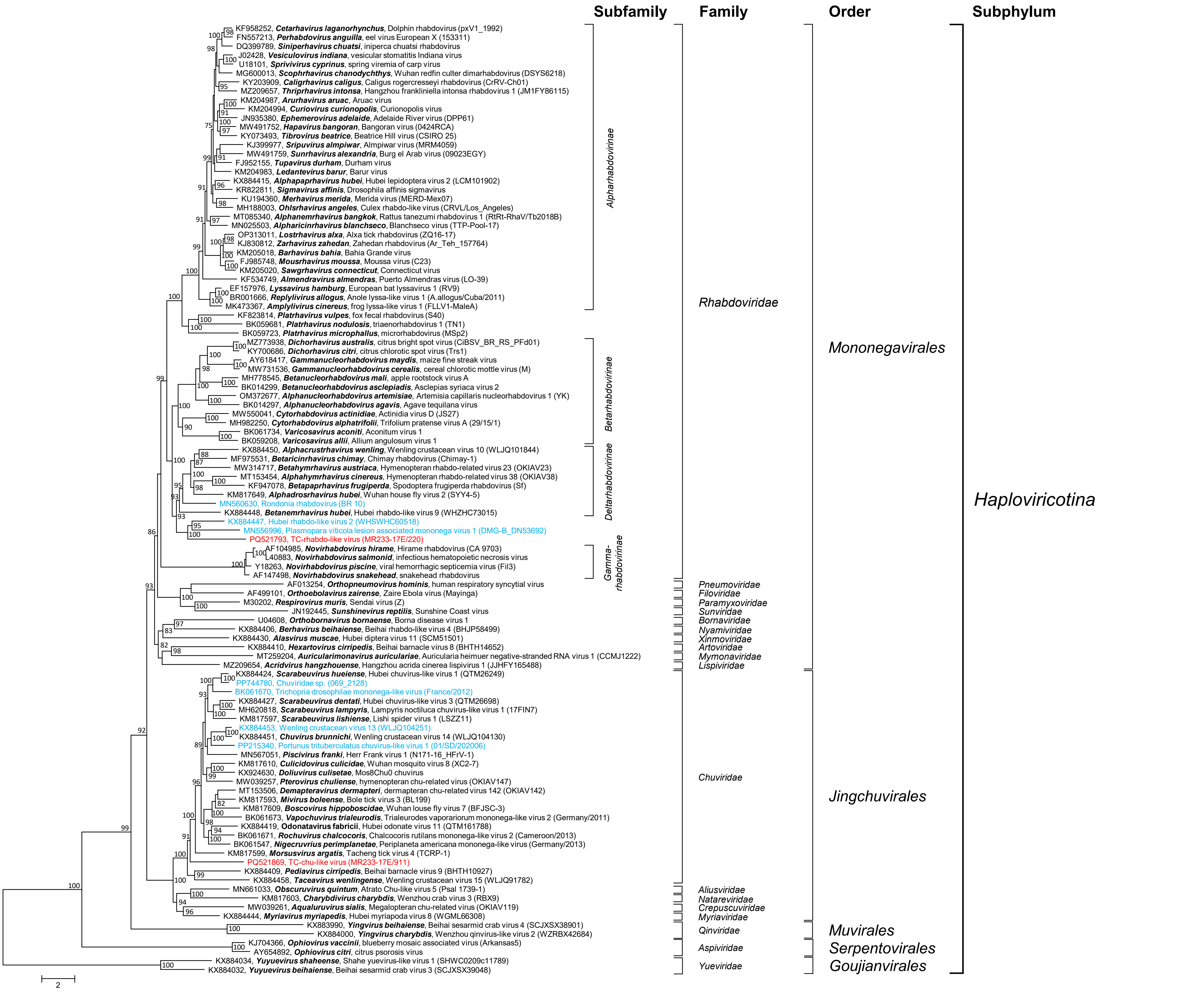
Subfamily
Family
Order
Subphylum
Alpharhabdovirinae
Betarhabdovirinae
Deltarhabdovirinae
Gamma-rhabdovirinae
Rhabdoviridae
Pneumoviridae
Filoviridae
Paramyxoviridae
Sunviridae
Bornaviridae
Nyamiviridae
Xinmoviridae
Artoviridae
Mymonaviridae
Lispiviridae
Chuviridae
Aliusviridae
Natareviridae
Crepuscuviridae
Myriaviridae
Qinviridae
Aspiviridae
Yueviridae
Mononegavirales
Jingchuvirales
Muvirales
Serpentovirales
Goujianvirales
Haploviricotina
KF958252, Cetarhavirus laganorhynchus, Dolphin rhabdovirus (pxV1_1992)
FN557213, Perhabdovirus anguilla, eel virus European X (153311)
DQ399789, Siniperhavirus chuatsi, iniperca chuatsi rhabdovirus
J02428, Vesiculovirus indiana, vesicular stomatitis Indiana virus
U18101, Sprivivirus cyprinus, spring viremia of carp virus
MG600013, Scophrhavirus chanodychthys, Wuhan redfin culter dimarhabdovirus (DSYS6218)
KY203909, Caligrhavirus caligus, Caligus rogercresseyi rhabdovirus (CrRV-Ch01)
MZ209657, Thriprhavirus intonsa, Hangzhou frankliniella intonsa rhabdovirus 1 (JM1FY86115)
KM204987, Arurhavirus aruac, Aruac virus
KM204994, Curiovirus curionopolis, Curionopolis virus
JN935380, Ephemerovirus adelaide, Adelaide River virus (DPP61)
MW491752, Hapavirus bangoran, Bangoran virus (0424RCA)
KY073493, Tibrovirus beatrice, Beatrice Hill virus (CSIRO 25)
KJ399977, Sripuvirus almpiwar, Almpiwar virus (MRM4059)
MW491759, Sunrhavirus alexandria, Burg el Arab virus (09023EGY)
FJ952155, Tupavirus durham, Durham virus
KM204983, Ledantevirus barur, Barur virus
KX884415, Alphapaprhavirus hubei, Hubei lepidoptera virus 2 (LCM101902)
KR822811, Sigmavirus affinis, Drosophila affinis sigmavirus
KU194360, Merhavirus merida, Merida virus (MERD-Mex07)
MH188003, Ohlsrhavirus angeles, Culex rhabdo-like virus (CRVL/Los_Angeles)
MT085340, Alphanemrhavirus bangkok, Rattus tanezumi rhabdovirus 1 (RtRt-RhaV/Tb2018B)
MN025503, Alpharicinrhavirus blanchseco, Blanchseco virus (TTP-Pool-17)
OP313011, Lostrhavirus alxa, Alxa tick rhabdovirus (ZQ16-17)
KJ830812, Zarhavirus zahedan, Zahedan rhabdovirus (Ar_Teh_157764)
KM205018, Barhavirus bahia, Bahia Grande virus
FJ985748, Mousrhavirus moussa, Moussa virus (C23)
KM205020, Sawgrhavirus connecticut, Connecticut virus
KF534749, Almendravirus almendras, Puerto Almendras virus (LO-39)
EF157976, Lyssavirus hamburg, European bat lyssavirus 1 (RV9)
BR001666, Replylivirus allogus, Anole lyssa-like virus 1 (A.allogus/Cuba/2011)
MK473367, Amplylivirus cinereus, frog lyssa-like virus 1 (FLLV1-MaleA)
KF823814, Platrhavirus vulpes, fox fecal rhabdovirus (S40)
BK059681, Platrhavirus nodulosis, triaenorhabdovirus 1 (TN1)
BK059723, Platrhavirus microphallus, microrhabdovirus (MSp2)
MZ773938, Dichorhavirus australis, citrus bright spot virus (CiBSV_BR_RS_PFd01)
KY700686, Dichorhavirus citri, citrus chlorotic spot virus (Trs1)
AY618417, Gammanucleorhabdovirus maydis, maize fine streak virus
MW731536, Gammanucleorhabdovirus cerealis, cereal chlorotic mottle virus (M)
MH778545, Betanucleorhabdovirus mali, apple rootstock virus A
BK014299, Betanucleorhabdovirus asclepiadis, Asclepias syriaca virus 2
OM372677, Alphanucleorhabdovirus artemisiae, Artemisia capillaris nucleorhabdovirus 1 (YK)
BK014297, Alphanucleorhabdovirus agavis, Agave tequilana virus
MW550041, Cytorhabdovirus actinidiae, Actinidia virus D (JS27)
MH982250, Cytorhabdovirus alphatrifolii, Trifolium pratense virus A (29/15/1)
BK061734, Varicosavirus aconiti, Aconitum virus 1
BK059208, Varicosavirus allii, Allium angulosum virus 1
KX884450, Alphacrustrhavirus wenling, Wenling crustacean virus 10 (WLJQ101844)
MF975531, Betaricinrhavirus chimay, Chimay rhabdovirus (Chimay-1)
MW314717, Betahymrhavirus austriaca, Hymenopteran rhabdo-related virus 23 (OKIAV23)
MT153454, Alphahymrhavirus cinereus, Hymenopteran rhabdo-related virus 38 (OKIAV38)
KF947078, Betapaprhavirus frugiperda, Spodoptera frugiperda rhabdovirus (Sf)
KM817649, Alphadrosrhavirus hubei, Wuhan house fly virus 2 (SYY4-5)
MN560630, Rondonia rhabdovirus (BR 10)
KX884448, Betanemrhavirus hubei, Hubei rhabdo-like virus 9 (WHZHC73015)
KX884447, Hubei rhabdo-like virus 2 (WHSWHC60518)
MN556996, Plasmopara viticola lesion associated mononega virus 1 (DMG-B_DN53692)
PQ521793, TC-rhabdo-like virus (MR233-17E/220)
AF104985, Novirhabdovirus hirame, Hirame rhabdovirus (CA 9703)
L40883, Novirhabdovirus salmonid, infectious hematopoietic necrosis virus
Y18263, Novirhabdovirus piscine, viral hemorrhagic septicemia virus (Fil3)
AF147498, Novirhabdovirus snakehead, snakehead rhabdovirus
AF013254, Orthopneumovirus hominis, human respiratory syncytial virus
AF499101, Orthoebolavirus zairense, Zaire Ebola virus (Mayinga)
M30202, Respirovirus muris, Sendai virus (Z)
JN192445, Sunshinevirus reptilis, Sunshine Coast virus
U04608, Orthobornavirus bornaense, Borna disease virus 1
KX884406, Berhavirus beihaiense, Beihai rhabdo-like virus 4 (BHJP58499)
KX884430, Alasvirus muscae, Hubei diptera virus 11 (SCM51501)
KX884410, Hexartovirus cirripedis, Beihai barnacle virus 8 (BHTH14652)
MT259204, Auricularimonavirus auriculariae, Auricularia heimuer negative-stranded RNA virus 1 (CCMJ1222)
MZ209654, Acridvirus hangzhouense, Hangzhou acrida cinerea lispivirus 1 (JJHFY165488)
KX884424, Scarabeuvirus hueiense, Hubei chuvirus-like virus 1 (QTM26249)
PP744780, Chuviridae sp. (069_2128)
BK061670, Trichopria drosophilae mononega-like virus (France/2012)
KX884427, Scarabeuvirus dentati, Hubei chuvirus-like virus 3 (QTM26698)
MH620818, Scarabeuvirus lampyris, Lampyris noctiluca chuvirus-like virus 1 (17FIN7)
KM817597, Scarabeuvirus lishiense, Lishi spider virus 1 (LSZZ11)
KX884453, Wenling crustacean virus 13 (WLJQ104251)
KX884451, Chuvirus brunnichi, Wenling crustacean virus 14 (WLJQ104130)
PP215340, Portunus trituberculatus chuvirus-like virus 1 (01/SD/202006)
MN567051, Piscivirus franki, Herr Frank virus 1 (N171-16_HFrV-1)
KM817610, Culicidovirus culicidae, Wuhan mosquito virus 8 (XC2-7)
KX924630, Doliuvirus culisetae, Mos8Chu0 chuvirus
MW039257, Pterovirus chuliense, hymenopteran chu-related virus (OKIAV147)
MT153506, Demapteravirus dermapteri, dermapteran chu-related virus 142 (OKIAV142)
KM817593, Mivirus boleense, Bole tick virus 3 (BL199)
KM817609, Boscovirus hippoboscidae, Wuhan louse fly virus 7 (BFJSC-3)
BK061673, Vapochuvirus trialeurodis, Trialeurodes vaporariorum mononega-like virus 2 (Germany/2011)
KX884419, Odonatavirus fabricii, Hubei odonate virus 11 (QTM161788)
BK061671, Rochuvirus chalcocoris, Chalcocoris rutilans mononega-like virus 2 (Cameroon/2013)
BK061547, Nigecruvirus perimplanetae, Periplaneta americana mononega-like virus (Germany/2013)
KM817599, Morsusvirus argatis, Tacheng tick virus 4 (TCRP-1)
PQ521869, TC-chu-like virus (MR233-17E/911)
KX884409, Pediavirus cirripedis, Beihai barnacle virus 9 (BHTH10927)
KX884458, Taceavirus wenlingense, Wenling crustacean virus 15 (WLJQ91782)
MN661033, Obscuruvirus quintum, Atrato Chu-like virus 5 (Psal 1739-1)
KM817603, Charybdivirus charybdis, Wenzhou crab virus 3 (RBX9)
MW039261, Aqualuruvirus sialis, Megalopteran chu-related virus (OKIAV119)
KX884444, Myriavirus myriapedis, Hubei myriapoda virus 8 (WGML66308)
KX883990, Yingvirus beihaiense, Beihai sesarmid crab virus 4 (SCJXSX38901)
KX884000, Yingvirus charybdis, Wenzhou qinvirus-like virus 2 (WZRBX42684)
KJ704366, Ophiovirus vaccinii, blueberry mosaic associated virus (Arkansas5)
AY654892, Ophiovirus citri, citrus psorosis virus
KX884034, Yuyuevirus shaheense, Shahe yuevirus-like virus 1 (SHWC0209c11789)
KX884032, Yuyuevirus beihaiense, Beihai sesarmid crab virus 3 (SCJXSX39048)
2

Table S1: Compilation of Teltow Canal and Havel River viruses

GenBank Acc. No.	Virus name	Length	Completeness	Mean depth	Strain designation	Genome layout, conserved domains
PQ521543	Havel bunya-like virus 1	6618 nt	par	40.4473	MR644-18E/103	orf-par: Acyl transferase domain in polyketide synthase enzymes
PQ521544	Havel bunya-like virus 2	4753 nt	par	58.4967	MR644-18E/199	orf-par: RdRp
PQ521545	Havel Jingmen-like virus (segment 1)	2978 nt	complete cds.	252.687	MR644-18E/486	orf1: Cap-O-specific (nucleoside-2'-O-)-methyltransferase/FtsJ – RdRp + orf2
PQ521546	Havel Jingmen-like virus (segment 3)	2732 nt	complete cds.	213.927	MR644-18E/591	orf: superfamily 2 helicase
PQ521547	Havel Jingmen-like virus (segment 4)	2750 nt	complete cds.	346.792	MR644-18E/582	orf1 + orf2: TonB
PQ521548	Havel noda-like virus 1	5009 nt	par	804.707	MR644-18E/174	bipartite?; orf1-par: Vmethyltr – RdRp + orf2
PQ521549	Havel noda-like virus 2	4795 nt	complete cds.	45.3639	MR644-18E/196	bipartite?; orf1-par: Vmethyltr – RdRp + orf2
PQ521550	Havel noda-like virus 3	4713 nt	complete cds.	23.6658	MR644-18E/201	bipartite?; orf1-par: Vmethyltr – RdRp + orf2
PQ521551	Havel noda-like virus 4	4581 nt	complete cds.	40.5339	MR644-18E/213	bipartite?; orf1-par: Vmethyltr – RdRp – Zn-finger + orf2
PQ521552	Havel noda-like virus 5	4483 nt	complete cds.	70.0466	MR644-18E/219	bipartite?; orf1-par: Vmethyltr – RdRp – Zn-finger + orf2
PQ521553	Havel noda-like virus 6	4463 nt	complete cds.	77.922	MR644-18E/223	bipartite?; orf1-par: Vmethyltr – RdRp + orf2
PQ521554	Havel noda-like virus 7	4443 nt	par	15.5501	MR644-18E/226	bipartite?; orf1-par: Vmethyltr – RdRp + orf2
PQ521555	Havel noda-like virus 8	3961 nt	par	106.197	MR644-18E/292	bipartite?; orf1-par: Vmethyltr – RdRp + orf2
PQ521556	Havel noda-like virus 9	3927 nt	par	17.5635	MR644-18E/299	orf-par: Vmethyltr – RdRp
PQ521557	Havel noda-like virus 10	3994 nt	par	10.2669	MR644-18E/371	dicistronic; orf1-par: Vmethyltr – RdRp + orf2-par: peptidase A6
PQ521558	Havel noda-like virus 11	3395 nt	par	12.9838	MR644-18E/405	orf-par: Vmethyltr – RdRp – hypothetical protein (PRK11901)
PQ521559	Havel noda-like virus 12	3359 nt	par	8.28193	MR644-18E/414	dicistronic; orf1-par: RdRp + orf2-par
PQ521560	Havel noda-like virus 13	3301 nt	complete cds.	456.884	MR644-18E/426	orf: Vmethyltr – RdRp
PQ521561	Havel noda-like virus 14	3148 nt	complete cds.	50.913	MR644-18E/455	orf: Vmethyltr – RdRp
PQ521562	Havel noda-like virus 15	3105 nt	complete cds.	54.2789	MR644-18E/463	orf: Vmethyltr – RdRp
PQ521563	Havel noda-like virus 16	3096 nt	par	123.256	MR644-18E/468	orf-par: Vmethyltr – RdRp
PQ521564	Havel noda-like virus 17	2713 nt	par	14.3564	MR644-18E/598	orf-par: Vmethyltr – RdRp
PQ521565	Havel noda-like virus 18	1676 nt	par	8.94451	MR644-18E/1617	orf-par: Vmethyltr – RdRp
PQ521566	Havel Riboviria sp.	2089 nt	par	12.6237	MR644-18E/1017	orf-par: peptidase A6
PQ521567	Havel Riboviria sp.	1914 nt	par	14.7194	MR644-18E/1234	dicistronic; orf1-par + orf2-par: peptidase A6
PQ521568	Havel Riboviria sp.	2198 nt	complete cds.	14.475	MR644-18E/2827	orf: peptidase A21
PQ521569	Havel Riboviria sp.	1253 nt	par	9.43575	MR644-18E/4758	orf-par: peptidase A21
PQ521570	Havel Riboviria sp.	1632 nt	par	9.5386	MR644-18E/9304	dicistronic; orf1-par + orf2-par: peptidase A21
PQ521862	Havel hepe-like virus	4418 nt	par	14.8015	MR644-18E/230	orf-par: FtsJ-like methyltransferase
PQ521571	Teltow Canal Riboviria sp.	2132 nt	complete cds.	443.111	MR233-17D/1798	orf: peptidase A6
PQ521572	Teltow Canal Riboviria sp.	2128 nt	complete cds.	895.14	MR233-17E/18595	orf: peptidase A6
PQ521573	Teltow Canal Riboviria sp.	1339 nt	complete cds.	30.4459	MR233-17E/24997	orf: peptidase A6
PQ521574	Teltow Canal Riboviria sp.	2109 nt	complete cds.	2501.95	MR233-17E/516	orf: peptidase A6
PQ521575	Teltow Canal Riboviria sp.	1684 nt	complete cds.	2278.27	MR233-17D/1485	orf: peptidase A6
PQ521576	Teltow Canal Riboviria sp.	1316 nt	par	29.6429	MR233-17D/4338	orf-par: peptidase A6
PQ521577	Teltow Canal Riboviria sp.	1453 nt	complete cds.	24.6242	MR233-17D/41700	orf: peptidase A6
PQ521578	Teltow Canal Riboviria sp.	1544 nt	complete cds.	6110.39	MR233-17E/15861	orf: peptidase A6
PQ521579	Teltow Canal Riboviria sp.	1543 nt	complete cds.	198.614	MR233-17E/15882	orf: peptidase A6
PQ521580	Teltow Canal Riboviria sp.	1537 nt	complete cds.	96.7437	MR233-17E/15998	orf: peptidase A6
PQ521581	Teltow Canal Riboviria sp.	1402 nt	complete cds.	42.2839	MR233-17E/19214	orf: peptidase A6
PQ521582	Teltow Canal Riboviria sp.	1393 nt	par	13.8241	MR233-17E/19479	orf-par: peptidase A6
PQ521583	Teltow Canal Riboviria sp.	1347 nt	par	12.4959	MR233-17E/20796	orf-par: peptidase A6
PQ521584	Teltow Canal Riboviria sp.	1340 nt	par	5.72612	MR233-17E/21028	orf-par: peptidase A6
PQ521585	Teltow Canal Riboviria sp.	1297 nt	complete cds.	45.0879	MR233-17E/22471	orf: peptidase A6
PQ521586	Teltow Conal Riboviria sp.	1352 nt	par	9.91864	MR233-17E/20659	orf-par: peptidase A6
PQ521587	Teltow Canal Riboviria sp.	1022 nt	par	6.02055	MR233-17E/36774	orf-par: peptidase A6
PQ521588	Teltow Canal Riboviria sp.	914 nt	par	6.60394	MR233-17E/46082	orf-par: peptidase A6
PQ521589	Teltow Canal Riboviria sp.	1709 nt	par	31.5108	MR233-17E/17942	orf-par: peptidase A21
PQ521590	Teltow Canal Riboviria sp.	1639 nt	par	10.3813	MR233-17E/14085	orf-par: peptidase A21
PQ521591	Teltow Canal Riboviria sp.	1421 nt	par	8.36242	MR233-17E/18730	orf-par: peptidase A21
PQ521592	Teltow Canal Riboviria sp.	1397 nt	par	12.6686	MR233-17E/19373	orf-par: peptidase A21
PQ521593	Teltow Canal Riboviria sp.	907 nt	par	3.19625	MR233-17E/46883	orf-par: peptidase A21
PQ521594	Teltow Canal Riboviria sp.	1268 nt	par	6.63407	MR233-17E/65621	orf-par: peptidase A21
PQ521595	Teltow Canal Riboviria sp.	754 nt	par	7.05703	MR233-17E/66408	orf-par: peptidase A21
PQ521596	Teltow Canal Riboviria sp.	1035 nt	par	7.69758	MR233-17E/69292	orf-par: peptidase A21
PQ521597	Teltow Canal Riboviria sp.	447 nt	par	4.39821	MR233-17E/174980	orf-par: peptidase A21
PQ521598	Teltow Canal Riboviria sp.	444 nt	par	5.60811	MR233-17E/177219	orf-par: peptidase A21
PQ521599	Teltow Canal Riboviria sp.	1682 nt	par	289.227	MR233-17E/8658	orf-par: viral coat protein S domain (pfam00729)
PQ521600	Teltow Canal Riboviria sp.	1360 nt	par	841.136	MR233-17D/1550	orf-par: viral coat protein S domain (pfam00729)
PQ521601	Teltow Canal Riboviria sp.	808 nt	par	2.43069	MR233-17D/12481	orf-par: viral coat protein S domain (pfam00729)
PQ521602	Teltow Canal Riboviria sp.	2066 nt	par	19.9758	MR233-17E/8894	orf1-par + orf2: viral coat protein S domain (pfam00729) **+** orf3-par

PQ521603	Teltow Canal Riboviria sp.	1859 nt	complete cds.	110678	MR233-17E/10966	orf: viral coat protein S domain (pfam00729)
PQ521604	Teltow Canal Riboviria sp.	1656 nt	par	318.773	MR233-17E/13799	orf-par: viral coat protein S domain (pfam00729)
PQ521605	Teltow Canal Riboviria sp.	1590 nt	par	795.935	MR233-17E/14945	orf-par: viral coat protein S domain (pfam00729)
PQ521606	Teltow Canal Riboviria sp.	1571 nt	complete cds.	25.9624	MR233-17E/15329	orf-par: viral coat protein S domain (pfam00729)
PQ521607	Teltow Canal Riboviria sp.	1445 nt	complete cds.	8.1308	MR233-17E/18124	orf-par: viral coat protein S domain (pfam00729)
PQ521608	Teltow Canal Riboviria sp.	1273 nt	par	15.6984	MR233-17E/23381	orf-par: viral coat protein S domain (pfam00729)
PQ521609	Teltow Canal Riboviria sp.	1208 nt	complete cds.	718.426	MR233-17E/25990	orf-par: viral coat protein S domain (pfam00729)
PQ521610	Teltow Canal Riboviria sp.	1195 nt	par	8.44435	MR233-17E/26622	orf-par: viral coat protein S domain (pfam00729)
PQ521611	Teltow Canal Riboviria sp.	951 nt	par	15.0568	MR233-17E/42563	orf-par: viral coat protein S domain (pfam00729)
PQ521612	Teltow Canal Riboviria sp.	696 nt	par	8.72845	MR233-17E/76895	orf-par: viral coat protein S domain (pfam00729)
PQ521868	Teltow Canal Riboviria sp.	1066 nt	par	4.12946	MR233-17E/33624	orf-par: viral coat protein S-domain (pfam00729)
PQ521613	Teltow Canal birna-like virus 1 (Segm. B)	4512 nt	par	16.3242	MR233-17E/1968	orf1-par + orf2: VP1 (RdRp)
PQ521614	Teltow Canal birna-like virus 2 (Segm. A)	3978 nt	par	619.044	MR233-17E/2515	orf1-par: VP5 + orf2: VP2/VP4/VP3
PQ521615	Teltow Canal birna-like virus 3 (Segm. A)	3959 nt	par	81.5916	MR233-17E/2536	orf1-par: VP5 + orf2: VP2/VP4/VP3
PQ521616	Teltow Canal birna-like virus 4 (Segm. B)	3851 nt	complete cds.	46.7372	MR233-17E/2693	orf: VP1 (RdRp)
PQ521617	Teltow Canal birna-like virus 5 (Segm B)	3837 nt	complete cds.	385.904	MR233-17E/2712	orf: VP1 (RdRp)
PQ521618	Teltow Canal birna-like virus 6 (Segm B)	3808 nt	complete cds.	382.575	MR233-17E/2763	orf: VP1 (RdRp)
PQ521619	Teltow Canal birna-like virus 7 (Segm. A)	3799 nt	complete cds.	2485.57	MR233-17E/2774	orf: VP2/VP4/VP3
PQ521620	Teltow Canal birna-like virus 8 (Segm. A)	3794 nt	complete cds.	52.4367	MR233-17E/2784	orf: VP2/VP4/VP3
PQ521621	Teltow Canal birna-like virus 9 (Segm. A)	3742 nt	complete cds.	65.2656	MR233-17E/2854	orf: VP2/VP4/VP3
PQ521622	Teltow Canal birna-like virus 10 (Segm. A)	3586 nt	complete cds.	466.363	MR233-17E/3001	orf: VP2/VP4/VP3
PQ521623	Teltow Canal birna-like virus 11 (Segm. B)	3474 nt	complete cds.	61.2948	MR233-17E/3342	orf: VP1 (RdRp)
PQ521624	Teltow Canal birna-like virus 12 (Segm. B)	3470 nt	complete cds.	80.5346	MR233-17E/3350	orf: VP1 (RdRp)
PQ521625	Teltow Canal birna-like virus 13 (Segm. B)	3446 nt	complete cds.	79.1773	MR233-17E/3409	orf: VP1 (RdRp)
PQ521626	Teltow Canal birna-like virus 14 (Segm. A)	3395 nt	par	73.1437	MR233-17E/3509	orf-par: VP2/VP4/VP3
PQ521627	Teltow Canal birna-like virus 15 (Segm. B)	3378 nt	complete cds.	91.5269	MR233-17E/3540	orf: VP1 (RdRp)
PQ521628	Teltow Canal birna-like virus 16 (Segm. A)	3309 nt	complete cds.	215.664	MR233-17E/3684	orf: VP2/VP4/VP3
PQ521629	Teltow Canal birna-like virus 17 (Segm. B)	3307 nt	complete cds.	288.008	MR233-17E/3688	orf: VP1 (RdRp)
PQ521630	Teltow Canal birna-like virus 18 (Segm. A)	2877 nt	par	10.2802	MR233-17E/4725	orf1: VP5 + orf2-par: VP2/VP4/VP3
PQ521631	Teltow Canal birna-like virus 19 (Segm. A)	2855 nt	complete cds.	22.6441	MR233-17E/4796	orf: VP2/VP4/VP3
PQ521632	Teltow Canal birna-like virus 20 (Segm. B)	2764 nt	complete cds.	55.9211	MR233-17E/5125	orf1: VP5 + orf2: VP2/VP4/VP3
PQ521633	Teltow Canal birna-like virus 21 (Segm. B)	3484 nt	complete cds.	3216.52	MR233-17D/318	orf: VP1 (RdRp)
PQ521634	Teltow Canal birna-like virus 22 (Segm. A)	3754 nt	par	28.4752	MR233-17D/60374	orf-par: VP2/VP4/VP3
PQ521635	Teltow Canal birna-like virus 23 (Segm. B)	1752 nt	par	13.4954	MR233-17E/12375	orf-par: VP1 (RdRp)
PQ521636	Teltow Canal birna-like virus 24 (Segm. A)	1539 nt	par	7.19168	MR233-17E/15968	orf-par: VP2/VP4/VP3
PQ521637	Teltow Canal birna-like virus 25 (Segm. A)	1535 nt	par	10.585	MR233-17E/16039	orf-par: VP2/VP4/VP3
PQ521638	Teltow Canal birna-like virus 26 (Segm. B)	1465 nt	par	10.6826	MR233-17E/17630	orf-par: VP1 (RdR)
PQ521639	Teltow Canal birna-like virus 27 (Segm. A)	1184 nt	par	10.9096	MR233-17E/27114	orf-par: VP2/VP4/VP3
PQ521640	Teltow Canal bunya-like virus 1	4943 nt	par	187.248	MR233-17E/1643	orf-par: RdRp
PQ521641	Teltow Canal bunya-like virus 2	2497 nt	par	11.6984	MR233-17E/6211	orf-par: RdRp
PQ521642	Teltow Canal bunya-like virus 3	1018 nt	par	8.34479	MR233-17E/37036	orf-par
PQ521643	Teltow Canal bunya-like virus 4	8397 nt	complete cds.	57.2382	MR233-17E/520	orf: RdRp
PQ521644	Teltow Canal bunya-like virus 5	7422 nt	par	30.5249	MR233-17E/700	orf: RdRp
PQ521645	Teltow Canal bunya-like virus 6	4591 nt	par	15.2557	MR233-17E/1905	orf: RdRp
PQ521646	Teltow Canal bunya-like virus 7	8078 nt	par	59.4237	MR233-17E/1974	orf: RdRp
PQ521647	Teltow Canal bunya-like virus 8	2554 nt	par	12.5948	MR233-17E/5956	orf: RdRp
PQ521648	Teltow Canal bunya-like virus 9	6487 nt	par	12.4265	MR233-17E/2107	orf1-par + orf2-par: RdRp
PQ521649	Teltow Canal bunya-like virus 10	2113 nt	par	12.1249	MR233-17E/8516	orf-par: RdRp
PQ521650	Teltow Canal bunya-like virus 11	1717 nt	par	10.9027	MR233-17E/12875	orf-par: RdRp
PQ521651	Teltow Canal bunya-like virus 12	1423 nt	par	9.95432	MR233-17E/18679	orf-par: RdRp
PQ521652	Teltow Canal bunya-like virus 13	1151 nt	par	6.33884	MR233-17E/28683	orf-par: RdRp
PQ521653	Teltow Canal bunya-like virus 14	8110 nt	par	723.257	MR233-17E/569	orf: RdRp
PQ521654	Teltow Canal bunya-like virus 15	1168 nt	par	10.0548	MR233-17E/63292	orf-par: RdRp
PQ521655	Teltow Canal bunya-like virus 16	946 nt	par	5.07082	MR233-17E/43037	orf-par
PQ521656	Teltow Canal bunya-like virus 17	905 nt	par	5.85635	MR233-17E/47041	orf-par: RdRp
PQ521657	Teltow Canal bunya-like virus 18	577 nt	par	7.24957	MR233-17E/108892	orf-par: RdRp
PQ521658	Teltow Canal bunya-like virus 19	9086 nt	complete cds.	24.5702	MR233-17E/368	orf: RdRp
PQ521659	Teltow Canal bunya-like virus 20	12413 nt	par	75.2144	MR233-17E/109	orf-par: RdRp
PQ521660	Teltow Canal bunya-like virus 21	8695 nt	complete cds.	70.9438	MR233-17E/457	orf: RdRp
PQ521661	Teltow Canal bunya-like virus 22	6418 nt	par	335.01	MR233-17E/941	orf-par: RdRp
PQ521662	Teltow Canal bunya-like virus 23	1744 nt	par	4.08773	MR233-17E/32850	orf-par: RdRp
PQ521663	Teltow Canal bunya-like virus 24	5104 nt	par	180.257	MR233-17E/1530	orf-par: RdRp
PQ521664	Teltow Canal bunya-like virus 25	9092 nt	par	32.1972	MR233-17E/365	orf-par: RdRp
PQ521665	Teltow Canal bunya-like virus 26	4911 nt	complete cds.	101.428	MR233-17E/1663	orf: RdRp

PQ521666	Teltow Canal bunya-like virus 27	5092 nt	complete cds.	16.5008	MR233-17E/1544	orf: RdRp
PQ521667	Teltow Canal bunya-like virus 28	3777 nt	par	20.2605	MR233-17E/2806	orf-par: RdRp
PQ521668	Teltow Canal bunya-like virus 29	4834 nt	complete cds.	221.101	MR233-17E/1724	orf: RdRp
PQ521861	Teltow Canal bunya-like virus 30	4219 nt	par	11.5783	MR233-17E/2243	orf-par: RdRp
PQ521869	Teltow Canal chu-like virus	6534 nt	par	9.6518	MR233-17E/911	orf-par: RdRp
PQ521669	Teltow Canal flavi-like virus	12630 nt	complete cds.	109.365	MR233-17E/147	orf: trypsin-like peptidase – superfamily 2 helicase - RdRp
PQ521670	Teltow Canal Jingmen-like virus (segm. 1)	3001 nt	complete cds.	11.9444	MR233-17E/31129	orf: NS5-like protein
PQ521671	Teltow Canal Jingmen-like virus (segm. 2)	2047 nt	complete cds.	22.6546	MR233-17E/9062	orf1 + orf2
PQ521672	Teltow Canal Jingmen-like virus (segm. 3)	733 nt	par	12.0041	MR233-17E/69945	orf-par: NS3-like protein
PQ521673	Teltow Canal Jingmen-like virus (segm. 4)	2742 nt	complete cds.	16.8968	MR233-17E/15544	orf1 + orf2: TonB
PQ521674	Teltow Canal nege-like virus 1	9403 nt	par	63.4208	MR233-17E/290	orf1: Vmethyltr – (nucleoside-2'-O-)-mehyltransferase – helicase – RdRp2 + orf2-par
PQ521675	Teltow Canal nege-like virus 2	1522 nt	par	8.68725	MR233-17E/16293	orf-par: RdRp2
PQ521676	Teltow Canal nege-like virus 3	886 nt	par	4.99669	MR233-17E/30650	orf-par: Vmethyltr
PQ521677	Teltow Canal nege-like virus 4	1196 nt	par	7.39967	MR233-17E/26563	orf-par: Vmethyltr
PQ521678	Teltow Canal nege-like virus 5	886 nt	par	5.64898	MR233-17E/49036	orf-par: RdRp2
PQ521679	Teltow Canal nido-like virus 1	14617 nt	par	21.7576	MR233-17E/70	orf1a + orf1b: RdRp – ZBD – Nsp13-like helicase – ExoN – m6A-methyltransferase + orf2 + orf3par
PQ521680	Teltow Canal nido-like virus 2	11436 nt	par	14.9325	MR233-17E/169	orf1-par: helicase – nsp14-like ExoN – NADAR + orf2-par
PQ521681	Teltow Canal nido-like virus 3	5105 nt	par	11.3459	MR233-17E/1529	orf-par: SAM-dep. methyltransferase – RdRp1 – ZBD
PQ521682	Teltow Canal nido-like virus 4	2960 nt	par	6.88581	MR233-17E/71601	orf-par: helicase – nsp14-like ExoN
PQ521683	Teltow Canal nido-like virus 5	1633 nt	par	7.30251	MR233-17E/14168	orf-par: RdRp
PQ521684	Teltow Canal nido-like virus 6	37031 nt	complete cds.	33.9878	MR233-17E/6	orf1a: ASCH + orf1b + orf2: SAM-dep. methyltransferase – RdRp – ZBD – helicase – nsp14-like ExoN – NADAR + orf3 + orf4
PQ521685	Teltow Canal nido-like virus 7	19043 nt	par	30.6177	MR233-17E/53	orf1-par + orf2: RdRp – ZBD – Hel – SAM-dep. methyltransferase + orf3 + orf4: protease + orf5 + orf6 + orf7-par
PQ521686	Teltow Canal nido-like virus 8	1135 nt	par	6.38855	MR233-17E/29485	orf-par: RdRp
PQ521687	Teltow Canal noda-like virus 1 (RNA1)	5543 nt	complete cds.	741.651	MR233-17E/1305	bipartite?; orf1: protein A (Vmethyltransferase – RdRp) + orf2: protein B2
PQ521688	Teltow Canal noda-like virus 2 (RNA1)	5619 nt	par	24.4868	MR233-17E/1386	bipartite?; orf-par: protein A (Vmethyltransferase – RdRp – Zn-finger)
PQ521689	Teltow Canal noda-like virus 3	5005 nt	par	81.1477	MR233-17E/1602	dicistronic; orf1-par: protein A (Vmethyltransferase – RdRp) + orf2
PQ521690	Teltow Canal noda-like virus 4	4584 nt	par	617.919	MR233-17E/1913	dicistronic; orf1-par: protein A (Vmethyltransferase – RdRp – Zn-finger) + orf2
PQ521691	Teltow Canal noda-like virus 5	4517 nt	par	25.1069	MR233-17E/1964	3 cistrons; orf1-par: protein A (Vmethyltransferase – RdRp) + orf2 + orf3
PQ521692	Teltow Canal noda-like virus 6	4441 nt	par	77.9333	MR233-17E/2023	dicistronic; orf1-par: protein A (Vmethyltransferase – RdRp) + orf2
PQ521693	Teltow Canal noda-like virus 7	4376 nt	par	35.1851	MR233-17E/2093	dicistronic; orf1-par: protein A (Vmethyltransferase – RdRp) + orf2
PQ521694	Teltow Canal noda-like virus 8	4249 nt	par	17.41	MR233-17E/2215	dicistronic; orf1-par: RdRp + orf2
PQ521695	Teltow Canal noda-like virus9 (RNA1)	1399 nt	par	11.0608	MR233-17E/19309	bipartite?; orf1-par: protein A (RdRp) + orf2: protein B2
PQ521696	Teltow Canal noda-like virus 10	3398 nt	par	172.856	MR233-17D/1070	dicistronic; orf1-par: Vmethyltransferase – RdRp + orf2-par: peptidase A6
PQ521697	Teltow Canal noda-like virus 11	3384 nt	par	406.417	MR233-17D/3384	dicistronic; orf1-par: Vmethyltransferase – RdRp + orf2: viral coat protein S domain
PQ521698	Teltow Canal noda-like virus 12 (RNA1)	3928 nt	par	156.804	MR233-17E/2583	bipartite?; orf1-par: protein A (Vmethyltransferase - RdRp) + orf2: protein B2
PQ521699	Teltow Canal noda-like virus 13	3906 nt	par	20.3628	MR233-17E/2619	dicistronic; orf1-par: Vmethyltransferase – RdRp + orf2
PQ521700	Teltow Canal noda-like virus 14	3629 nt	par	15.1681	MR233-17E/3052	dicistronic: orf1-par: Vmethyltransferase – RdRp + orf2-par
PQ521701	Teltow Canal noda-like virus 15 (RNA1)	3623 nt	par	82.7469	MR233-17E/3057	bipartite?; orf1-par: protein A (Vmethyltransferase – RdRp) + orf2: protein B2
PQ521702	Teltow Canal noda-like virus 16	3508 nt	complete cds.	236.409	MR233-17E/3279	dicistronic; orf1: Vmethyltransferase – RdRp + orf2: viral coat protein S domain
PQ521703	Teltow Canal noda-like virus 17 (RNA1)	3499 nt	par	293.28	MR233-17E/3292	bipartite?; orf1-par: protein A (Vmethyltransferase – RdRp) + orf2: protein B2
PQ521704	Teltow Canal noda-like virus 18 (RNA1)	3470 nt	complete cds.	17.4988	MR233-17E/3351	bipartite?; orf1: protein A (Vmethyltransferase – RdRp) + orf2: protein B2
PQ521705	Teltow Canal noda-like virus 19	4539 nt	par	16.759	MR233-17E/3389	orf-par: ubiquitin-like domain – RdRp
PQ521706	Teltow Canal noda-like virus 20 (RNA1)	3485 nt	complete cds.	32.3361	MR233-17E/3750	bipartite?; orf1: protein A (Vmethyltransferase – RdRp – Krüppel-like factor) + orf2: protein B2
PQ521707	Teltow Canal noda-like virus 21 (RNA1)	3251 nt	complete cds.	153.616	MR233-17E/3807	bipartite?; orf1: protein A (Vmethyltransferase – RdRp) + orf2: protein B2
PQ521708	Teltow Canal noda-like virus 22 (RNA1)	3191 nt	complete cds.	478.708	MR233-17E/3940	bipartite?; orf1: protein A (Vmethyltransferase – RdRp) + orf2: protein B2
PQ521709	Teltow Canal noda-like virus 23 (RNA1)	3166 nt	complete cds.	422.314	MR233-17E/3989	bipartite?; orf1: protein A (Vmethyltransferase – RdRp) + orf2: protein B2
PQ521710	Teltow Canal noda-like virus 24 (RNA1)	3145 nt	par	441.919	MR233-17E/4033	bipartite?; orf1-par: protein A (Vmethyltransferase – RdRp) + orf2: protein B2
PQ521711	Teltow Canal noda-like virus 25 (RNA1)	3127 nt	complete cds.	3881.05	MR233-17E/4081	bipartite?; orf1: protein A (Vmethyltransferase – RdRp) + orf2: protein B2
PQ521712	Teltow Canal noda-like virus 26 (RNA1)	3127 nt	complete cds.	125.412	MR233-17E/4082	bipartite?; orf1: protein A (Vmethyltransferase – RdRp) + orf2: protein B2
PQ521713	Teltow Canal noda-like virus 27 (RNA1)	3097 nt	par	57.5063	MR233-17E/4156	bipartite?; orf1-par: protein A (Vmethyltransferase – RdRp) + orf2: protein B2
PQ521714	Teltow Canal noda-like virus 28	2989 nt	par	58.0716	MR233-17E/4421	dcistronic; orf1-par: Vmethyltransferase – RdRp + orf2: hypothetical protein
PQ521715	Teltow Canal noda-like virus 29	4680 nt	par	17.5519	MR233-17E/4826	dicistronic; orf1-par: Vmethyltransferase – RdRp + orf2: hypothetical protein
PQ521716	Teltow Canal noda-like virus 30 (RNA1)	2483 nt	par	8.38462	MR233-17E/6283	bipartite?; orf1-par: protein A (Vmethyltransferase – RdRp) + orf2: protein B2
PQ521717	Teltow Canal noda-like virus 31 (RNA2?)	2482 nt	par	12.9029	MR233-17E/6288	bipartite?; orf1: capsid protein VNN + orf2-par: hypothetical protein
PQ521718	Teltow Canal noda-like virus 32	2368 nt	par	14.6558	MR233-17E/6869	dicistronic; orf1-par: hypothetical protein + orf2-par: hypothetical protein
PQ521719	Teltow Canal noda-like virus 33	2191 nt	par	7.79735	MR233-17E/7962	orf-par: protein A (Vmethyltransferase – RdRp)
PQ521720	Teltow Canal noda-like virus 34	2175 nt	par	12.2731	MR233-17E/8070	orf-par: protein A (Vmethyltransferase – RdRp)
PQ521721	Teltow Canal noda-like virus 35	2148 nt	par	13.3059	MR233-17E/8267	orf-par: protein A (Vmethyltransferase – RdRp)
PQ521722	Teltow Canal noda-like virus 36 (RNA1)	3233 nt	complete cds.	1410.18	MR233-17E/8376	bipartite?; orf1: protein A (Vmethyltransferase – RdRp) + orf2: protein B2
PQ521723	Teltow Canal noda-like virus 37	2098 nt	par	12.9194	MR233-17E/8624	orf-par: protein A (Vmethyltransferase – RdRp)
PQ521724	Teltow Canal noda-like virus 38 (RNA1)	3481 nt	complete cds.	72.1758	MR233-17D/27800	bipartite?; orf1: protein A (Vmethyltransferase – RdRp – Krüppel-like factor) + orf2: protein B2
PQ521725	Teltow Canal noda-like virus 39	2782 nt	par	153.05	MR233-17E/9549	dicistronic; orf1-par: RdRp + orf2: peptidase A6
PQ521726	Teltow Canal noda-like virus 40	1983 nt	par	18.3086	MR233-17D/9658	orf-par: protein A (Vmethyltransferase – RdRp)
PQ521727	Teltow Canal noda-like virus 41	1921 nt	par	4.41541	MR233-17E/10290	orf-par: RdRp

PQ521728	Teltow Canal noda-like virus 42	1909 nt	par	7.19853	MR233-17E/10430	orf-par: Vmethyltransferase
PQ521729	Teltow Canal noda-like virus 43	1905 nt	par	11.5974	MR233-17E/10484	orf-par: Vmethyltransferase – RdRp
PQ521730	Teltow Canal noda-like virus 44 (RNA1)	3243 nt	complete cds.	15.613	MR233-17E/10603	bipartite?; orf1: protein A (Vmethyltransferase – RdRp) + orf2: protein B2
PQ521731	Teltow Canal noda-like virus 45 (RNA1)	3034 nt	par	24.3725	MR233-17D/32087	bipartite?; orf1: protein A (Vmethyltransferase – RdRp) + orf2: protein B2
PQ521732	Teltow Canal noda-like virus 46	1849 nt	par	22.1401	MR233-17E/11094	orf-par: Vmethyltransferase – RdRp
PQ521733	Teltow Canal noda-like virus 47	1810 nt	par	26.9199	MR233-17E/11573	orf-par: capsid protein VNN
PQ521734	Teltow Canal noda-like virus 48	1832 nt	par	5.80731	MR233-17E/12504	orf-par: Vmethyltransferase – RdRp
PQ521735	Teltow Canal noda-like virus 49	1667 nt	par	7.40926	MR233-17E/13633	orf-par: protein A (RdRp)
PQ521736	Teltow Canal noda-like virus 50 (RNA1)	1635 nt	par	14.5987	MR233-17E/14142	bipartite?; orf1: protein A (Vmethyltransferase – RdRp) + orf2: protein B2
PQ521737	Teltow Canal noda-like virus 51	1751 nt	par	6.90634	MR233-17E/14651	dicistronic; orf1-par: RdRp + orf2: hypothetical protein
PQ521738	Teltow Canal noda-like virus 52	1602 nt	par	14.7514	MR233-17E/14707	orf-par: RdRp
PQ521739	Teltow Canal noda-like virus 53	1573 nt	par	13.9201	MR233-17E/15287	orf-par: RdRp
PQ521740	Teltow Canal noda-like virus 54	1335 nt	par	9.63221	MR233-17E/21193	orf-par: Vmethyltransferase
PQ521741	Teltow Canal noda-like virus 55	1925 nt	par	8.68831	MR233-17D/49524	orf-par: Vmethyltransferase – RdRp
PQ521742	Teltow Canal noda-like virus 56 (RNA1)	1746 nt	par	11.0487	MR233-17E/16291	bipartite?; orf1: protein A (Vmethyltransferase – RdRp) + orf2: protein B2
PQ521743	Teltow Canal noda-like virus 57	1498 nt	par	17.0834	MR233-17E/16810	orf-par: Vmethyltransferase
PQ521744	Teltow Canal noda-like virus 58	1494 nt	par	93.1867	MR233-17E/18829	orf-par: capsid protein VNN
PQ521745	Teltow Canal noda-like virus 59	1408 nt	par	21.7216	MR233-17D/416	orf-par: Vmethyltransferase
PQ521746	Teltow Canal noda-like virus 60 (RNA2?)	2219 nt	par	16.1469	MR233-17E/24280	bipartite?; orf1: capsid protein VNN + orf2-par: hypothetical protein
PQ521747	Teltow Canal noda-like virus 61	1031 nt	par	8.87876	MR233-17E/36069	orf-par: Vmethyltransferase
PQ521748	Teltow Canal noda-like virus 62	1024 nt	par	6.74609	MR233-17E/36597	orf-par: Vmethyltransferase
PQ521749	Teltow Canal noda-like virus 63	1016 nt	par	7.51772	MR233-17E/37182	orf-par
PQ521750	Teltow Canal noda-like virus 64	2800 nt	par	16.8375	MR233-17E/4996	orf-par: Vmethyltransferase – RdRp
PQ521751	Teltow Canal noda-like virus 65 (RNA1)	3108 nt	par	555.749	MR233-17E/4135	bipartite?; orf1-par: protein A (Vmethyltransferase – RdRp) + orf2: protein B2
PQ521752	Teltow Canal noda-like virus 66	419 nt	par	5.72554	MR233-17E/197940	orf-par: capsid protein VNN
PQ521753	Teltow Canal noda-like virus 67	1300 nt	par	4.91462	MR233-17E/22406	orf-par: Vmethyltransferase
PQ521754	Teltow Canal noda-like virus 68	1332 nt	par	11.717	MR233-17E/21291	orf-par: capsid protein VNN
PQ521755	Teltow Canal noda-like virus 69 (RNA1)	2398 nt	par	55.1884	MR233-17E/6690	bipartite?; orf1-par: protein A (Vmethyltransferase – RdRp) + orf2: protein B2
PQ521756	Teltow Canal noda-like virus 70 (RNA1)	2815 nt	complete cds.	9422.68	MR233-17E/4938	bipartite?; orf1-par: protein A (Vmethyltransferase – RdRp) + orf2: protein B2
PQ521757	Teltow Canal noda-like virus 71 (RNA2?)	2031 nt	par	16.6184	MR233-17E/9227	bipartite?; orf1-par: capsid protein VNN + orf2
PQ521758	Teltow Canal noda-like virus 72	2776 nt	par	24.5843	MR233-17E/5078	orf-par: Vmethyltransferase – RdRp
PQ521759	Teltow Canal noda-like virus 73 (RNA1)	2282 nt	par	57.1113	MR233-17E/787511	bipartite?; orf1-par: RdRp + orf2: protein B2
PQ521760	Teltow Canal noda-like virus 74	1139 nt	par	4.18437	MR233-17D/24203	orf-par: Vmethyltransferase
PQ521761	Teltow Canal noda-like virus 75	1274 nt	par	4.4427	MR233-17E/44659	orf-par: RdRp
PQ521762	Teltow Canal noda-like virus 76 (RNA1)	4627 nt	par	184.407	MR233-17E/1873	dicistronic; orf-par: Vmethyltransferase – RdRp + orf2
PQ521763	Teltow Canal noda-like virus 77	1948 nt	par	63.846	MR233-17D/15137	orf-par: Vmethyltransferase – RdRp
PQ521764	Teltow Canal noda-like virus 78 (RNA1)	3854 nt	par	31.7721	MR233-17E/2686	dicistronic; orf1-par: Vmethyltransferase – RdRp + orf2
PQ521765	Teltow Canal noda-like virus 79	1523 nt	par	13.0689	MR233-17E/16269	orf-par: capsid protein VNN
PQ521766	Teltow Canal noda-like virus 80	1959 nt	par	8.92853	MR233-17E/16843	orf-par: RdRp
PQ521767	Teltow Canal noda-like virus 81	2897 nt	par	62716.6	MR233-17E/4621	orf-par: RdRp
PQ521768	Teltow Canal noda-like virus 82	2869 nt	par	465.484	MR233-17E/4743	orf-par: Vmethyltransferase – RdRp
PQ521769	Teltow Canal noda-like virus 83	2740 nt	complete cds.	139.724	MR233-17E/5209	orf-par: Vmethyltransferase – RdRp
PQ521770	Teltow Canal noda-like virus 84	2735 nt	par	23.7507	MR233-17E/5229	orf-par: Vmethyltransferase – RdRp
PQ521771	Teltow Canal noda-like virus 85 (RNA1)	2825 nt	complete cds.	629.464	MR233-17E/4908	bipartite?; orf1-par: protein A (Vmethyltransferase – RdRp) + orf2: protein B2
PQ521772	Teltow Canal noda-like virus 86	3358 nt	complete cds.	510.027	MR233-17E/3569	orf: Vmethyltransferase – RdRp
PQ521773	Teltow Canal noda-like virus 88	1390 nt	par	115.681	MR233-17E/144186	orf-par: Vmethyltransferase
PQ521774	Teltow Canal noda-like virus 89 (RNA1)	2593 nt	par	25.7297	MR233-17E/6562	bipartite?; orf1-par: protein A (Vmethyltransferase – RdRp) + orf2: protein B2
PQ521775	Teltow Canal noda-like virus 90 (RNA1)	2672 nt	par	17.8911	MR233-17E/5487	bipartite?; orf1-par: protein A (Vmethyltransferase – RdRp) + orf2: protein B2
PQ521776	Teltow Canal noda-like virus 91 (RNA1)	3137 nt	par	38.5814	MR233-17E/11094	bipartite?; orf1-par: protein A (Vmethyltransferase – RdRp) + orf2: protein B2
PQ521777	Teltow Canal noda-like virus 92	4035 nt	par	53.0188	MR233-17E/2445	dicistronic: orf1-par: Vmethyltransferase – RdRp + orf2: peptidase A6
PQ521778	Teltow Canal noda-like virus 93	2708 nt	par	21.2253	MR233-17E/5351	orf-par: Vmethyltransferase – RdRp
PQ521779	Teltow Canal noda-like virus 94	1734 nt	par	27.7745	MR233-17D/36648	orf-par: Vmethyltransferase – RdRp
PQ521780	Teltow Canal noda-like virus 95 (RNA1)	3063 nt	complete cds.	17.4623	MR233-17E/4240	bipartite?; orf1-par: protein A (Vmethyltransferase – RdRp) + orf2: protein B2
PQ521781	Teltow Canal noda-like virus 96	2113 nt	par	20.8438	MR233-17E/8506	orf-par: Vmethyltransferase – RdRp
PQ521782	Teltow Canal noda-like virus 98	2756 nt	par	31.7384	MR233-17E/5152	orf-par: Vmethyltransferase – RdRp
PQ521783	Teltow Canal noda-like virus 99 (RNA1)	2847 nt	par	27.7583	MR233-17E/26292	bipartite?; orf1-par: protein A (Vmethyltransferase – RdRp) + orf2: protein B2
PQ521784	Teltow Canal noda-like virus 100	2044 nt	par	15.6375	MR233-17E/9097	orf-par: Vmethyltransferase – RdRp
PQ521785	Teltow Canal noda-like virus 101	4562 nt	par	1086.16	MR233-17D/5587	dicistronic: orf1-par: Vmethyltransferase – RdRp + orf2: coat protein S-domain
PQ521786	Teltow Canal noda-like virus 102	5538 nt	par	32.8906	MR233-17D/6837	dicistronic: orf1-par: Vmethyltransferase – RdRp + orf2
PQ521787	Teltow Canal noda-like virus 103	2813 nt	par	56.7295	MR233-17E/4950	orf1-par: Vmethyltransferase – RdRp + orf2: protein B2
PQ521788	Teltow Canal noda-like virus 104	2808 nt	par	14.9163	MR233-17E/4969	orf-par: Vmethyltransferase – RdRp
PQ521789	Teltow Canal noda-like virus 105	2341 nt	par	20.3148	MR233-17E/7003	orf-par: Vmethyltransferase – RdRp
PQ521790	Teltow Canal noda-like virus 106	2891 nt	par	118.488	MR233-17D/16355	orf-par: Vmethyltransferase – RdRp
PQ521791	Teltow Canal noda-like virus 107	1381 nt	par	13.1904	MR233-17E/19786	orf-par: Vmethyltransferase

PQ521792	Teltow Canal noda-like virus 108	2345 nt	par	18.2942	MR233-17E/6978	orf-par: Vmethyltransferase – RdRp
PQ521794	Teltow Canal permutotetra-like virus 1	4171 nt	complete cds.	2473.29	MR233-17E/2292	orf1: hypothetical CP + orf2: permuted RdRp
PQ521795	Teltow Canal permutotetra-like virus 2	4381 nt	complete cds.	82.2942	MR233-17E/2087	orf1: permuted RdRp + orf2: coat protein S-domain
PQ521796	Teltow Canal permutotetra-like virus 3	4179 nt	complete cds.	44.8354	MR233-17E/2278	orf1: hypothetical CP + orf2: permuted RdRp
PQ521797	Teltow Canal permutotetra-like virus 4	4149 nt	complete cds.	83.5086	MR233-17E/2311	orf1: permuted RdRp + orf2: hypothetical CP
PQ521798	Teltow Canal permutotetra-like virus 5	1496 nt	par	9.3389	MR233-17E/16863	orf-par: permuted RdRp
PQ521799	Teltow Canal permutotetra-like virus 6	1814 nt	par	4.09868	MR233-17D/160224	orf-par: permuted RdRp
PQ521800	Teltow Canal permutotetra-like virus 7	4035 nt	complete cds.	16.6791	MR233-17D/15195	orf1: permuted RdRp + orf2:
PQ521793	Teltow Canal rhabdo-like virus	10129 nt	complete cds.	30.6556	MR233-17E/220	orf1 + orf2 + orf3 + orf4 + orf5: L-protein (RdRp – mRNA-capping region)
PQ521801	Teltow Canal reo-like virus 1	1630 nt	par	6.57669	MR233-17E/14220	orf-par: FtsJ-like methyltransferase
PQ521802	Teltow Canal reo-like virus 2	4919 nt	complete cds.	108.605	MR233-17E/1657	orf: RdRp
PQ521803	Teltow Canal reo-like virus 3	4039 nt	complete cds.	359.257	MR233-17E/2440	orf: RdRp
PQ521804	Teltow Canal reo-like virus 4	3927 nt	complete cds.	395.036	MR233-17E/2735	orf: VP2-like
PQ521805	Teltow Canal reo-like virus 5	3822 nt	par	160.515	MR233-17E/2738	orf-par: VP2-like
PQ521806	Teltow Canal reo-like virus 6	3994 nt	complete cds.	761.31	MR233-17E/2745	orf: RdRp
PQ521807	Teltow Canal reo-like virus 7	2278 nt	par	20.0079	MR233-17E/7393	orf-par: RdRp
PQ521808	Teltow Canal reo-like virus 8	3623 nt	par	50.3895	MR233-17E/3058	orf-par: RdRp
PQ521809	Teltow Canal reo-like virus 9	4119 nt	par	26.6368	MR233-17E/4512	orf-par: RdRp
PQ521810	Teltow Canal reo-like virus 10	2634 nt	par	26.3345	MR233-17E/5628	orf-par: RdRp
PQ521811	Teltow Canal reo-like virus 11	2547 nt	par	30.9996	MR233-17E/5986	orf-par: RdRp
PQ521812	Teltow Canal reo-like virus 12	2514 nt	par	72.1014	MR233-17E/6133	orf-par: RdRp
PQ521813	Teltow Canal reo-like virus 13	1857 nt	par	24.2143	MR233-17E/10994	orf-par: VP4-like (methyltransferase)
PQ521814	Teltow Canal reo-like virus 14	1494 nt	par	18.8949	MR233-17E/16902	orf-par: RdRp
PQ521815	Teltow Canal reo-like virus 15	1394 nt	par	23.4971	MR233-17E/19446	orf-par: RdRp
PQ521816	Teltow Canal reo-like virus 16	1347 nt	par	8.69859	MR233-17E/20801	orf-par: RdRp
PQ521817	Teltow Canal reo-like virus 17	1966 nt	par	6.24313	MR233-17E/26273	orf-par: RdRp
PQ521818	Teltow Canal reo-like virus 18	1042 nt	par	20.5182	MR233-17E/35219	orf-par: RdRp
PQ521819	Teltow Canal reo-like virus 19	962 nt	par	16.9688	MR233-17E/41562	orf-par: RdRp
PQ521820	Teltow Canal reo-like virus 20	3429 nt	par	232.548	MR233-17E/3441	orf-par: RdRp
PQ521821	Teltow Canal reo-like virus 21	3444 nt	par	259.446	MR233-17E/3412	orf-par: RdRp
PQ521822	Teltow Canal reo-like virus 22	1985 nt	par	21.4514	MR233-17E/9640	orf-par
PQ521823	Teltow Canal rotavirus 1 (segm. 1)	1348 nt	par	38.0423	MR233-17E/20758	orf-par: VP1 (RdRp)
PQ521824	Teltow Canal rotavirus 2 (segm. 1)	1033 nt	par	7.20039	MR233-17E/35913	orf-par: VP1 (RdRp)
PQ521825	Teltow Canal rotavirus 3 (segm. 1)	1571 nt	par	46.4322	MR233-17E/15328	orf-par: VP1 (RdRp)
PQ521826	Teltow Canal rotavirus 4 (segm. 1)	1581 nt	par	52.1113	MR233-17E/15119	orf-par: VP1 (RdRp)
PQ521827	Teltow Canal rotavirus 5 (segm. 2)	1966 nt	par	48.3398	MR233-17E/13843	orf-par: VP2
PQ521828	Teltow Canal rotavirus 6 (segm. 2)	995 nt	par	36.6161	MR233-17E/38824	orf-par: VP2
PQ521829	Teltow Canal rotavirus 7 (segm. 2)	670 nt	par	73.1209	MR233-17E/82284	orf-par: VP2
PQ521830	Teltow Canal rotavirus 8 (segm. 3)	2568 nt	par	55.8777	MR233-17E/17468	orf-par: VP3 (Cap)
PQ521831	Teltow Canal rotavirus 9 (segm. 3)	2307 nt	par	59.9016	MR233-17E/7199	orf-par: VP3 (Cap)
PQ521832	Teltow Canal rotavirus 10 (segm. 3)	2164 nt	par	41.732	MR233-17E/12663	orf-par: VP3 (Cap)
PQ521833	Teltow Canal rotavirus 11 (segm. 3)	1074 nt	par	13.6387	MR233-17E/33095	orf-par: VP3 (Cap)
PQ521834	Teltow Canal rotavirus 12 (segm. 3)	723 nt	par	12.5505	MR233-17E/71695	orf-par: VP3 (Cap)
PQ521835	Teltow Canal rotavirus 13 (segm. 4)	2185 nt	par	67.3931	MR233-17E/10937	orf-par: VP4
PQ521836	Teltow Canal rotavirus 14 (segm. 4)	1804 nt	par	54.8038	MR233-17E/26818	orf-par: VP4
PQ521837	Teltow Canal rotavirus 15 (segm. 5)	1464 nt	par	96.9857	MR233-17E/17653	orf-par: NSP1
PQ521838	Teltow Canal rotavirus 16 (segm. 5)	1107 nt	par	5.20325	MR233-17E/73942	orf-par: NSP1
PQ521839	Teltow Canal rotavirus 17 (segm. 5)	951 nt	par	37.3701	MR233-17E/42549	orf-par: NSP1
PQ521840	Teltow Canal rotavirus 18 (segm. 6)	875 nt	par	104.286	MR233-17E/50179	orf-par: VP6
PQ521841	Teltow Canal rotavirus 19 (segm. 7)	1032 nt	par	218.721	MR233-17E/35947	orf-par: NSP3
PQ521842	Teltow Canal rotavirus 20 (segm. 7)	941 nt	par	94.8789	MR233-17E/43460	orf-par: NSP3
PQ521843	Teltow Canal rotavirus 21 (segm. 7)	880 nt	par	11.2591	MR233-17E/49626	orf-par: NSP3
PQ521844	Teltow Canal rotavirus 22 (segm. 8)	949 nt	par	67.9715	MR233-17E/42699	orf-par: NSP2
PQ521845	Teltow Canal rotavirus 23 (segm. 8)	938 nt	par	37.0075	MR233-17E/43759	orf-par: NSP2
PQ521846	Teltow Canal rotavirus 24 (segm. 8)	587 nt	par	9.57922	MR233-17E/105483	orf-par: NSP2
PQ521847	Teltow Canal rotavirus 25 (segm. 8)	566 nt	par	15.3551	MR233-17E/112852	orf-par: NSP2
PQ521848	Teltow Canal rotavirus 26 (segm. 9)	1034 nt	par	18.3656	MR233-17E/35790	orf-par: VP7
PQ521849	Teltow Canal rotavirus 27 (segm. 9)	739 nt	par	28.8309	MR233-17E/68847	orf-par: VP7
PQ521850	Teltow Canal rotavirus 28 (segm. 10)	525 nt	par	33.8171	MR233-17E/129296	orf-par: NSP4
PQ521851	Teltow Canal rotavirus 29 (segm. 11)	743 nt	par	20.4697	MR233-17E/68197	orf-par: NSP5/6
PQ521852	Teltow Canal seadornavirus (segm. 1)	3715 nt	par	155.899	MR233-17E/2901	orf-par: VP1
PQ521853	Teltow Canal seadornavirus (segm. 2)	3073 nt	par	86.699	MR233-17E/4214	orf-par: VP2
PQ521854	Teltow Canal seadornavirus (segm. 3)	2326 nt	complete cds.	322.87	MR233-17E/7089	orf: VP3
PQ521855	Teltow Canal seadornavirus (segm. 4)	1982 nt	par	56.1453	MR233-17E/6275	orf-par: VP4

PQ521856	Teltow Canal seadornavirus (segm. 5)	1606 nt	complete cds.	189.098	MR233-17E/14634	orf: VP5
PQ521857	Teltow Canal seadornavirus (segm. 6)	1567 nt	par	119.218	MR233-17E/15395	orf-par: VP6
PQ521858	Teltow Canal seadornavirus (segm. 7)	1153 nt	complete cds.	251.663	MR233-17E/28550	orf: VP7
PQ521859	Teltow Canal seadornavirus (segm. 8)	1014 nt	par	26.1765	MR233-17E/37302	orf-par: VP8
PQ521860	Teltow Canal seadornavirus (segm. 12)	731 nt	complete cds.	207.055	MR233-17E/70275	orf: VP12
PQ521863	Teltow Canal tombus-like virus 336	4367 nt	par	880.095	MR233-17E/2101	orf1a-par + orf1b: RdRp + orf2: peptidase A6
PQ521864	Teltow Canal tombus-like virus 337	3999 nt	par	8.54714	MR233-17E/2491	orf1a-par + orf1b: RdRp + orf2 + orf3: peptidase A21
PQ521865	Teltow Canal tombus-like virus 338	3957 nt	par	198.388	MR233-17D/1582	orf1a + orf1b: RdRp + orf2: coat protein S-domain
PQ521866	Teltow Canal tombus-like virus 339	4322 nt	complete cds.	88.2434	MR233-17E/2139	orf1a + orf1b: RdRp + orf2: peptidase A6
PQ521867	Teltow Canal tombus-like virus 340	4061 nt	par	26.8636	MR233-17E/2415	orf1a + orf1b: RdRp + orf2: peptidase A6
